# Association of Survival and Immune-Related Biomarkers With Immunotherapy in Patients With Non–Small Cell Lung Cancer: A Meta-analysis and Individual Patient–Level Analysis

**DOI:** 10.1001/jamanetworkopen.2019.6879

**Published:** 2019-07-10

**Authors:** Yunfang Yu, Dongqiang Zeng, Qiyun Ou, Shengbo Liu, Anlin Li, Yongjian Chen, Dagui Lin, Quanlong Gao, Haiyu Zhou, Wangjun Liao, Herui Yao

**Affiliations:** 1 Guangdong Provincial Key Laboratory of Malignant Tumor Epigenetics and Gene Regulation, Department of Oncology and Phase I Clinical Trial Centre, Sun Yat-sen Memorial Hospital, Sun Yat-sen University, Guangzhou, China; 2 Department of Oncology, Nanfang Hospital, Southern Medical University, Guangzhou, China; 3 Guangdong Key Laboratory for Research and Development of Natural Drugs, Guangdong Medical University, Zhanjiang, China; 4 Department of Thoracic Surgery, Guangdong General Hospital and Guangdong Academy of Medical Sciences, Southern Medical University, South China University of Technology, Guangzhou, China

## Abstract

**Importance:**

The beneficial role of immunotherapy and the clinical relevance of current biomarkers in non–small cell lung cancer (NSCLC) remain inconclusive; thus, appropriate strategies and reliable predictors need further definition.

**Objectives:**

To evaluate the association of clinical outcomes with immune checkpoint inhibitors, tumor vaccines, and cellular immunotherapy in patients with advanced NSCLC and to explore appropriate strategies, candidates, and predictors.

**Data Sources:**

The PubMed, EMBASE, and Cochrane Central Register of Controlled Trials databases were searched from inception to June 2018, using relevant search keywords and Medical Subject Headings (MeSH) terms, including *tumor vaccine*, *cellular immunotherapy*, *immune checkpoint inhibitor*, *cytotoxic T-lymphocyte-associated protein 4*, *programmed death-ligand 1*, *programmed death receptor 1*, and *non-small cell lung carcinoma*. Systematic reviews, meta-analyses, references, and conference proceedings were manually searched.

**Study Selection:**

English-language randomized clinical trials with available data that measured overall survival (OS), progression-free survival (PFS), or objective response rate comparing immune checkpoint inhibitors, tumor vaccines, or cellular immunotherapy with conventional therapy for patients with advanced or metastatic NSCLC were included. Thirty-one immunotherapy randomized clinical trials were included, and multicohort data included next-generation sequencing data from patients with advanced NCSLC.

**Data Extraction and Synthesis:**

Hazard ratios and 95% CIs were pooled to estimate the survival increases in OS and PFS. Dichotomous data, such as object response rate data, were analyzed using the risk ratio. Mantel-Haenszel random-effects model was used. *I*^2^ was used to assess the heterogeneity between trials; an *I*^2^ value exceeding 50% indicated the existence of substantial heterogeneity. Analyses took place from February 1, 2018, to August 31, 2018.

**Main Outcomes and Measures:**

Primary outcomes were OS and PFS.

**Results:**

In total, 14 395 patients (9500 [66.0%] men) were included in the meta-analysis, and 1833 patients (mean [SD], 65.2 [9.9] years; 1063 [58.0%] men) were included in the individual patient–level study. Compared with conventional therapy, immunotherapy was associated with significantly longer OS (hazard ratio, 0.76; 95% CI, 0.71-0.82; *P* < .001) and PFS (hazard ratio, 0.76; 95% CI, 0.70-0.83; *P* < .001). The best checkpoint blockade strategy was first-line pembrolizumab with platinum-based chemotherapy. The combined predictive utility of programmed cell death ligand 1 (PD-L1) expression and tumor mutation burden (TMB) was associated with predictive prognosis (whole-exome sequencing: 1-year PFS area under the receiver operating characteristic curve [AUC], 0.829; 3-year PFS AUC, 0.839; targeted next-generation sequencing: 1-year PFS AUC, 0.826; 3-year PFS AUC, 0.948). Moreover, the addition of CD8^+^ T-cell tumor-infiltrating lymphocytes was associated with improved prognosis predictions for OS (3-year OS AUC, 0.659; 5-year OS AUC, 0.665). *RYR1* or *MGAM* mutations were significantly associated with concomitantly increased durable clinical benefits (*RYR1*: durable clinical benefit [DCB], 12 of 51 patients [24%]; no durable benefit [NDB], 2 of 55 patients [4%]; *P* < .001; *MGAM*: DCB, 12 of 51 patients [24%]; NDB, 0 patients; *P* < .001), a higher TMB (*RYRI*: high TMB, 12 of 53 patients [23%]; low TMB, 2 of 53 patients [38%]; *P* < .001; *MGAM*: high TMB, 9 of 53 patients [17%]; low TMB, 0 patients; *P* < .001), and higher PD-L1 expression (*RYRI*: high PD-L1 expression, 8 of 30 patients [27%]; low PD-L1 expression, 6 of 85 [7.1%]; *P* < .001; *MGAM*: high PD-L1 expression, 6 of 30 patients [20%]; low PD-L1 expression, 5 of 85 patients [6%]; *P* < .001).

**Conclusions and Relevance:**

Immunotherapies showed promising clinical outcomes for patients with NSCLC. Pembrolizumab with platinum-based chemotherapy was found to be the most appropriate first-line immune checkpoint inhibitor regimen for advanced NSCLC, and the combined use of PD-L1 expression and TMB was found to be a promising biomarker to evaluate patients’ survival and response to precision immunotherapy. The further combination of CD8^+^ T-cell tumor-infiltrating lymphocytes, PD-L1 expression, and TMB was associated with reliable prognosis. The predictive value of that combination needs to be prospectively validated in large-scale studies.

## Key Points

**Question:**

Is immunotherapy associated with beneficial outcomes for advanced non–small cell lung cancer and how can combination strategies and biomarkers be deployed to produce the best benefit?

**Findings:**

In this meta-analysis including 14 395 patients and individual patient–level analysis including 1833 patients, immunotherapy was associated with improved survival, and pembrolizumab with platinum-based chemotherapy was identified as the best first-line checkpoint blockade strategy. Programmed cell death ligand 1 expression and tumor mutation burden jointly exhibited promising predictive and prognostic associations, and CD8^+^ T-cell tumor-infiltrating lymphocytes score further synergized with these 2 biomarkers.

**Meaning:**

Integrated tumor microenvironment-based biomarkers may be an effective means to predict responsiveness to immunotherapy for patients with advanced non–small cell lung cancer.

## Teaser

This meta-analysis examines whether differences in biomarkers, such as programmed cell death ligand 1 (PD-L1) expression and tumor mutation burden, are associated with improved response to immunotherapy and survival in patients with advanced non–small cell lung cancer (NSCLC).

## Introduction

Advances in immuno-oncology are changing the standard of care for non–small cell lung cancer (NSCLC) through immunotherapies, including tumor vaccines, cellular immunotherapies, and immune checkpoint inhibitors (ICIs), that aim to establish or enhance effective immune responses toward a tumor.^1,2^ However, immunotherapy has produced inconsistent results in previous randomized clinical trials (RCTs). In the KEYNOTE-024,^3^ CheckMate-057,^4^ and TIME^5^ trials and the study by Li et al,^6^ immunotherapies were found to significantly improve overall survival (OS) and progression-free survival (PFS) rates compared with chemotherapy in patients with advanced NSCLC, but inconsistent survival outcomes were shown in the CheckMate-026 trial^7^ and the studies by Takayama et al^8^ and Wu et al.^9^ Additionally, important questions remain regarding which immunotherapeutic strategy can be deployed to the best benefit.

Moreover, independent immune-related biomarkers that are currently used, such as programmed cell death ligand 1 (PD-L1)^10,11^ and tumor mutation burden (TMB),^12^ have achieved clinical relevance for a selection of patients to some extent, but to our knowledge, they are still far from clear and established,^13,14^ which warrants the development of an integrated tumor microenvironment–based signature with multiple parameters to maximize treatment effects and guide suitable strategies. In this meta-analysis and individual patient–level analysis, we describe the largest series to date, to our knowledge, to evaluate clinical outcomes, treatment strategies, exploratory patient subtypes, and predictive biomarkers in patients with NSCLC.

## Methods

A systematic literature search of the PubMed, EMBASE, and Cochrane Central Register of Controlled Trials databases was performed to identify relevant RCTs published from inception to June 2018, using search keywords and Medical Subject Headings (MeSH) terms pertinent to the intervention of interest, such as *tumor vaccine*, *cellular immunotherapy*, *immune checkpoint inhibitor*, *cytotoxic T-lymphocyte-associated protein 4*, *programmed death-ligand 1*, *programmed death receptor 1*, and *non–small cell lung carcinoma*. Furthermore, we manually searched and checked references of systematic reviews, meta-analyses, and conference proceedings of the American Society of Clinical Oncology, the European Society for Medical Oncology, the American Association for Cancer Research, and the World Conference on Lung Cancer. Database searches were conducted in January 2018 and updated in July 2018. The inclusion criteria were (1) RCTs comparing ICIs, tumor vaccines, or cellular immunotherapy with conventional therapy for patients with advanced or metastatic NSCLC; (2) RCTs with reported available data that measured OS, PFS, or objective response rate (ORR); and (3) RCTs published in English. The primary outcomes were OS and PFS; the secondary outcome was the ORR and durable clinical benefit (DCB). This study is reported following the Preferred Reporting Items for Systematic Reviews and Meta-analyses (PRISMA) reporting guideline (eFigure 1 in the Supplement).

### Statistical Analysis

For the meta-analysis, hazard ratios (HRs) and 95% CIs were pooled to estimate the survival increases in OS and PFS. Dichotomous data, such as ORR data, were analyzed using the risk ratio. The Mantel-Haenszel random-effects model was used. Two-sided *P* values less than .05 were regarded as statistically significant. We used *I*^2^ to assess the heterogeneity between trials; an *I*^2^ value exceeding 50% indicated substantial heterogeneity. The differences in treatment effect between subgroups were measured by *P* value for interaction. In addition, we conducted network meta-analyses to compare the OS and PFS of different ICI strategies using the random-eﬀects Bayesian model.

The GRADE (Grading of Recommendations Assessment, Development, and Evaluation) method was used to categorize the quality of the evidence as high, moderate, low, or very low. Randomized clinical trials were initially considered high-quality evidence but could be rated lower because of a risk of bias, imprecision, inconsistency, indirectness, and publication bias.

For individual patient–level analysis, aggregated OS and PFS were computed using the Kaplan-Meier estimates method and compared with the log-rank test. Hazard ratios and 95% CIs were calculated by using the Cox regression model. Treatment effects between 2 groups were calculated using the difference in restricted mean survival time. Categorical variables were compared with χ^2^ or Fisher exact tests, and continuous variables were compared with Wilcoxon rank sum tests for 2-group comparisons or the Kruskal-Wallis exact test for multiple comparisons. We categorized PD-L1, TMB, and the neoantigen burden (NAB) into high-value and low-value groups with the optimal cutoff values defined by the R statistical software version 3.4.1 ggsurvimier package (R Project for Statistical Computing). Tumor mutation burden was defined as the number of nonsynonymous single-nucleotide variants or insertion or deletion variants. Spearman rank correlation coefficients were used to estimate the correlations. Receiver operating characteristic curves were generated to assess the sensitivity and specificity of continuous variables with the area under the receiver operating characteristic curve (AUC), and a *P* value less than .05 was considered statistically significant. Analyses took place from February 1, 2018 to August 31, 2018. All statistical analyses were performed with R statistical software version 3.4.1. Full details of the methods are described in the eAppendix in the Supplement.

## Results

Our search found 31 relevant RCTs^3,4,5,6,7,8,9,10,11,12,15,16,17,18,19,20,21,22,23,24,25,26,27,28,29,30,31,32,33,34,35^ for meta-analyses of ICIs, tumor vaccines, or cellular immunotherapy, including 14 395 patients (9500 [66.0%] men) with advanced NSCLC. Next, we conducted an individual patient–level analysis by examining next-generation sequencing data for 1833 patients with NSCLC (mean [SD], 65.2 [9.9] years; 1063 [58.0%] men) in multicohort trials. A total of 825 patients who received ICIs were analyzed, including 349 patients in cohort 1 from 3 studies (the KEYNOTE-001 trial,^36^ the CheckMate-012 trial,^37^ and the study by Rizvi et al^38^), 56 patients in cohort 2 for OS (from cBioPortal for Cancer Genetics^39^), and 420 patients from the OAK trial.^10^ Additionally, 1008 patients were included from the Cancer Genome Atlas.^40^ The characteristics of the RCTs are summarized in eTable 1 in the Supplement, and characteristics of the ICI cohorts are summarized in eTable 2 in the Supplement. The results of the methodological quality analysis of the RCTs are summarized in eFigure 2 and eFigure 3 in the Supplement, and publication bias analyses of the RCTs are summarized eFigure 4 in the Supplement.

### Association of Immunotherapy With OS and PFS

**Figure 1.**
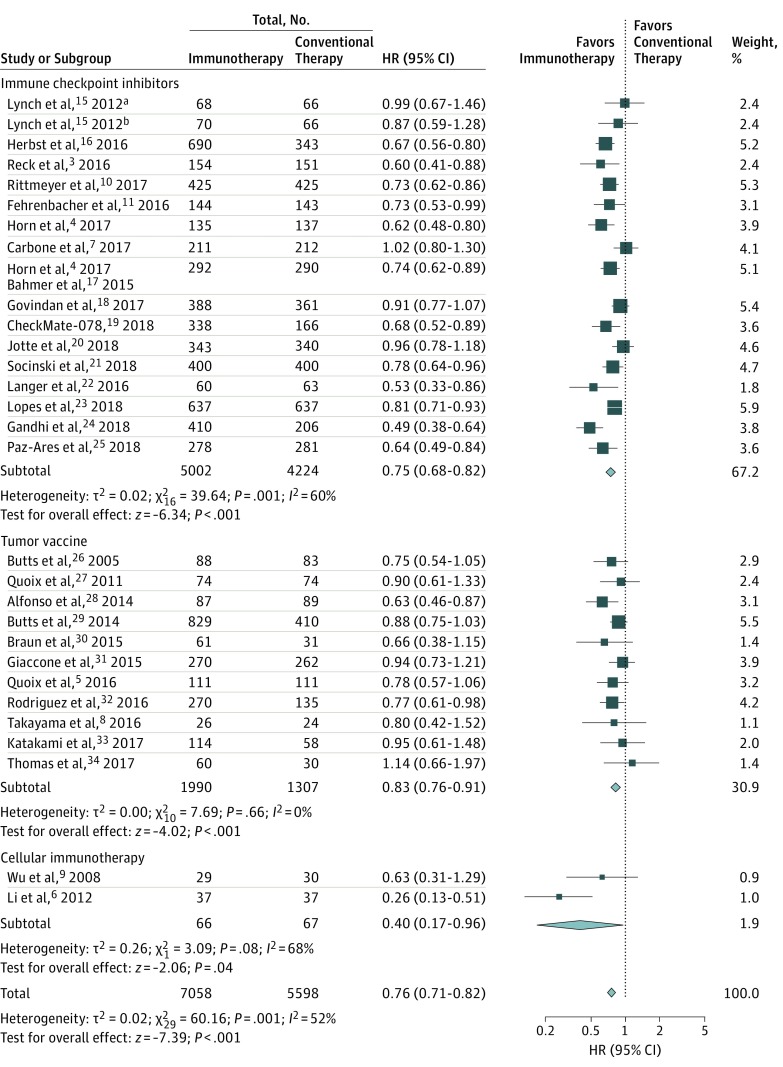
Pooled Hazard Ratios (HRs) for Overall Survival With Immunotherapy vs Conventional Therapy Size of boxes indicates proportional weight of each trial. Diamonds indicate point estimates and 95% CIs of the combined result. ^a^Patients were treated by phased regimen. ^b^Patients were treated by concurrent regimen.

Overall, compared with conventional therapy, immunotherapy was associated with significantly longer OS (HR, 0.76; 95% CI, 0.71-0.82; *P* < .001; ratio of the medians, 1.29; 95% CI, 1.14-1.46; *P* < .001) (Figure 1; eFigure 5 in the Supplement) and PFS (HR, 0.76; 95% CI, 0.71-0.82; *P* < .001; ratio of the medians, 1.15; 95% CI, 1.01-1.32; *P* = .04) (eFigure 6 and eFigure 7 in the Supplement). When classified by treatment, ICIs (HR, 0.75; 95% CI, 0.68-0.82; *P* < .001), tumor vaccines (HR, 0.83; 95% CI, 0.76-0.91; *P* < .001), and cellular immunotherapy (HR, 0.40; 95% CI, 0.17-0.96; *P* = .04) were associated with improved OS, while a significant improvement in PFS was recorded for ICIs (HR, 0.76; 95% CI, 0.68-0.84; *P* < .001) and tumor vaccines (HR, 0.86; 95% CI, 0.78-0.94; *P* = .001) (Figure 1; eFigure 6 in the Supplement). The GRADE evidence ranged from moderate to high quality.

Across the ICI RCTs, moderate-quality to high-quality evidence revealed significant improvements in PFS in first-line dual ICIs vs chemotherapy (HR, 0.83; 95% CI, 0.72-0.96; *P* = .01), first-line ICIs with chemotherapy vs chemotherapy alone (HR, 0.68; 95% CI, 0.58-0.80; *P* < .001), first-line ICIs with anti–vascular endothelial growth factor receptor therapy and chemotherapy vs anti–vascular endothelial growth factor receptor therapy with chemotherapy (HR, 0.61; 95% CI, 0.52-0.72; *P* < .001), first-line maintenance ICIs vs chemotherapy (HR, 0.52; 95% CI, 0.42-0.65; *P* < .001), and ICIs vs chemotherapy in patients who had been previously treated (HR, 0.85; 95% CI, 0.77-0.94; *P* = .002) (eTable 3 in the Supplement). Similar survival benefits were also recorded for OS (eTable 4 in the Supplement). Furthermore, comprehensive network meta-analysis showed that first-line pembrolizumab with platinum-based chemotherapy was superior to nivolumab, atezolizumab with platinum-based chemotherapy, and ipilimumab with platinum-based chemotherapy in OS and PFS. There were no survival differences among atezolizumab, nivolumab, and pembrolizumab in patients who had been previously treated (eFigures 8-14 in the Supplement).

In tumor vaccine trials, moderate-quality to high-quality evidence indicated that, compared with chemotherapy, first-line tumor vaccine immunotherapy with chemotherapy was associated with improved PFS (HR, 0.74; 95% CI, 0.60-0.91; *P* = .005). Compared with no vaccine treatment, first-line maintenance tumor vaccine immunotherapy was associated with improved OS (HR, 0.89; 95% CI, 0.81-0.99; *P* = .001) and PFS (HR, 0.83; 95% CI, 0.74-0.92; *P* = .02). More results are shown in eTable 3 and eTable 4 in the Supplement.

### Association of Immunotherapy With Response

The ORR was higher with immunotherapy than with conventional therapy (risk ratio, 1.33; 95% CI, 1.18-1.51; *P* < .001), and the benefit of objective response with ICIs was statistically significant (risk ratio, 1.47; 95% CI, 1.25-1.73; *P* < .001) (eFigure 15 in the Supplement). Furthermore, we performed trial sequential analysis on the ORR results and found a 20% relative risk increment in the response with immunotherapy (eFigure 16A in the Supplement) and specifically with ICIs (eFigure 16B in the Supplement). There was moderate-quality to high-quality evidence for a response benefit of first-line or second-line ICIs and first-line or maintenance tumor vaccines compared with conventional therapy (eTable 5 in the Supplement).

### Association of PD-L1 Expression, TMB, and NAB With OS and PFS Among Patients Treated With ICIs 

The subgroup analysis of meta-analysis showed that PFS was significantly longer with ICIs than with conventional therapy among patients with a high TMB but not among those with a low TMB. In addition, OS and PFS benefits from ICIs increased with increasing PD-L1 expression compared with conditional therapy. By comparing the HR of OS for ICIs alone vs chemotherapy alone (HR, 0.70; 95% CI, 0.61-0.79) and that of ICIs with chemotherapy vs chemotherapy alone (HR, 0.50; 95% CI, 0.38-0.66), we found that ICIs with chemotherapy were associated with significantly longer OS compared with ICIs alone in patients with PD-L1 expression scores of 1, 2, or 3 for tumor cells (TCs) (defined as the number of TCs expressing PD-L1 as a percentage of total TCs; TC1, ≥1% to <5%; TC2, ≥5% to <50%; TC3, ≥50%) or for tumor-infiltrating immune cells (ICs) (defined as the number of tumor-infiltrating ICs as a percentage of tumor area; IC1, ≥1% to <5%; IC2, ≥5% to <10%; IC3, ≥10%) (*P* for interaction = .03). However, the OS benefit of ICIs with chemotherapy compared with ICIs alone did not differ in patients with PD-L1 expression scores of TC3 or IC3 or for patients with PD-L1 expression scores TC2 or IC2. More results of subgroup analyses and the GRADE evidence are presented in eFigure 17, eFigure 18, eTable 6, and eTable 7 in the Supplement.

**Figure 2.**
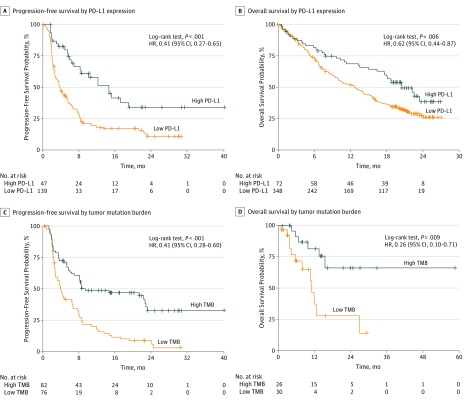
Survival Analysis of Patients Stratified by Programmed Cell Death Ligand 1 (PD-L1) Expression or Tumor Mutation Burden (TMB) A, Progression-free survival curves were plotted for patients stratified by PD-L1 expression in cohort 1. B, Overall survival curves were plotted for patients stratified by PD-L1 expression in the OAK trial.^10^ C, Progression-free survival curves were plotted for patients stratified by tumor mutation burden in cohort 1. D, Overall survival curves were plotted for patients stratified by tumor mutation burden in cohort 2. Crosses indicate censoring of data. HR indicates hazard ratio.

Individual patient–level analysis in the ICI cohorts found that PD-L1 expression, TMB, and the NAB were significantly greater in the patients with a complete response (defined as all target lesions have disappeared and any target or nontarget pathological lymph nodes have been reduced to <10 mm in the short axis) or partial response (defined as ≥30% decreased sum of diameters of target lesions compared with the baseline sum diameters) compared with patients with stable disease (defined as neither sufficient shrinkage to qualify for partial response nor sufficient increase to qualify for progressive disease, using the smallest sum diameters during the study as the reference) or progressive disease (defined as ≥20% increase in the sum diameters of target lesions, using the smallest sum during the study as reference, and the sum diameters demonstrate an absolute increase of ≥5 mm)^41^ (eFigure 19 in the Supplement). Likewise, these biomarkers were also greater in the patients with DCB than in the patients with no durable benefit (NDB) (eFigure 19 in the Supplement). Additionally, PFS was higher in the patients with high PD-L1 expression than in those with low PD-L1 expression (HR, 0.41; 95% CI, 0.27-0.65; *P* < .001) (Figure 2A). Similar results were observed when the patients were stratified into high TMB vs low TMB groups (HR, 0.41; 95% CI, 0.28-0.60; *P* < .001) (Figure 2C) as well as high NAB vs low NAB groups (HR, 0.34; 95% CI, 0.18-0.66; *P* < .001) (eFigure 20 in the Supplement). We found significantly improved OS associated with patients with high PD-L1 expression (HR, 0.62; 95% CI, 0.44-0.81; *P* = .006) or high TMB (HR, 0.26; 95% CI, 0.10-0.71; *P* = .009) (Figure 2B-D). The OS results were further confirmed by calculating the restricted mean survival time, which showed that the mean months of life gained through 24 months for high vs low PD-L1 expression was 3.36 (95% CI, 1.17-5.54) months (*P* = .003); for high vs low TMB, restricted mean survival time was 6.78 (95% CI, 1.75-11.81) months (*P* = .008). We also characterized PD-L1 expression, TMB, and the NAB in the Cancer Genome Atlas^40^ cohort and found consistent results (eFigure 21 in the Supplement).

**Figure 3.**
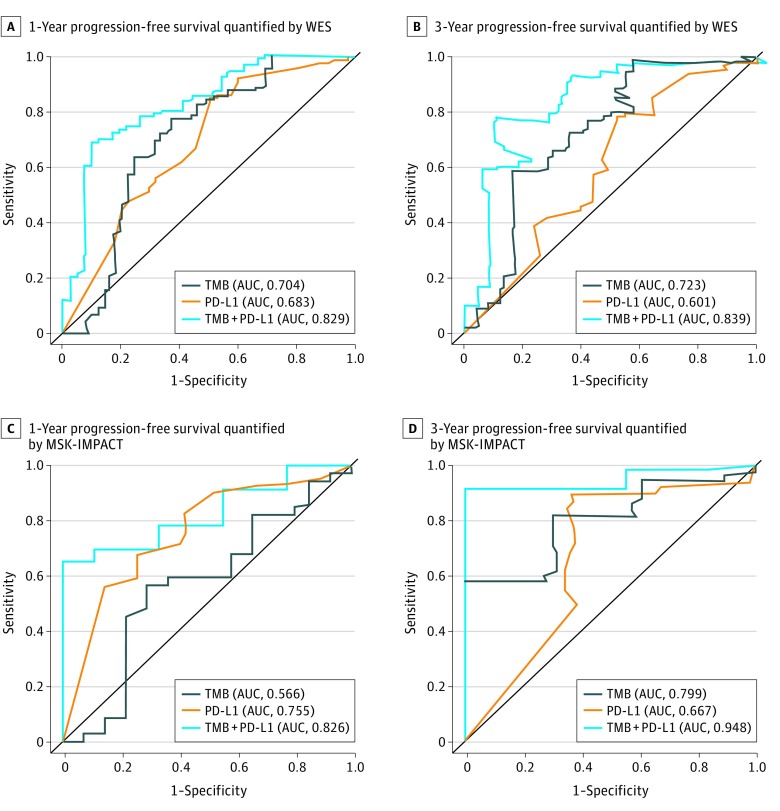
Joint Association of Programmed Cell Death Ligand 1 (PD-L1) Expression and Tumor Mutation Burden (TMB) With Survival in Checkpoint Blockade A and B, Patient tumor tissues were quantified by whole-exome sequencing (WES) and receiver operating characteristic curves were plotted for sensitivity vs 1 − specificity of 1-year progression-free survival (A) and 3-year progression-free survival (B). C and D, Patient tumor tissues were quantified by Memorial Sloan Kettering–Integrated Mutation Profiling of Actionable Cancer Targets (MSK-IMPACT) sequencing and receiver operating characteristic curves were plotted for sensitivity vs 1 − specificity of 1-year progression-free survival (C) and 3-year progression-free survival (D). AUC indicates area under the receiver operating characteristic curve.

There was an association of increased PD-L1 expression or an increased TMB with an increased rate of 1-year PFS (PD-L1: AUC, 0.683; TMB: AUC, 0.704), 3-year PFS (PD-L1: AUC, 0.601; TMB: AUC, 0.723), complete response or partial response (PD-L1: AUC, 0.643; TMB: AUC, 0.727), and DCB (PD-L1: AUC, 0.621; TMB: AUC, 0.679) in cohort 1 (Figure 3A and B; eFigure 22 in the Supplement).

### Association of Genomic Biomarker Combinations With ICI Response

We next evaluated the joint use of the 2 biomarkers in predicting response and PFS. A strong correlation was found between TMB and NAB (Spearman ρ = 0.78; *P* < .001). No correlation was found between PD-L1 expression and TMB (eFigure 23 in the Supplement), which suggests that PD-L1 expression and TMB were independent predictive measures of immunotherapy benefit. Compared with patients with only 1 high variable or no high variables, patients with concomitantly high TMB and PD-L1 expression had improved PFS (HR, 0.42; 95% CI, 0.30-0.58; *P* < .001) (eFigure 24 in the Supplement) and a higher complete response or partial response rate (high TMB or high PD-L1, 62.5%; high TMB and low PD-L1, 43.9%; low TMB and high PD-L1, 27.3%; low TMB and low PD-L1, 8.0%; eFigure 25A in the Supplement) or DCB rate (high TMB or high PD-L1, 77.3%; high TMB and low PD-L1, 57.1%; low TMB and high PD-L1, 63.6%; low TMB and low PD-L1, 25.9%) (eFigure 25B in the Supplement). Among patients with NSCLC who had undergone whole-exome sequencing, the clinical use of TMB combined with PD-L1 expression had a higher value of prediction with PFS, complete response, partial response, or DCB than TMB or PD-L1 expression alone (1-year PFS: AUC, 0.829; 3-year PFS: AUC, 0.839; ORR: AUC, 0.803; DCB: AUC, 0.740) (Figure 3A and B; eFigure 22 in the Supplement). The predictability of TMB quantified by targeted next-generation sequencing (Memorial Sloan Kettering-Integrated Mutation Profiling of Actionable Cancer Targets) combined with PD-L1 expression was further validated in tumor tissues (1-year PFS: AUC, 0.826; 3-year PFS: AUC, 0.948) (Figure 3C and D). We also assessed its prognostic use in the Cancer Genome Atlas^40^ cohort (eFigure 26 in the Supplement).

**Figure 4.**
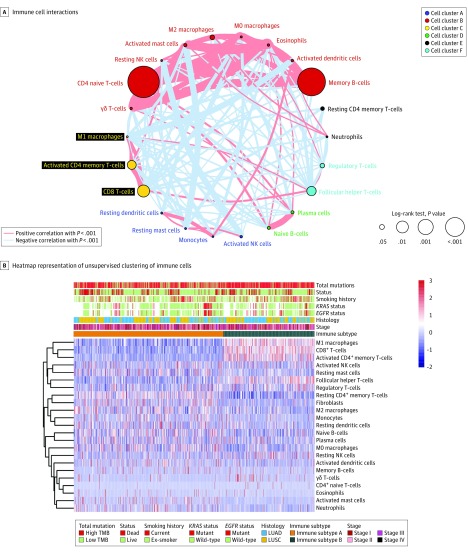
Landscape of the Immune Cells in Non–Small Cell Lung Cancer A, Cellular interaction of immune cell types. B, The heatmap representation of the unsupervised clustering of immune cells. *EGFR* indicates epidermal growth factor receptor; *KRAS*, Kirsten rat sarcoma viral oncogene homologue; LUAD, lung adenocarcinoma; LUSC, lung squamous cell carcinoma; NK, natural killer; and TMB, tumor mutation burden.

Further, a network of ICs that represented a comprehensive landscape of IC interactions was established (Figure 4A). Through unsupervised consensus matrix analyses (eFigure 27 in the Supplement), we developed an immune signature consisting of 2 immune subtypes (Figure 4B). Tumor mutation burden was significantly greater in the patients with immune subtype B compared with those with immune subtype A (eFigure 28A in the Supplement), and the immune subtype B group was associated with a higher OS (HR, 0.71; 95% CI, 0.55-0.90; *P* = .006) (eFigure 28B in the Supplement). Patients with immune subtype B had a higher proportion of high CD8^+^ T-cell tumor-infiltrating lymphocytes (TILs) compared with patients with immune subtype A (319 of 353 [90.4%] vs 187 of 588 [31.8%]) (eFigure 28C in the Supplement). Kaplan-Meier analysis found a significant difference in OS between the patients with high-CD8^+^ T-cell TILs vs those with low CD8^+^ T-cell TILs (HR, 0.67; 95% CI, 0.53-0.85; *P* < .001) (eFigure 28D in the Supplement). Random forest analysis consistently confirmed that the infiltration of CD8^+^ T-cell TILs was the most important variable in our clustering (eFigure 29 in the Supplement).

Next, we demonstrated that CD8^+^ T-cell TILs could further synergize with PD-L1 expression and TMB to have a prognostic association in the Cancer Genome Atlas^40^ cohort (3-year OS: AUC, 0.659; 5-year OS: AUC, 0.665) (eFigure 26 in the Supplement). Small correlations were identified between CD8^+^ T-cell TILs and PD-L1 expression (Spearman ρ = 0.21), between TMB and CD8^+^ T-cell TILs (Spearman ρ = 0.20), and between PD-L1 expression and TMB (Spearman ρ = 0.06) (eFigure 30 in the Supplement).

### Association of Individual Gene Alterations With Response and Resistance to ICI Therapy 

**Figure 5.**
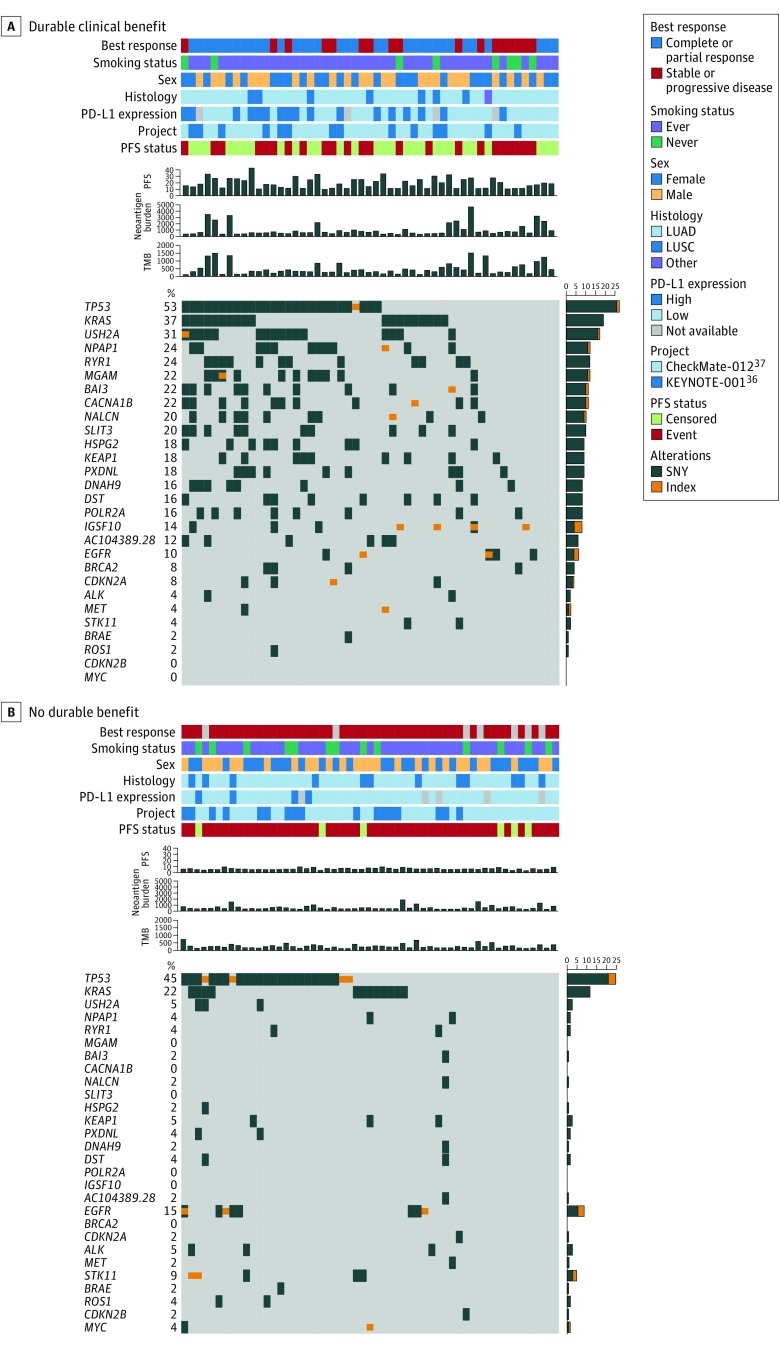
Individual Gene Alterations Associated With Response and Molecular Features in Checkpoint Blockade LUAD indicates lung adenocarcinoma; LUSC, lung squamous cell carcinoma; PD-L1, programmed cell death ligand 1; PFS, progression-free survival; SNV, single-nucleotide variants; and TMB, tumor mutation burden.

Within the KEYNOTE-001^36^ and CheckMate-012^37^ cohorts, we examined the associations of the frequencies of common NSCLC carcinogenic driver mutations with the clinical benefit derived from checkpoint blockade (Figure 5; eFigure 31A in the Supplement). Gene alterations were mostly enriched in the DCB group. Mutations in *TP53* (DCB, 27 of 51 [53%]; NDB, 25 of 55 [45%]) and *KRAS* (DCB, 19 of 51 [37%]; NDB, 12 of 55 [22%]) were common and associated with increased responsiveness but did not reach statistical significance. Additionally, we identified other potential genes associated with responsiveness, such as *USH2A* (DCB, 17 of 51 [33%]; NDB, 3 of 55 [5%]; *P* < .001), *NPAP1* (DCB, 12 of 51 [24%]; NDB, 2 of 55 [4%]; *P* < .001), *RYR1* (DCB, 12 of 51 [24%]; NDB, 2 of 55 [4%]; *P* < .001), and *MGAM* (DCB, 12 of 51 [24%]; NDB, 0 of 55; *P* < .001). However, the frequencies of mutations in *EGFR*, *ALK*, *ROS1*, *STK11*, and *BRAF* were not significantly different between the DCB group and the NDB group (*EGFR*: DCB, 6 of 51 [10%]; NDB, 9 of 55 [16%]; *ALK*: DCB, 2 of 51 [4%]; NDB, 3 of 55 [5%]; *ROS1*: DCB, 1 of 51 [2%]; NDB, 2 of 55 [4%]; *STK11*: DCB, 2 of 51 [4%]; NDB, 5 of 55 [9%]; and *BRAF*: DCB, 1 of 51 [2%]; NDB, 1 of 55 [2%]). We also compared the frequencies of altered genes in the high TMB group with those in the low TMB group and those in the high PD-L1 group with those in the low PD-L1 group. Notably, *RYR1* or *MGAM* mutations were associated with concomitantly increased DCB (*RYR1*: DCB, 12 of 51 [24%]; NDB, 2 of 55 [4%]; *P* < .001; *MGAM*: DCB, 12 of 51 [24%]; NDB, 0 of 55; *P* < .001), a higher TMB (*RYR1*: high TMB, 12 of 53 [23%]; low TMB, 2 of 53 [38%]; *P* < .001; *MGAM*: high TMB, 9 of 53 [17%]; low TMB, 0 of 53; *P* < .001), and higher PD-L1 expression (*RYR1*: high PD-L1, 8 of 30 [27%]; low PD-L1, 6 of 85 [7%]; *P* < .001; *MGAM*: high PD-L1, 6 of 30 [20%]; low PD-L1, 5 of 85 [6%]; *P* < .001) (eFigure 31B and C in the Supplement).

## Discussion

Our analysis revealed that immunotherapies, including ICIs, tumor vaccines, and cellular immunotherapy, were associated with improved OS and PFS in patients with advanced NSCLC. Our results indicated that first-line pembrolizumab with platinum-based chemotherapy was superior to other ICI regimens and therefore should be the preferred choice for patients with advanced NSCLC. Other strategies, such as first-line tumor vaccines with chemotherapy and first-line tumor vaccine maintenance therapy, also showed promising results with high-quality evidence. Additionally, increasing PD-L1 expression and TMB were associated with increasing improvements in OS and PFS in patients with NSCLC treated with ICIs. We further found improved OS and PFS associated with using a single ICI for patients in the TC3 or IC3 and TC2/3 or IC2/3 groups, whereas ICIs with chemotherapy might be more suitable for patients in the TC1 or IC1 groups. The combined predictive relevance of PD-L1 expression and TMB was found to be a promising biomarker for patient survival and response to precision immunotherapy. We also demonstrated the potential to jointly use CD8^+^ T-cell TILs with PD-L1 expression and TMB in candidate identification.

There is an increasing immunotherapeutic trend in dual immunotherapy, which has shown to be effective in treating melanoma^42^ and renal cell carcinoma^43^ in RCTs. The results of a 2017 phase 3 RCT^42^ with 945 patients with advanced melanoma showed that nivolumab with ipilimumab as a first-line treatment was associated with longer OS than nivolumab or ipilimumab alone. The combination of ICIs and tumor vaccines, such as sargramostim, with ipilimumab showed significantly longer OS and lower toxic effects than ipilimumab alone in treating melanoma.^44^ In addition, the efficacy of chimeric antigen receptor T-cells in treating NSCLC is being evaluated in a randomized phase 1/2 trial (ClinicalTrials.gov identifier: NCT03525782), although RCTs concerning the combination of chimeric antigen receptor T-cells and immunotherapy remain restricted to hematologic malignancies, to our knowledge.^45^ Overall, the evidence of the beneficial role of various dual therapies that combine ICIs with tumor vaccines or cellular immunotherapy for the treatment of NSCLC remains too preliminary, and these combinations need to be further explored and established.

Independent biomarkers, such as PD-L1 expression and TMB, are probably inadequately predictive in intratumoral immune microenvironments with heterogeneous features, and an integrated multiparameter evaluation will aid efforts to overcome within-tumor heterogeneity and identify patients who could derive the greatest therapeutic benefit.^46^ Based on the data from a 2018 pan-tumor study,^47^ the highest ORR and longest PFS were observed in patients with high PD-L1 expression and a high TMB. This finding is also supported in our study, and we further demonstrated that the joint use of PD-L1 expression and TMB represented greater predictive and prognostic relevance for immunotherapy survival and response than the PD-L1 expression or TMB alone, which merits future clinical investigation on the combined use of PD-L1 expression and TMB as a predictor.

Characterizing the immune infiltrate could facilitate evaluating the treatment effect of immunotherapy; immune-inflamed tumors, which have a higher density of CD8^+^ T-cell TILs than immune-desert tumors, can elicit a strong immune response.^48,49,50^ The IMpassion130 RCT,^51^ a phase III study that evaluated the predictive effect of ICs on atezolizumab with nab-paclitaxel therapy in patients with triple-negative breast cancer, showed that high intratumoral CD8^+^ expression was significantly associated with PD-L1 expression and improvements in OS and PFS. According to our findings, using CD8^+^ T-cell TILs, PD-L1 expression, and TMB as an integrated variable was associated with improved OS and PFS compared with using a single biomarker or a combination of 2 of these 3 biomarkers. Future development of an optimized, integrated predictive model for immunotherapy should consider the integration of multiple approaches involving biomarkers associated with the T cell–inflamed tumor microenvironment, such as PD-L1 expression, ICs, and those associated with tumor neoepitope burden.

The examination of oncogenic driver mutations is becoming a novel approach to identify appropriate patients for immunotherapy. However, previous small-scale cohort studies showed inconsistent results concerning the associations of oncogenic alterations with immunotherapeutic outcomes. A study by Dong et al^52^ demonstrated that *KRAS* or *TP53* mutations were statistically significantly associated with an increased clinical benefit of immunotherapy in NSCLC, but these findings were not confirmed in later studies.^53,54^ In our study, we used the largest data series to date, to our knowledge, and found that the differences between the DCB group and the NDB group were not statistically significant when the patients were stratified by *TP53* or *KRAS* status. Mutated genes in patients with NSCLC probably represent diverse molecular functions; thus, cooccurring patterns of mutations could describe different patient immune subsets that are associated with distinct clinical benefits,^55^ which may also explain the observed contradictory findings. A 2018 cohort study^53^ of lung adenocarcinoma showed that *KRAS* mutation was not associated with enhanced response or survival among patients with *TP53*, *STK11*, *EGFR*, or wild-type NSCLC. In agreement, our nonsignificant results for *KRAS* may be owing to most of the patients in our included cohorts exhibiting the *TP53*, *STK11*, *EGFR*, or wild-type status. Of note, we further identified novel genes, such as *RYR1* and *MGAM*, in which mutations were significantly associated with increased response and higher PD-L1 expression and TMB, indicating that these genes may contribute to IC infiltration and may be important components of the immunogenetic landscape. Future prospective sequencing tools that examine predictors for immunotherapy should be able to expand the landscape of immuno-oncological genes to more fully realize the potential for precision immunotherapy.

### Limitations

This study has several limitations. First, we could not perform further subgroup analyses and examine distinct molecular features among the patients who received tumor vaccines and cellular immunotherapy owing to limited data. Additional trials are needed to identify the candidates who would most benefit from treatment with tumor vaccines and cellular immunotherapy. Second, patient data were limited regarding dual immunotherapy vs a single agent. The beneficial role of dual immunotherapy in NSCLC remained unclear. Third, the results about the biomarkers are somewhat scattered, as there were patients who did not receive quantification of both PD-L1 and TMB. Fourth, the interpretation in the ICI cohorts was restricted to an inadequate type of data owing to mainly using exome data; therefore, an integrated analysis based on multidimensional data comprising genomics, transcriptomics, proteomics, and signaling pathways might represent a powerful explanation of our findings.

## Conclusions

In this study, overall, ICIs, tumor vaccines, and cellular immunotherapy showed promising clinical outcomes in patients with NSCLC. We recommend pembrolizumab with platinum-based chemotherapy as the most appropriate first-line ICI regimen for advanced NSCLC and suggest the combined use of PD-L1 expression and TMB to evaluate patients’ survivals and responses to precision immunotherapy. Moreover, the combination of CD8^+^ T-cell TILs, PD-L1 expression, and TMB were associated with reliable prognostic relevance. The predictive value of that combination needs to be prospectively validated in large-scale studies.

## Notes

Supplement. **eAppendix.** Methods **eTable 1.** Characteristics of the Included Patients and Randomized Clinical Trials **eTable 2.** Characteristics and Clinical Outcomes of the Individual Patients in Checkpoint Inhibitor Cohorts **eTable 3.** Summary of the Pooled Estimates and Grading of Recommendations, Assessment, Development, and Evaluation Evidence of Progression-Free Survival **eTable 4.** Summary of the Pooled Estimates and Grading of Recommendations, Assessment, Development, and Evaluation Evidence of Overall Survival **eTable 5.** Summary of the Pooled Estimates and Grading of Recommendations, Assessment, Development, and Evaluation Evidence of Objective Response Rate **eTable 6.** Summary of the Estimates and Grading of Recommendations, Assessment, Development, and Evaluation Evidence in the Subgroup Analysis of Clinical Outcomes **eTable 7.** Summary of the Estimates Stratified by Programmed Cell Death Ligand 1 Expression and Treatment Strategy **eFigure 1.** PRISMA Flow Diagram for the Meta-analysis **eFigure 2.** Risk of Bias Summary of the Randomized Clinical Trials Included in the Meta-analysis **eFigure 3.** Risk of Bias Graph for the Randomized Clinical Trials Included in the Meta-analysis **eFigure 4.** Analysis of Publication Bias in the Meta-analyses of Immunotherapy Versus Conventional Therapy **eFigure 5.** Pooled Analysis of the Ratio of the Median Overall Survival With Immunotherapy vs Conventional Therapy **eFigure 6.** Pooled Hazard Ratios for Progression-Free Survival With Immunotherapy vs Conventional Therapy **eFigure 7.** Pooled Analysis of the Ratio of the Median Progression-Free Survival With Immunotherapy vs Conventional Therapy **eFigure 8.** Network Diagram of Studies Comparing Clinical Outcomes of Different Immune Checkpoint Inhibitors Strategies for Advanced Non–Small Cell Lung Cancer **eFigure 9.** Network Meta-analysis of Immune Checkpoint Inhibitors in Terms of Progression-Free Survival **eFigure 10.** Network Meta-analysis of Immune Checkpoint Inhibitors in Terms of Overall Survival **eFigure 11.** Network Meta-analysis of Immune Checkpoint Inhibitors as a First-line Therapy in Terms of Progression-Free Survival **eFigure 12.** Network Meta-analysis of Immune Checkpoint Inhibitors as a First-line Therapy in Terms of Overall Survival **eFigure 13.** Network Meta-analysis of Immune Checkpoint Inhibitors in Terms of Progression-Free Survival in Previously Treated Patients **eFigure 14.** Network Meta-analysis of Immune Checkpoint Inhibitors in Terms of Overall Survival in Previously Treated Patients **eFigure 15.** Pooled Analysis of the Objective Response Rate With Immunotherapy vs Conventional Therapy **eFigure 16.** Trial Sequential Analyses of Trials Comparing Immunotherapy With Conventional Therapy **eFigure 17.** Subgroup Analyses of Progression-Free Survival in Patients Receiving Immune Checkpoint Inhibitor Therapy **eFigure 18.** Subgroup Analyses of Overall Survival in Patients Receiving Immune Checkpoint Inhibitor Therapy **eFigure 19.** Response and Clinical Benefit to Checkpoint Inhibitor Relative to Molecular Features in Cohort 1 **eFigure 20.** Progression-Free Survival Analysis Stratified by Neoantigen Burden in Cohort 1 **eFigure 21.** Overall Survival Analysis Stratified by Molecular Features in the Cancer Genome Atlas Cohort **eFigure 22.** Receiver Operating Characteristic Curves Correlating Molecular Features With Clinical Outcomes in Cohort 1 **eFigure 23.** Scatterplots of Molecular Features in Cohort 1 **eFigure 24.** Progression-Free Survival Analysis Stratified by Programmed Cell Death Ligand 1 Expression and Tumor Mutation Burden in Cohort 1 **eFigure 25.** Response and Clinical Benefit to Checkpoint Inhibitor Stratified by Programmed Cell Death Ligand 1 and Tumor Mutation Burden **eFigure 26.** Receiver Operating Characteristic Curves Correlating Molecular Features With Survival in the Cancer Genome Atlas Cohort **eFigure 27.** Unsupervised Consensus Clustering of Immune Subtypes in the Cancer Genome Atlas Cohort **eFigure 28.** Molecular Features and Survival Stratified by Immune Subtype in the Cancer Genome Atlas Cohort **eFigure 29.** Identification of the Most Important Immune Feature Using Random Forest Method **eFigure 30.** Scatterplots of Molecular Features in the Cancer Genome Atlas Cohort **eFigure 31.** Individual Gene Alterations Associated With Checkpoint Blockade Benefits and Molecular Features **eReferences.**
