# Supplementary material for: Association of Survival and Immune-Related Biomarkers With Immunotherapy in Patients With Non–Small Cell Lung Cancer: A Meta-analysis and Individual Patient–Level Analysis
Source: JAMA Netw Open. 2019 Jul 10;2(7):e196879. doi: 10.1001/jamanetworkopen.2019.6879 (PMC6625073; doi:10.1001/jamanetworkopen.2019.6879)
Supplement: Supplement. — eAppendix. Methods eTable 1. Characteristics of the Included Patients and Randomized Clinical Trials eTable 2. Characteristics and Clinical Outcomes of the Individual Patients in Checkpoint Inhibitor Cohorts eTable 3. Summary of the Pooled Estimates and Grading of Recommendations, Assessment, Development, and Evaluation Evidence of Progression-Free Survival eTable 4. Summary of the Pooled Estimates and Grading of Recommendations, Assessment, Development, and Evaluation Evidence of Overall Survival eTable 5. Summary of the Pooled Estimates and Grading of Recommendations, Assessment, Development, and Evaluation Evidence of Objective Response Rate eTable 6. Summary of the Estimates and Grading of Recommendations, Assessment, Development, and Evaluation Evidence in the Subgroup Analysis of Clinical Outcomes eTable 7. Summary of the Estimates Stratified by Programmed Cell Death Ligand 1 Expression and Treatment Strategy eFigure 1. PRISMA Flow Diagram for the Meta-analysis eFigure 2. Risk of Bias Summary of the Randomized Clinical Trials Included in the Meta-analysis eFigure 3. Risk of Bias Graph for the Randomized Clinical Trials Included in the Meta-analysis eFigure 4. Analysis of Publication Bias in the Meta-analyses of Immunotherapy Versus Conventional Therapy eFigure 5. Pooled Analysis of the Ratio of the Median Overall Survival With Immunotherapy vs Conventional Therapy eFigure 6. Pooled Hazard Ratios for Progression-Free Survival With Immunotherapy vs Conventional Therapy eFigure 7. Pooled Analysis of the Ratio of the Median Progression-Free Survival With Immunotherapy vs Conventional Therapy eFigure 8. Network Diagram of Studies Comparing Clinical Outcomes of Different Immune Checkpoint Inhibitors Strategies for Advanced Non–Small Cell Lung Cancer eFigure 9. Network Meta-analysis of Immune Checkpoint Inhibitors in Terms of Progression-Free Survival eFigure 10. Network Meta-analysis of Immune Checkpoint Inhibitors in Terms of Overall Survival eFigure 11. Network Me [file jamanetwopen-2-e196879-s001.pdf]

Supplementary Online Content

Yu Y, Zeng D, Ou Q, et al. Association of survival and immune-related biomarkers with immunotherapy in patients with non–small cell lung cancer: a meta-analysis and individual patient-level analysis. *JAMA Netw Open*. 2019;2(7):e196879. doi:10.1001/jamanetworkopen.2019.6879

eAppendix. Methods
eTable 1. Characteristics of the Included Patients and Randomized Clinical Trials
eTable 2. Characteristics and Clinical Outcomes of the Individual Patients in Checkpoint Inhibitor Cohorts
eTable 3. Summary of the Pooled Estimates and Grading of Recommendations, Assessment, Development, and Evaluation Evidence of Progression-Free Survival
eTable 4. Summary of the Pooled Estimates and Grading of Recommendations, Assessment, Development, and Evaluation Evidence of Overall Survival
eTable 5. Summary of the Pooled Estimates and Grading of Recommendations, Assessment, Development, and Evaluation Evidence of Objective Response Rate
eTable 6. Summary of the Estimates and Grading of Recommendations, Assessment, Development, and Evaluation Evidence in the Subgroup Analysis of Clinical Outcomes
eTable 7. Summary of the Estimates Stratified by Programmed Cell Death Ligand 1 Expression and Treatment Strategy
eFigure 1. PRISMA Flow Diagram for the Meta-analysis
eFigure 2. Risk of Bias Summary of the Randomized Trials Included in the Meta-analysis
eFigure 3. Risk of Bias Graph for the Randomized Trials Included in the Meta-analysis
eFigure 4. Analysis of Publication Bias in the Meta-analyses of Immunotherapy vs Conventional Therapy
eFigure 5. Pooled Analysis of the Ratio of the Median Overall Survival With Immunotherapy vs Conventional Therapy
eFigure 6. Pooled Hazard Ratios for Progression-Free Survival With Immunotherapy vs Conventional Therapy
eFigure 7. Pooled Analysis of the Ratio of the Median Progression-Free Survival With Immunotherapy vs Conventional Therapy
eFigure 8. Network Diagram of Studies Comparing Clinical Outcomes of Different Immune Checkpoint Inhibitors Strategies for Advanced Non–Small Cell Lung Cancer
eFigure 9. Network Meta-analysis of Immune Checkpoint Inhibitors in Terms of Progression-Free Survival
eFigure 10. Network Meta-analysis of Immune Checkpoint Inhibitors in Terms of Overall Survival
eFigure 11. Network Meta-analysis of Immune Checkpoint Inhibitors as a First-line Therapy in Terms of Progression-Free Survival

© 2019 Yu Y et al. *JAMA Network Open.*

eFigure 12. Network Meta-analysis of Immune Checkpoint Inhibitors as a First-line Therapy in Terms of Overall Survival
eFigure 13. Network Meta-analysis of Immune Checkpoint Inhibitors in Terms of Progression-Free Survival in Previously Treated Patients
eFigure 14. Network Meta-analysis of Immune Checkpoint Inhibitors in Terms of Overall Survival in Previously Treated Patients
eFigure 15. Pooled Analysis of the Objective Response Rate With Immunotherapy vs Conventional Therapy
eFigure 16. Trial Sequential Analyses of Trials Comparing Immunotherapy With Conventional Therapy
eFigure 17. Subgroup Analyses of Progression-Free Survival in Patients Receiving Immune Checkpoint Inhibitor Therapy
eFigure 18. Subgroup Analyses of Overall Survival in Patients Receiving Immune Checkpoint Inhibitor Therapy
eFigure 19. Response and Clinical Benefit to Checkpoint Inhibitor Relative to Molecular Features in Cohort 1
eFigure 20. Progression-Free Survival Analysis Stratified by Neoantigen Burden in Cohort 1
eFigure 21. Overall Survival Analysis Stratified by Molecular Features in the Cancer Genome Atlas Cohort
eFigure 22. Receiver Operating Characteristic Curves Correlating Molecular Features With Clinical Outcomes in Cohort 1
eFigure 23. Scatterplots of Molecular Features in Cohort 1
eFigure 24. Progression-Free Survival Analysis Stratified by Programmed Cell Death Ligand 1 Expression and Tumor Mutation Burden in Cohort 1
eFigure 25. Response and Clinical Benefit to Checkpoint Inhibitor Stratified by Programmed Cell Death Ligand 1 and Tumor Mutation Burden
eFigure 26. Receiver Operating Characteristic Curves Correlating Molecular Features With Survival in the Cancer Genome Atlas Cohort
eFigure 27. Unsupervised Consensus Clustering of Immune Subtypes in the Cancer Genome Atlas Cohort
eFigure 28. Molecular Features and Survival Stratified by Immune Subtype in the Cancer Genome Atlas Cohort
eFigure 29. Identification of the Most Important Immune Feature Using Random Forest Method
eFigure 30. Scatterplots of Molecular Features in the Cancer Genome Atlas Cohort
eFigure 31. Individual Gene Alterations Associated With Checkpoint Blockade Benefits and Molecular Features
eReferences.

This supplementary material has been provided by the authors to give readers additional information about their work.

eAppendix. Methods

Study design

Meta-analysis

Search strategy and selection criteria. The meta-analysis was reported in accordance with PRISMA reporting guideline. A systematic literature search of the PubMed, EMBASE, and Cochrane Central Register of Controlled Trials databases was performed to identify relevant randomized clinical trials (RCTs) published prior to June 2018. The search was performed with the search keywords and MeSH terms pertinent to the intervention of interest, such as “tumor vaccine”, “cellular immunotherapy”, “immune checkpoint inhibitor”, “cytotoxic T-lymphocyte-associated protein 4”, “programmed death receptor 1”, “programmed death-ligand 1”, “ipilimumab”, “tremelimumab”, “atezolizumab”, “durvalumab”, “nivolumab”, “pembrolizumab”, “non-small cell lung carcinoma” and “randomized clinical trial”. Furthermore, we also manually searched and checked references of systematic reviews, meta-analyses and conference proceedings of the American Society of Clinical Oncology, the European Society for Medical Oncology, the American Association for Cancer Research, and the World Conference on Lung Cancer. The latest update was in July 2018. The following inclusion criteria were applied: (1) randomized trials comparing immune checkpoint inhibitors, tumor vaccines, or cellular immunotherapy with conventional therapy for patients with advanced or metastatic non-small cell lung carcinoma (NSCLC); (2) trials with reported available data that measured overall survival (OS), progression-free survival (PFS), or objective response rate (ORR); and (3) Trials published in English. The PRISMA flow diagram was shown in eFigure 1 in the supplement.

Study selection, data extraction and quality assessment. Two reviewers (Y.F.Y and S.B.L) independently and in duplicate screened titles and abstracts to identify relevant studies. Studies that appeared to meet the inclusion criteria were further checked by full-text review. Data extraction was also conducted independently by two reviewers (Y.F.Y and S.B.L). Data collected included the first author (or registration number), study design, sample size, treatment group allocated, line of therapy, details about immunotherapy regimens, and patients and tumor characteristics. Hazard ratios (HRs) of OS and PFS with their 95% corresponding confidence intervals (CIs) and the numbers of responders were also extracted. If an inconsistency arose, a consensus was reached by discussion among all investigators. Two reviewers (Y.F.Y and S.B.L) rigorously and independently assessed the risk of bias by using an approach based on the Cochrane Collaboration Handbook[1] with the following seven domains: (1) random sequence generation; (2) allocation concealment; (3) blinding of the participants and personnel; (4) blinding of the outcome assessment; (5) incomplete outcome data; (6) selective outcome reporting; and (7) other biases. Each item was categorized as low, unclear, or high risk of bias by using Review Manager 5.3 software (Cochrane Community). The result of the methodological quality of the randomized trials was shown in eFigure 2 and eFigure 3 in the supplement.

Individual patient-level analysis

© 2019 Yu Y et al. *JAMA Network Open.*

Checkpoint inhibitor cohorts. Patients were also eligible if they had durable clinical benefit (DCB; complete response [CR]/partial response [PR] or stable disease [SD] that lasted > 6 months) or no durable benefit (NDB; progressive disease [PD] or SD that lasted < 6 months), which was assessed with Response Evaluation Criteria in Solid Tumors (RECIST) version 1.1.[2]

The Cancer Genome Atlas (TCGA) cohort. Level 3 data (FPKM normalized) for genes from 1,008 lung adenocarcinoma and lung squamous cell carcinoma samples processed on 2017-09-14 were downloaded from the UCSC Xena browser (http://xena.ucsc.edu/) GDC hub. The expression profile (FPKM normalized) was transformed to Transcripts Per Million Kilobases (TPM), converting the FPKM data into values that were more comparable between samples.

Statistical analysis

Meta-analysis

HRs and 95% CIs were pooled to estimate the survival increases in PFS and OS. Dichotomous data, such as ORR data, were analyzed using the risk ratio (RR). The Mantel-Haenszel random-effects model was utilized. Two-sided *P* values less than 0.05 were regarded as statistically significant. I^2 was used to assess the heterogeneity between trials; an I^2 value exceeding 50% indicated the existence of substantial heterogeneity. In addition, the OS and PFS of immunotherapy were compared with those of conventional therapy in key subgroups defined by age, ECOG score, epithelial growth factor receptor (EGFR) status, kirsten rat sarcoma viral oncogene homolog (KRAS) status, histology, programmed death-ligand 1(PD-L1) expression, sex, smoking status and tumor mutation burden (TMB). The differences in treatment impact between subgroups were measured by *P* value for interaction. We evaluated median survival outcomes by assessing the ratio of the median months of survival (Median month ratio = Median $\text{month}_{\textit{immunotherapy group}}$ / Median $\text{month}_{\textit{conventional therapy group}}$). In addition, we conducted network meta-analyses to compare the OS and PFS of different immune checkpoint inhibitor strategies using the random effects Bayesian model.[3] For example, the relative treatment effect of treatment A versus treatment B can be indirectly obtained from the comparisons of treatment A with treatment C and treatment B with treatment C.

The role of trial sequential analysis (TSA) in a meta-analysis is analogous to that of interim analysis in a single trial, with boundaries to decide whether current trials have enough power to draw a reliable conclusion.[4] TSA adjusts the thresholds for the Z values of benefits, harm and futility to reduce type I (false positive) and type II (false negative) errors due to sparse data and repetitive significance testing and could be used to calculate required information size (RIS).[5-7] Sufficient evidence for the anticipated intervention effect is established and no further trials are needed when the cumulative Z-curve crosses the TSA monitoring boundary, while experimental intervention can be considered minimally important to patients if the Z-curve enters the futility boundary. When the Z-curve crosses neither the TSA monitoring boundary for benefit nor the futility boundary for harm, the meta-analysis is inconclusive. A random effects model with an O'Brien-Fleming α-spending function and a two-side boundary was utilized to calculate the TSA monitoring boundary, futility boundary and RIS. A relative risk reduction of 20%, a type I error rate of 5% and a type II error rate of 20% (power = 80%) were set. We adjusted heterogeneity based on model

© 2019 Yu Y et al. *JAMA Network Open.*

variance. TSA was performed by using TSA version 0.9 beta software (Copenhagen Trial Unit 2011).

Potential publication bias was visually evaluated using funnel plots and the Copas selection model[8] and was quantified using Begg's and Egger's regression tests.[9,10] A *P* value > 0.05 was considered the criterion for a negligible probability of publication bias. The results of the publication bias analyses were shown in eFigure 4 in the supplement.

The grading of recommendations assessment, development, and evaluation methodology was used to categorize the quality of the evidence as high, moderate, low, or very low. RCTs were initially considered high-quality evidence but could be rated lower because of a risk of bias, imprecision, inconsistency, indirectness, and publication bias.[11,12]

Individual patient-level analysis

Aggregated PFS and OS were computed using the Kaplan-Meier estimates method and compared with the log-rank test. HRs and 95% CIs were calculated by using the Cox regression model. Treatment effect between two groups were also calculated using the difference in restricted mean survival time.[13] Categorical variables were compared with χ^2 or Fisher's exact test, and continuous variables were compared with Wilcoxon rank sum tests for two-group comparisons or the Kruskal-Wallis exact test for multiple comparisons. PD-L1, the TMB and the neoantigen burden (NAB) were categorized into high and low value groups with the optimal cutoff values defined by the R package ggsurvimier. TMB was defined as number of nonsynonymous single nucleotide variants (SNVs) or indels variants. Spearman rank correlation coefficients were used to estimate the correlations. Receiver operating characteristic curves were generated to assess the sensitivity and specificity of continuous variables with the area under the curve. All statistical analyses were performed with R (version 3.4.1, The R Foundation), and a *P* value less than 0.05 was considered statistically significant.

An oncoprint plot was established to compare the differences in the frequencies of altered genes in the DCB and NDB groups using the R package ComplexHeatmap. The Boruta algorithm (a wrapper built around the Random Forest classification algorithm) was used to select DCB-relevant nonsynonymous alterations (SNVs or indels) with 100 iterations.[14] MutSigCV analysis was applied to identify genes that mutated at statistically significant levels,[15] and the frequencies of the identified genes were compared between the DCB group and the NDB group, between the high TMB group and the low TMB group, and between the high PD-L1 expression group and the low PD-L1 expression group using odds ratios and Fisher's exact test.
To quantify the proportions of immune cells in the NSCLC samples, we used the CIBERSORT algorithm[16] and the LM22 gene signature, which allows for highly sensitive and specific discrimination of 22 human immune cell phenotypes including B cells, T cells, natural killer cells, macrophages, dendritic cells, and myeloid subsets. CIBERSORT is a deconvolution algorithm that uses a set of reference gene expression values (a signature with 547 genes) that is considered a minimal representation for each cell type. Based on those values, CIBERSORT infers cell type proportions in data from bulk tumor samples with mixed cell types using support vector regression. gene expression profiles were prepared using standard annotation files, the data were uploaded to

© 2019 Yu Y et al. *JAMA Network Open.*

the CIBERSORT web portal (http://cibersort.stanford.edu/), and the algorithm was run using the LM22 signature at 1,000 permutations. Tumors with qualitatively different tumor microenvironment cell infiltration patterns were grouped using a hierarchical agglomerative clustering (based on Euclidean distance and Ward's linkage) method. Unsupervised clustering methods (K-means)[17] were used to analyze the dataset, identify tumor microenvironment patterns and classify patients for further analysis. While applying the consensus clustering algorithm, we varied the cluster number from 2 to 6 to determine the optimal number of clusters, which was associated with the highest stability and the lowest ambiguity. Random forest method was utilized to rank the importance of features (multiple immunologic cells), and features with high score of mean decrease accuracy or mean decrease gini were considered more important than those with low score. This procedure using the ConsensusClusterPlus R package[18] was repeated 1,000 times to ensure the stability of the classification.

© 2019 Yu Y et al. *JAMA Network Open.*

eTable 1. Characteristics of the Included Patients and Randomized Clinical Trials

Trial (Year)	Study Design	Tumor Stage	No. of Patients (ECOG PS < 2, %)	Treatment Type	Intervention Drug[a]	Control Drug[a]	Line of Therapy	STEC	Target of Therapy	EGFR or ALK Mutation Status
Butts et al,[19] (2005)	RCT, phase Iib	IIIB-IV	171 (95.3)	TV	Tecemotide + Cyclophosphamide	BSA	First-line MT	RECIST/WHO	MUC1	NP
Quoix et al,[20] (2011)	RCT, phase Iib	IIIB-IV	148 (99.3)	TV	TG4010 + Platinum-based chemotherapy	Platinum-based chemotherapy	First-line	WHO	MUC1	NP
Alfonso et al,[21] (2014)	RCT	IIIB-IV	176 (96.2)	TV	Racotumomab-alum	Placebo	First-line MT	RECIST	NeuGcGM3 ganglioside	NP
START,[22] (2014)	RCT, phase III	III	1,239 (99.4)	TV	Tecemotide + Cyclophosphamide	Placebo + Saline	First-line MT	RECIST	MUC1	NP
Braun et al,[23] (2015)	RCT, phase II	IV	92 (96.7)	TV	Imprime PGG + Carboplatin/ Paclitaxel/ Bevacizumab	Carboplatin/ Paclitaxel/ Bevacizumab	First-line	RECIST	CR3	NP
Giaccone et al,[24] (2015)	RCT, phase	III-IV	532 (95.3)	TV	Belagenpumatucel-L	Placebo	First-line MT	RECIST	TGF-b2-antisense	NP

© 2019 Yu Y et al. *JAMA Network Open.*

	III									
TIME,[25] (2015)	RCT, phase IIb/III	IV	222 (99.5)	TV	TG4010 + Platinum-based chemotherapy	Placebo + Platinum-based chemotherapy	First-line	RECIST	MUC1	Without EGFR mutation
Rodriguez et al,[26] (2016)	RCT, Phase III	IIIB-IV	405 (90.9)	TV	CIMAvax-EGF	BSA	First-line MT	RECIST	EGF	NP
Takayama et al,[27] (2016)	RCT, phase II	IIIB-IV/ recurrent	50 (100)	TV	Personalized peptide vaccination + Docetaxel	Docetaxel + Placebo	Second-line	RECIST/ WHO	EGFR	Without EGFR mutation
Katakami et al,[28] (2017)	RCT, phase I/II	III	172 (100)	TV	Tecemotide + Cyclophosphamide	Placebo + Saline	First-line MT	RECIST	MUC1	EGFR Mutant (n = 16, 9.3%)
Thomas et al,[29] (2017)	RCT, phase II	IIIB-IV	90 (95.5)	TV	BTH1677 + Cetuximab + Platinum-based chemotherapy	Cetuximab + Platinum-based chemotherapy	First-line	RECIST	CR3	NP
Wu et al,[30] (2008)	RCT	IIIA- IV	59 (NP)	CIM	CIK cell + Platinum-based chemotherap	Platinum-based chemother	First-line	RECIST/ WHO	Cytokine	NP

© 2019 Yu Y et al. *JAMA Network Open.*

					y	apy				
Li et al,[31] (2012)	RCT, phase II	IIIB-IV	74 (NP)	CIM	CIK cell + Cisplatin + Gemcitabine/ Paclitaxel/Na velbine	Cisplatin + Gemcitabi ne/ Paclitaxel/ Navelbine	First-line	RECIST	Cytokine	NP
Lynch et al,[32,c] (2012)	RCT, phase II	IIIB-IV	136 (100)	ICI	Ipilimumab+ Platinum-bas ed chemotherap y (Concurrent regimen)	Platinum-based chemother apy	First-line	WHO/ icRC	CTLA-4	NP
Lynch et al,[32,c] (2012)	RCT, phase II	IIIB-IV	134 (100)	ICI	Ipilimumab + Platinum-bas ed chemotherap y (Phased regimen)	Platinum-based chemother apy	First-line	WHO/ icRC	CTLA-4	NP
CheckMate-017,[33,34] (2015)	RCT, phase III	IIIB-IV	272 (99.2)	ICI	Nivolumab	Docetaxel	Second-line	RECIST	PD-1	NP

© 2019 Yu Y et al. *JAMA Network Open.*

CheckMate-057,[33] (2015)	RCT, phase III	III-IV	582 (NP)	ICI	Nivolumab	Docetaxel	Second-line	RECIST	PD-1	NP
KEYNOTE-010,[35] (2016)	RCT, phase II/III	III-IV	1,034 (99.3)	ICI	Pembrolizumab	Docetaxel	Second-line	RECIST	PD-1	EGFR Mutant (n = 86, 8.3%) ALK Mutant (n = 28, 8%)
KEYNOTE-024,[36] (2016)	RCT, phase III	IV	305 (99.7)	ICI	Pembrolizumab	Platinum-based chemotherapy	First-line	RECIST	PD-1	Without EGFR or ALK mutation
OAK,[37] (2016)	RCT, phase III	IIIB-IV	850 (100)	ICI	Atezolizumab	Docetaxel	Second/Third-line	RECIST	PD-L1	EGFR Mutant (n = 85, 10%) ALK Mutant (n = 2, 0.2%)
POPLAR,[38] (2016)	RCT, phase II	III-IV	287 (98.9)	ICI	Atezolizumab	Docetaxel	Second/Third-line	RECIST	PD-L1	EGFR Mutant (n = 11, 13%)

© 2019 Yu Y et al. *JAMA Network Open.*

										ALK Mutant (n = 3, 5%)
CheckMate-026,[39] (2017)	RCT, phase III	IV	541 (98.9)	ICI	Pembrolizumab	Platinum-based chemotherapy	First-line	RECIST	PD-1	Without EGFR or ALK mutation
Govindan et al,[40] (2017)	RCT, phase III	IV/ recurrent	749 (99.4)	ICI	Ipilimumab + Platinum-based chemotherapy	Platinum-based chemotherapy	First-line	WHO	CTLA-4	NP
PACIFIC,[41] (2017)	RCT, phase III	III	713 (NP)	ICI	Durvalumab	Placebo	First-line MT	RECIST	PD-L1	EGFR Mutant (n = 43, 6%)
CheckMate-078,[42] (2018)	RCT, phase III	IIIB/ IV	272 (87[b])	ICI	Nivolumab	Docetaxel	Second-line	RECIST	PD-1	Without EGFR or ALK mutation
CheckMate-227,[43,d] (2018)	RCT, phase III	IV/ recurrent	139 (100)	ICI	Nivolumab + Ipilimumab	Platinum-based chemotherapy	First-line	RECIST	PD-1 and CTLA-4	Without EGFR or ALK mutation
CheckMate-2	RCT,	IV/	71 (93)	ICI	Nivolumab	Platinum-	First-line	RECIST	PD-1	Without

© 2019 Yu Y et al. *JAMA Network Open.*

27,[43,d] (2018)	phase III	recurrent				based chemotherapy				EGFR or ALK mutation
CheckMate-227,[43,d] (2018)	RCT, phase III	IV/ recurrent	101 (93)	ICI	Nivolumab + Ipilimumab	Nivolumab	First-line	RECIST	PD-1 and CTLA-4	Without EGFR or ALK mutation
Impower131,[44] (2018)	RCT, phase III	IV	683 (NP)	ICI	Atezolizumab + Platinum-based chemotherapy	Platinum-based chemotherapy	First-line	RECIST	PD-L1	NP
Impower150,[45] (2018)	RCT, phase III	IV/ recurrent	800 (99.3)	ICI	Atezolizumab + Bevacizumab + Platinum-based chemotherapy	Bevacizumab + Platinum-based chemotherapy	First-line	RECIST	PD-L1	EGFR Mutant (n = 80, 10%) ALK Mutant (n = 34, 4.3%)
KEYNOTE-021 cohort G,[46] (2018)	RCT, phase II	IIIB-IV	123 (99.5)	ICI	Pembrolizumab + Platinum-based chemotherapy	Platinum-based chemotherapy	First-line	RECIST	PD-1	Without EGFR or ALK mutation

© 2019 Yu Y et al. *JAMA Network Open.*

KEYNOTE-042,[47] (2018)	RCT, phase III	III/ IV	1,274 (NP)	ICI	Pembrolizumab	Platinum-based chemotherapy	First-line	RECIST	PD-1	NP
KEYNOTE-407,[48] (2018)	RCT, phase III	III/ IV	559 (NP)	ICI	Pembrolizumab + Platinum-based chemotherapy	Placebo + Platinum-based chemotherapy	First-line	RECIST	PD-1	NP
KEYNOTE-189,[49] (2018)	RCT, phase III	IIIB/ IV	408 (99.5)	ICI	Pembrolizumab + Platinum-based chemotherapy	Placebo + Platinum-based chemotherapy	First-line	RECIST	PD-1	Without EGFR or ALK mutation

Abbreviations: RCT, randomized clinical trial; ECOG PS, Eastern Cooperative Oncology Group Performance Status; NP, not provided; TV, Tumor vaccine; CIM, Cellular immunotherapy; ICI, immune checkpoint inhibitor; CT, chemotherapy; MT, maintenance therapy; STEC, solid tumor evaluation criteria; RECIST, The Response Evaluation Criteria in Solid Tumors; WHO, World Health Organization; icRC, immune-related response criteria; BSA, best supportive care; CR3, complement receptor 3; MUC1, mucin 1; PD-1, programmed death-1; PD-L1, programmed death-ligand 1; CTLA-4, cytotoxic T-cell lymphocyte antigen-4; CIK, cytokine-induced killer; EGF, epithelial growth factor; EGFR, epithelial growth factor receptor; TGF, transforming growth factor; ALK, anaplastic lymphoma kinase.

[a] Platinum-based chemotherapy, which consisted of carboplatin plus pemetrexed, cisplatin plus pemetrexed, carboplatin plus gemcitabine, cisplatin plus gemcitabine, carboplatin plus paclitaxel or carboplatin plus Nab-paclitaxel.

[b] 87% of the enrolled patients had an ECOG PS score of 1.

[c] This study was divided into two substudies because two different immunotherapy strategy groups were compared with the control group.

© 2019 Yu Y et al. *JAMA Network Open.*

[d] This study was divided into three substudies because three different comparisons were performed by the investigators.

© 2019 Yu Y et al. *JAMA Network Open.*

eTable 2. Characteristics and Clinical Outcomes of the Individual Patients in Checkpoint Inhibitor Cohorts

Characteristics/Clinical Outcomes		Cohort 1 No. (%)	Cohort 2 No. (%)	OAK trial No. (%)
No. of patients		349	56	420
Follow up time, median years (IQR)		3.77 (1.83-8.32)	8.29 (3.91-14.59)	12.62 (5.09-20.17)
Age, median years (IQR)		65 (57-72)	61 (56.5-67)	63 (57-70)
Sex	Male	178 (51)	24 (42.9)	257 (61)
	Female	171 (49)	32 (57.1)	163 (39)
Histology	Adenocarcinoma	274 (79)	47 (83.9)	311 (74)
	Squamous	54 (15)	7 (12.5)	109 (26)
	Other	21 (6)	2 (3.6)	0 (0)
Smoking status	Ever	281 (81)	43 (76.8)	336 (80)
	Never	68 (19)	13 (23.2)	84 (20)
Stage	IIIB	9 (3)	NP	NP
	IV	340 (97)	NP	NP
Line of therapy	First-line	51 (15)	NP	0 (0)
	Second-line	127 (36)	NP	316 (75)
	Third or more	62 (18)	NP	104 (25)
Treatment	Monotherapy	240 (69)	56 (100)	420 (100)
	Combination therapy	109 (31)	0 (0)	0 (0)
Best overall response	CR/PR	85 (24)	17 (30.4)	57 (14)
	SD	119 (34)	21 (37.5)	147 (38)
	PD	145 (42)	18 (32.1)	186 (48)
Clinical benefit	DCB	120 (34)	NP	NP
	NDB	213 (61)	NP	NP
	NR	16 (5)	NP	NP
PD-L1 expression	≥ 50%	41 (12)	NP	72 (17)
	1-49%	72 (21)	NP	168 (40)
	< 1%	73 (21)	NP	180 (43)
	NA	163 (47)	NP	0 (0)

© 2019 Yu Y et al. *JAMA Network Open.*

Characteristics/Clinical Outcomes		Cohort 1 No. (%)	Cohort 2 No. (%)	OAK trial No. (%)
Tumor mutation burden (cutoff: 175)	High	82 (23)	26 (46.4)	NP
	Low	86 (25)	30 (53.6)	NP
	NA	181 (52)	0 (0)	NP
Candidate neoantigen burden (cutoff: 517)	High	26 (7)	NP	NP
	Low	83 (24)	NP	NP
	NA	240 (69)	NP	NP

Abbreviations: IQR, interquartile range; NP, not provided.

eTable 3. Summary of the Pooled Estimates and Grading of Recommendations, Assessment, Development, and Evaluation Evidence of Progression-Free Survival

**Subgroup**	**Progression-Free Survival**					
	**No. of Trials**	**HR**	**95% CI**	***P* Value**	***I*², %**	**GRADE**
**Immune checkpoint inhibitor**						
**All trials**	22	0.76	0.71 - 0.82	<.0001	82.0	⊕⊕⊕O Moderate[a]
**First-line ICI vs CT**	5	0.88	0.69 - 1.11	.2812	84.8	⊕⊕OO Low[a,c]
Nivolumab[c]	2	1.09	0.89 - 1.34	.409	0	⊕⊕OO Low[b,c]
Pembrolizumab	2	0.74	0.35 - 1.56	.430	95.1	⊕OOO Very low[a,b,c]
Nivolumab + ipilimumab	1	0.83	0.72 - 0.96	.011	NA	⊕⊕⊕O Moderate[b]
**First-line ICI+ICI vs IC** (Nivolumab + ipilimumab vs Nivolumab)	1	0.75	0.53 - 1.07	.108	NA	⊕⊕OO Low[b,c]
**First-line ICI+CT vs CT**	8	0.68	0.58 - 0.80	< .0001	69.7	⊕⊕⊕O Moderate[a]
Ipilimumab	3	0.85	0.74 - 0.96	.014	0	⊕⊕⊕⊕ High
Pembrolizumab	3	0.54	0.47 - 0.62	< .001	0	⊕⊕⊕⊕ High
Atezolizumab	1	0.71	0.60 - 0.85	< .001	NA	⊕⊕⊕O Moderate[b]

© 2019 Yu Y et al. *JAMA Network Open.*

Nivolumab	1	0.74	0.58 - 0.94	.015	NA	⊕⊕⊕O Moderate[b]
**First-line ICI + anti-VEGFR + CT vs anti-VEGFR + CT** (Atezolizumab + bevacizumab)	1	0.61	0.52 - 0.72	< .001	NA	⊕⊕⊕O Moderate[b]
**First-line MT ICI vs CT** (Durvalumab)	1	0.52	0.42 - 0.65	< .001	NA	⊕⊕⊕O Moderate[b]
**Second/third-line ICI vs CT**	6	0.85	0.77 - 0.94	.0016	40.1	⊕⊕⊕⊕ High
Atezolizumab	2	0.95	0.83 - 1.08	0.445	0	⊕⊕OO Low[b,c]
Nivolumab	3	0.77	0.64 - 0.93	.006	54.6	⊕⊕⊕O Moderate[a]
Pembrolizumab	1	0.85	0.73 - 0.98	.03	NA	⊕⊕⊕O Moderate[b]
**Tumor vaccine**						
**All trials**	11	0.86	0.78 - 0.94	< .0001	0	⊕⊕⊕⊕ High
**First-line TV+CT vs CT**	3	0.74	0.60 - 0.91	.005	0	⊕⊕⊕⊕ High
**First-line MT TV vs no TV**	4	0.89	0.81 - 0.99	.023	0	⊕⊕⊕⊕ High
**Second-line TV+CT vs CT**	1	0.78	0.43 - 1.42	.415	NA	⊕⊕OO Low[b,c]
**Cellular immunotherapy**						
**First-line CIM+CT vs CT**	2	0.51	0.24 - 1.10	.083	73.0	⊕OOO Very low[a,b,c]

Abbreviations: GRADE, Grading of Recommendations Assessment, Development and Evaluation; vs, versus; ICI, immune checkpoint inhibitor; TV, tumor vaccine; CIM, cellular immunotherapy; CT, chemotherapy; MT, maintenance therapy; HR, hazard ratio; CI, confidence interval; VEGFR, vascular endothelial growth factor receptor; NA, not available.

[a] Downgraded (-1) for inconsistency: Substantial heterogeneity (I^2 > 50%) was found among the trials.

[b] Downgraded (-1) for imprecision: Fewer than three trials were included in this subgroup.

[c] Downgraded (-1) for imprecision: The 95% CIs were so wide that the result included no effect

© 2019 Yu Y et al. *JAMA Network Open.*

and failed to exclude important benefits or serious harmful effects.

GRADE Working Group grades of evidence:

⊕⊕⊕⊕ High quality: Further research is very unlikely to change our confidence in the estimated effect.

⊕⊕⊕O Moderate quality: Further research is likely to have an important impact on our confidence in the estimated effect and might change the estimate.

⊕⊕OO Low quality: Further research is very likely to have an important impact on our confidence in the estimated effect and might change the estimate.

⊕OOO Very low quality: We are very uncertain about the estimate.

eTable 4. Summary of the Pooled Estimates and Grading of Recommendations, Assessment, Development, and Evaluation Evidence of Overall Survival

**Subgroup**	**Overall Survival**					
	**No. of Trials**	**HR**	**95% CI**	***P* Value**	***I*², %**	**GRADE**
**Immune checkpoint inhibitor**						
**All trials**	17	0.76	0.70 - 0.83	< .0001	60.0	⊕⊕⊕O Moderate[a]
**First-line ICI vs CT**	3	0.82	0.65 - 1.03	.094	64.2	⊕⊕OO Low[a,c]
Nivolumab[c]	1	1.02	0.80 - 1.30	.873	NA	⊕⊕OO Low[b,c]
Pembrolizumab	2	0.78	0.69 - 0.89	< .001	51.3	⊕⊕OO Low[a,b]
**First-line ICI+CT vs CT**	7	0.61	0.58 - 0.93	.0102	76.5	⊕⊕⊕O Moderate[a]
Ipilimumab	3	0.991	0.79 - 1.05	.901	0	⊕⊕⊕O Moderate[c]
Pembrolizumab	3	0.55	0.46 - 0.66	< .001	0	⊕⊕⊕⊕ High
Atezolizumab	1	0.96	0.78 - 1.18	.699	NA	⊕⊕OO Low [b,c]
**First-line ICI + anti-VEGFR + CT vs anti-VEGFR + CT** (Atezolizumab + bevacizumab)	1	0.78	0.64 - 0.96	.016	NA	⊕⊕⊕O Moderate[b]
**Second/third-line ICI vs CT**	6	0.70	0.64 - 0.77	< .0001	0	⊕⊕⊕⊕ High

© 2019 Yu Y et al. *JAMA Network Open.*

Atezolizumab	2	0.73	0.63 - 0.85	< .0001	0	⊕⊕⊕O Moderate[b]
Nivolumab	3	0.69	0.61 - 0.79	< .0001	0	⊕⊕⊕⊕ High
Pembrolizumab	1	0.67	0.56 - 0.80	< .0001	NA	⊕⊕⊕O Moderate[b]
**Tumor vaccine**						
**All trials**	8	0.83	0.76 - 0.91	< .0001	0	⊕⊕⊕⊕ High
**First-line TV+CT vs CT**	4	0.84	0.68 - 1.03	.100	0	⊕⊕⊕O Moderate[c]
**First-line MT TV vs no TV**	6	0.83	0.74 - 0.92	.001	7.7	⊕⊕⊕⊕ High
**Second-line TV+CT vs CT**	1	0.80	0.42 - 1.52	.496	NA	⊕⊕OO Low[b,c]
**Cellular immunotherapy**						
**First-line CIM+CT vs CT**	2	0.40	0.17 - 0.96	.038	67.6	⊕⊕OO Low[a,b]

Abbreviations: GRADE, Grading of Recommendations Assessment, Development and Evaluation; vs, versus; ICI, immune checkpoint inhibitor; TV, tumor vaccine; CIM, cellular immunotherapy; CT, chemotherapy; MT, maintenance therapy; HR, hazard ratio; CI, confidence interval; VEGFR, vascular endothelial growth factor receptor; NA, not available.

[a] Downgraded (-1) for inconsistency: Substantial heterogeneity (I^2 > 50%) was found among the trials.

[b] Downgraded (-1) for imprecision: Fewer than three trials were included in this subgroup.

[c] Downgraded (-1) for imprecision: The 95% CIs were so wide that the result included no effect and failed to exclude important benefits or serious harmful effects.

GRADE Working Group grades of evidence:

⊕⊕⊕⊕ High quality: Further research is very unlikely to change our confidence in the estimated effect.

⊕⊕⊕O Moderate quality: Further research is likely to have an important impact on our confidence in the estimated effect and might change the estimate.

⊕⊕OO Low quality: Further research is very likely to have an important impact on our confidence in the estimated effect and might change the estimate.

⊕OOO Very low quality: We are very uncertain about the estimate.

eTable 5. Summary of the Pooled Estimates and Grading of Recommendations, Assessment, Development, and Evaluation Evidence of Objective Response Rate

**Subgroup**	**Objective Response Rate**					
	**No. of**	**RR**	**95% CI**	***P* Value**	**I^2, %**	**GRADE**

© 2019 Yu Y et al. *JAMA Network Open.*

	Trials					
**Immune checkpoint inhibitor**						
All trials	16	1.34	1.26 - 1.42	<. 0001	83.6	⊕⊕⊕O Moderate[a]
First-line therapy	10	1.28	1.20 - 1.37	<. 0001	75.4	⊕⊕⊕O Moderate[a]
Second-line therapy	5	2.00	1.66 - 2.41	<. 0001	86.3	⊕⊕⊕O Moderate[a]
Maintenance therapy	1	1.02	0.87 - 1.19	.8119	NA	⊕⊕OO Low[b,c]
**Tumor vaccine**						
All trials	9	1.04	0.98 - 1.12	.201	58.3	⊕⊕OO Low[a,c]
First-line therapy	4	1.48	1.16 - 1.88	.0014	0	⊕⊕⊕⊕ High
Second-line therapy	1	2.77	0.62 - 12.42	.1835	NA	⊕⊕OO Low[b,c]
Maintenance therapy	4	0.98	0.92 - 1.05	.6197	44.9	⊕⊕⊕O Moderate[c]
**Cellular immunotherapy**						
First-line therapy	1	1.03	0.58 - 1.84	.908	NA	⊕⊕OO Low[b,c]

Abbreviations: GRADE, Grading of Recommendations Assessment, Development and Evaluation; RR, risk ratio; CI, confidence interval; NA, not available.

[a] Downgraded (-1) for inconsistency: Substantial heterogeneity (I^2 > 50%) was found among the trials.

[b] Downgraded (-1) for imprecision: Fewer than three trials were included in this subgroup.

[c] Downgraded (-1) for imprecision: The 95% CIs were so wide that the result included no effect and failed to exclude important benefits or serious harmful effects.

GRADE Working Group grades of evidence:

⊕⊕⊕⊕ High quality: Further research is very unlikely to change our confidence in the estimated effect.

⊕⊕⊕O Moderate quality: Further research is likely to have an important impact on our confidence in the estimated effect and might change the estimate.

⊕⊕OO Low quality: Further research is very likely to have an important impact on our confidence in the estimated effect and might change the estimate.

⊕OOO Very low quality: We are very uncertain about the estimate.

© 2019 Yu Y et al. *JAMA Network Open.*

eTable 6. Summary of the Estimates and Grading of Recommendations, Assessment, Development, and Evaluation Evidence in the Subgroup Analysis of Clinical Outcomes

**Quality Assessment**								**Effect**	**Quality**	**Importance**
**No. of trials**	**Design**	**Risk of Bias**	**Inconsistency**	**Indirectness**	**Imprecision**	**Publication Bias**	**Other Considerations**	**Relative (95% CI)**		
**Objective response rate among all trials**										
26	Randomized trials	No serious risk of bias	Serious	No serious indirectness	No serious imprecision	Strongly suspected	None	RR 1.33 (1.18 - 1.51)	⊕⊕OO Low[a,c]	CRITICAL
**Objective response rate among the trials investigating immune checkpoint inhibitor therapy**										
16	Randomized trials	No serious risk of bias	Serious	No serious indirectness	No serious imprecision	Strongly suspected	None	RR 1.47 (1.25 - 1.73)	⊕⊕OO Low[a,c]	CRITICAL
**Progression-free survival outcomes among the trials investigating immune checkpoint inhibitor therapy**										
**Age < 65**										
7	Randomized trials	No serious risk of bias	Serious	No serious indirectness	No serious imprecision	None detected	None	HR 0.65 (0.49 - 0.86)	⊕⊕⊕O Moderate[a]	CRITICAL
**Age ≥ 65**										

© 2019 Yu Y et al. *JAMA Network Open.*

7	Randomized trials	No serious risk of bias	Serious	No serious indirectness	No serious imprecision	None detected	None	HR 0.76 (0.61 - 0.94)	⨁⨁⨁O Moderate[a]	CRITICAL
**ECOG PS = 0**										
6	Randomized trials	No serious risk of bias	Serious	No serious indirectness	Serious	None detected	None	HR 0.76 (0.51 - 1.12)	⨁⨁OO Low[a,d]	CRITICAL
**ECOG PS = 1**										
6	Randomized trials	No serious risk of bias	Serious	No serious indirectness	No serious imprecision	None detected	None	HR 0.68 (0.56 - 0.82)	⨁⨁⨁O Moderate[a]	CRITICAL
**EGFR mutant**										
2	Randomized trials	No serious risk of bias	No serious inconsistencies	No serious indirectness	Serious	None detected	None	HR 0.63 (0.42 - 0.94)	⨁⨁⨁O Moderate[b]	CRITICAL
**EGFR wild-type**										
8	Randomized trials	No serious risk of bias	Serious	No serious indirectness	No serious imprecision	None detected	None	HR 0.64 (0.53 - 0.77)	⨁⨁⨁O Moderate[a]	CRITICAL

© 2019 Yu Y et al. *JAMA Network Open.*

**KRAS mutant**										
1	Randomized trials	No serious risk of bias	No serious inconsistencies	No serious indirectness	Serious	None detected	None	HR 0.50 (0.29 - 0.85)	⊕⊕⊕O Moderate [b]	CRITICAL
**PD-L1 TC0 or IC0**										
7	Randomized trials	No serious risk of bias	No serious inconsistencies	No serious indirectness	No serious imprecision	None detected	None	HR 0.77 (0.67 - 0.89)	⊕⊕⊕⊕ High	CRITICAL
**PD-L1 TC1/2/3 or IC1/2/3**										
8	Randomized trials	No serious risk of bias	Serious	No serious indirectness	No serious imprecision	None detected	None	HR 0.61 (0.51 - 0.74)	⊕⊕⊕O Moderate [a]	CRITICAL
**PD-L1 TC2/3 or IC2/3**										
5	Randomized trials	No serious risk of bias	Serious	No serious indirectness	No serious imprecision	None detected	None	HR 0.65 (0.45 - 0.94)	⊕⊕⊕O Moderate [a]	CRITICAL
**PD-L1 TC3 or IC3**										
12	Randomized trials	No serious	Serious	No serious indirectness	No serious imprecision	None detected	None	HR 0.60 (0.47 -	⊕⊕⊕O Moderate	CRITICAL

© 2019 Yu Y et al. *JAMA Network Open.*

		risk of bias						0.76)	[a]	
**Female**										
6	Randomized trials	No serious risk of bias	Serious	No serious indirectness	Serious	None detected	None	HR 0.74 (0.49 - 1.10)	⊕⊕OO Low[a,d]	CRITICAL
**Male**										
6	Randomized trials	No serious risk of bias	Serious	No serious indirectness	No serious imprecision	None detected	None	HR 0.64 (0.50 - 0.83)	⊕⊕⊕O Moderate[a]	CRITICAL
**Current or former smoker**										
4	Randomized trials	No serious risk of bias	No serious inconsistencies	No serious indirectness	Serious	None detected	None	HR 0.59 (0.51 - 0.69)	⊕⊕⊕O Moderate[b]	CRITICAL
**Never smoked**										
3	Randomized trials	No serious risk of bias	No serious inconsistencies	No serious indirectness	Serious	None detected	None	HR 0.64 (0.43 - 0.97)	⊕⊕⊕O Moderate[b]	CRITICAL
**Squamous-type tumor**										

© 2019 Yu Y et al. *JAMA Network Open.*

8	Randomized trials	No serious risk of bias	No serious inconsistencies	No serious indirectness	No serious imprecision	None detected	None	HR 0.73 (0.64 - 0.84)	⊕⊕⊕⊕ High	CRITICAL
**Nonsquamous-type tumor**										
8	Randomized trials	No serious risk of bias	Serious	No serious indirectness	No serious imprecision	None detected	None	HR 0.70 (0.55 - 0.88)	⊕⊕⊕O Moderate[a]	CRITICAL
**High tumor mutation burden**										
2	Randomized trials	No serious risk of bias	No serious inconsistencies	No serious indirectness	Serious	None detected	None	HR 0.58 (0.46 - 0.74)	⊕⊕⊕O Moderate[b]	CRITICAL
**Low tumor mutation burden**										
2	Randomized trials	No serious risk of	Serious	No serious indirectness	Serious	None detected	None	HR 1.20 (0.81 - 1.79)	⊕OOO Very Low[a,b,d]	CRITICAL
**Overall survival outcomes among the trials investigating immune checkpoint inhibitor therapy**										
**Age < 65**										
8	Randomized trials	No serious risk of bias	Serious	No serious indirectness	No serious imprecision	None detected	None	HR 0.73 (0.62 - 0.87)	⊕⊕⊕O Moderate[a]	CRITICAL

© 2019 Yu Y et al. *JAMA Network Open.*

**Age ≥ 65**										
8	Randomized trials	No serious risk of bias	No serious inconsistencies	No serious indirectness	No serious imprecision	None detected	None	HR 0.80 (0.70 - 0.90)	⊕⊕⊕⊕ High	CRITICAL
**ECOG PS = 0**										
8	Randomized trials	No serious risk of bias	No serious inconsistencies	No serious indirectness	No serious imprecision	None detected	None	HR 0.75 (0.63 - 0.9)	⊕⊕⊕⊕ High	CRITICAL
**ECOG PS = 1**										
8	Randomized trials	No serious risk of bias	Serious	No serious indirectness	No serious imprecision	None detected	None	HR 0.75 (0.66 - 0.85)	⊕⊕⊕O Moderate [a]	CRITICAL
**ALK wild-type**										
1	Randomized trials	No serious risk of bias	No serious inconsistencies	No serious indirectness	Serious	None detected	None	HR 0.49 (0.38 - 0.64)	⊕⊕⊕O Moderate [b]	CRITICAL
**EGFR mutant**										
3	Randomized trials	No serious	No serious inconsistencies	No serious indirectness	Serious	None detected	None	HR 1.12 (0.80 -	⊕⊕⊕O Moderate	CRITICAL

© 2019 Yu Y et al. *JAMA Network Open.*

		risk of bias	s					1.56)	[d]	
**EGFR wild−type**										
8	Randomized trials	No serious risk of bias	Serious	No serious indirectness	No serious imprecision	None detected	None	HR 0.68 (0.58 - 0.79)	⊕⊕⊕O Moderate[a]	CRITICAL
**KRAS mutant**										
3	Randomized trials	No serious risk of bias	No serious inconsistencies	No serious indirectness	No serious imprecision	None detected	None	HR 0.65 (0.44 - 0.96)	⊕⊕⊕⊕ High	CRITICAL
**KRAS wild-type**										
4	Randomized trials	No serious risk of bias	No serious inconsistencies	No serious indirectness	No serious imprecision	None detected	None	HR 0.81 (0.69 - 0.95)	⊕⊕⊕⊕ High	CRITICAL
**PD-L1 TC0 or IC0**										
7	Randomized trials	No serious risk of bias	No serious inconsistencies	No serious indirectness	No serious imprecision	None detected	None	HR 0.72 (0.61 - 0.86)	⊕⊕⊕⊕ High	CRITICAL
**PD-L1 TC1/2/3 or IC1/2/3**										

© 2019 Yu Y et al. *JAMA Network Open.*

8	Randomized trials	No serious risk of bias	Serious	No serious indirectness	No serious imprecision	None detected	None	HR 0.60 (0.46 - 0.79)	⊕⊕⊕O Moderate[a]	CRITICAL
**PD-L1 TC2/3 or IC2/3**										
7	Randomized trials	No serious risk of bias	Serious	No serious indirectness	No serious imprecision	None detected	None	HR 0.64 (0.51 - 0.81)	⊕⊕⊕O Moderate[a]	CRITICAL
**PD-L1 TC3 or IC3**										
11	Randomized trials	No serious risk of bias	No serious inconsistencies	No serious indirectness	No serious imprecision	None detected	None	HR 0.55 (0.47 - 0.65)	⊕⊕⊕⊕ High	CRITICAL
**Female**										
8	Randomized trials	No serious risk of bias	Serious	No serious indirectness	No serious imprecision	None detected	None	HR 0.71 (0.54 - 0.95)	⊕⊕⊕O Moderate[a]	CRITICAL
**Male**										
8	Randomized trials	No serious risk of bias	No serious inconsistencies	No serious indirectness	No serious imprecision	None detected	None	HR 0.78 (0.72 - 0.85)	⊕⊕⊕⊕ High	CRITICAL

© 2019 Yu Y et al. *JAMA Network Open.*

**Current or former smoker**										
7	Randomized trials	No serious risk of bias	Serious	No serious indirectness	No serious imprecision	None detected	None	HR 0.79 (0.69 - 0.90)	⊕⊕⊕O Moderate[a]	CRITICAL
**Never smoked**										
6	Randomized trials	No serious risk of bias	Serious	No serious indirectness	Serious	None detected	None	HR 0.75 (0.53 - 1.06)	⊕⊕OO Low[a,d]	CRITICAL
**Squamous-type tumor**										
7	Randomized trials	No serious risk of bias	No serious inconsistencies	No serious indirectness	No serious imprecision	None detected	None	HR 0.77 (0.69 - 0.86)	⊕⊕⊕⊕ High	CRITICAL
**Nonsquamous-type tumor**										
8	Randomized trials	No serious risk of bias	Serious	No serious indirectness	No serious imprecision	None detected	None	HR 0.77 (0.67 - 0.88)	⊕⊕⊕O Moderate[a]	CRITICAL

Abbreviations: GRADE, Grading of Recommendations Assessment, Development and Evaluation; RR, risk ratio; HR, hazard ratio; CI, confidence interval; ECOG PS, Eastern Cooperative Oncology Group Performance Status; ALK, anaplastic lymphoma kinase; EGFR, epidermal growth factor receptor; KRAS, kirsten rat sarcoma viral oncogene homolog; PD-L1, programmed death-ligand 1; TC, tumor cells; IC, tumor-infiltrating immune cells.

[a] Downgraded (-1) for inconsistency: Substantial heterogeneity (I^2 > 50%) was found among the trials.

© 2019 Yu Y et al. *JAMA Network Open.*

[b] Downgraded (-1) for imprecision: Fewer than three trials were included in this subgroup.
[c] Downgraded (-1) for publication bias: The *P* values of the Egger's and Begg's regression tests were < 0.05 in this subgroup.
[d] Downgraded (-1) for imprecision: The 95% CIs were so wide that the result included no effect and failed to exclude important benefits or serious harmful effects.
PD-L1 scoring criteria:
TC3 or IC3: TC3 ≥50% or IC3 ≥10%.
TC2 or IC2: TC2 ≥5% and <50% or IC2 ≥5% and <10%.
TC1 or IC1: TC1 ≥1% and <5% or IC1 ≥1% and <5%.
TC0 or IC0: TC0 <1% or IC0 <1%.
GRADE Working Group grades of evidence:
⊕⊕⊕⊕ High quality: Further research is very unlikely to change our confidence in the estimate of effect.
⊕⊕⊕O Moderate quality: Further research is likely to have an important impact on our confidence in the estimate of effect and may change the estimate.
⊕⊕OO Low quality: Further research is very likely to have an important impact on our confidence in the estimate of effect and is likely to change the estimate.
⊕OOO Very low quality: We are very uncertain about the estimate.

eTable 7. Summary of the Estimates Stratified by Programmed Cell Death Ligand 1 Expression and Treatment Strategy

	**Overall Survival**						**Progression-free Survival**					
**Subgroup**	**No. of Trials**	**HR**	**95% CI**	***I*² (%)**	***P* Value**	***P* for Interaction [a]**	**No. of Trials**	**HR**	**95% CI**	***I*² (%)**	***P* Value**	***P* for Interaction [a]**
**TC3 or IC3**						.6381						.0002
ICI vs CT	8	0.57	0.46 - 0.71	55.9	< .0001		8	0.73	0.58 - 0.94	73.8	.0123	
ICI+CT vs CT	3	0.52	0.38 - 0.71	0	< .0001		3	0.38	0.30 - 0.49	0	< .0001	
**TC2/3 or IC2/3**						.5361						.1726
ICI vs CT	6	0.65	0.51 - 0.84	74.5	.0011		4	0.79	0.56 - 1.09	82.6	.1551	

© 2019 Yu Y et al. *JAMA Network Open.*

ICI+CT vs CT	1	0.55	0.34 - 0.90	NA	.016		1	0.55	0.37 - 0.81	NA	< .0001	
**TC1/2/3 or IC1/2/3**						.0296						.002
ICI vs CT	6	0.70	0.61 - 0.79	30.6	< .0001		4	0.85	0.69 - 1.05	75.7	.1204	
ICI+CT vs CT	2	0.50	0.38 - 0.66	0	< .001		3	0.53	0.43 - 0.66	41.9	< .0001	
**TC0 or IC0**						.9536						.5751
ICI vs CT	4	0.72	0.56 - 0.94	48.2	.0166		2	0.90	0.51 - 1.61	79.8	.719	
ICI+CT vs CT	3	0.72	0.55 - 0.93	26.2	.0115		4	0.76	0.66 - 0.87	0	< .0001	

Abbreviations: HR, hazard ratio; CI, confidence interval; PD-L1, programmed death-ligand 1; TC, tumor cells; IC, tumor-infiltrating immune cells; ICI, immune checkpoint inhibitor; CT, chemotherapy.

[a] The *P* value for interaction reflects the difference between ICI alone and ICI + CT, as calculated by the χ^2 test comparing the HRs of the subgroups; NA, not available.

PD-L1 scoring criteria:

TC3 or IC3: TC3 ≥50% or IC3 ≥10%.

TC2 or IC2: TC2 ≥5% and <50% or IC2 ≥5% and <10%.

TC1 or IC1: TC1 ≥1% and <5% or IC1 ≥1% and <5%.

TC0 or IC0: TC0 <1% or IC0 <1%.

© 2019 Yu Y et al. *JAMA Network Open.*

eFigure 1. PRISMA Flow Diagram for the Meta-analysis

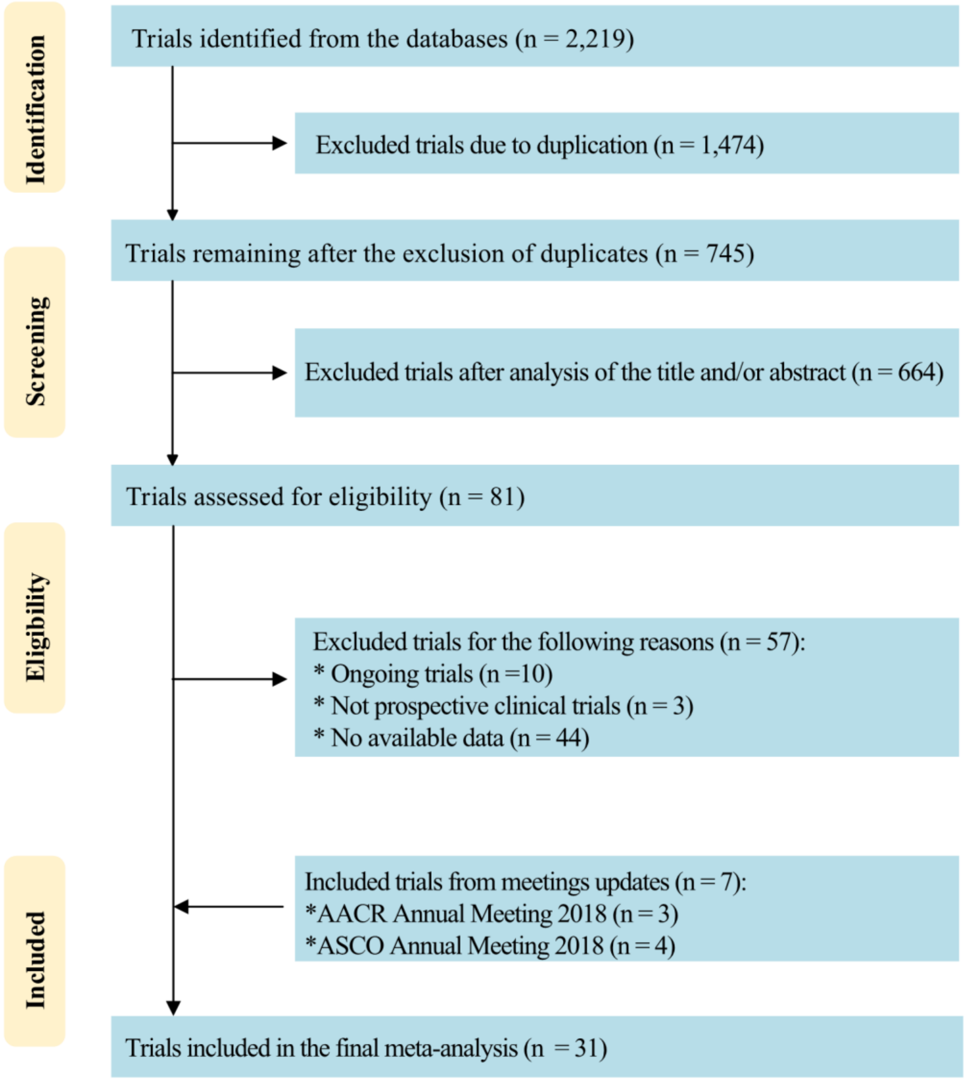

PRISMA, Preferred Reporting Items for Systematic Reviews and Meta-Analyses; ASCO, the American Society of Clinical Oncology; AACR, the American Association for Cancer Research.

eFigure 2. Risk of Bias Summary of the Randomized Trials Included in the Meta-analysis

© 2019 Yu Y et al. *JAMA Network Open.*

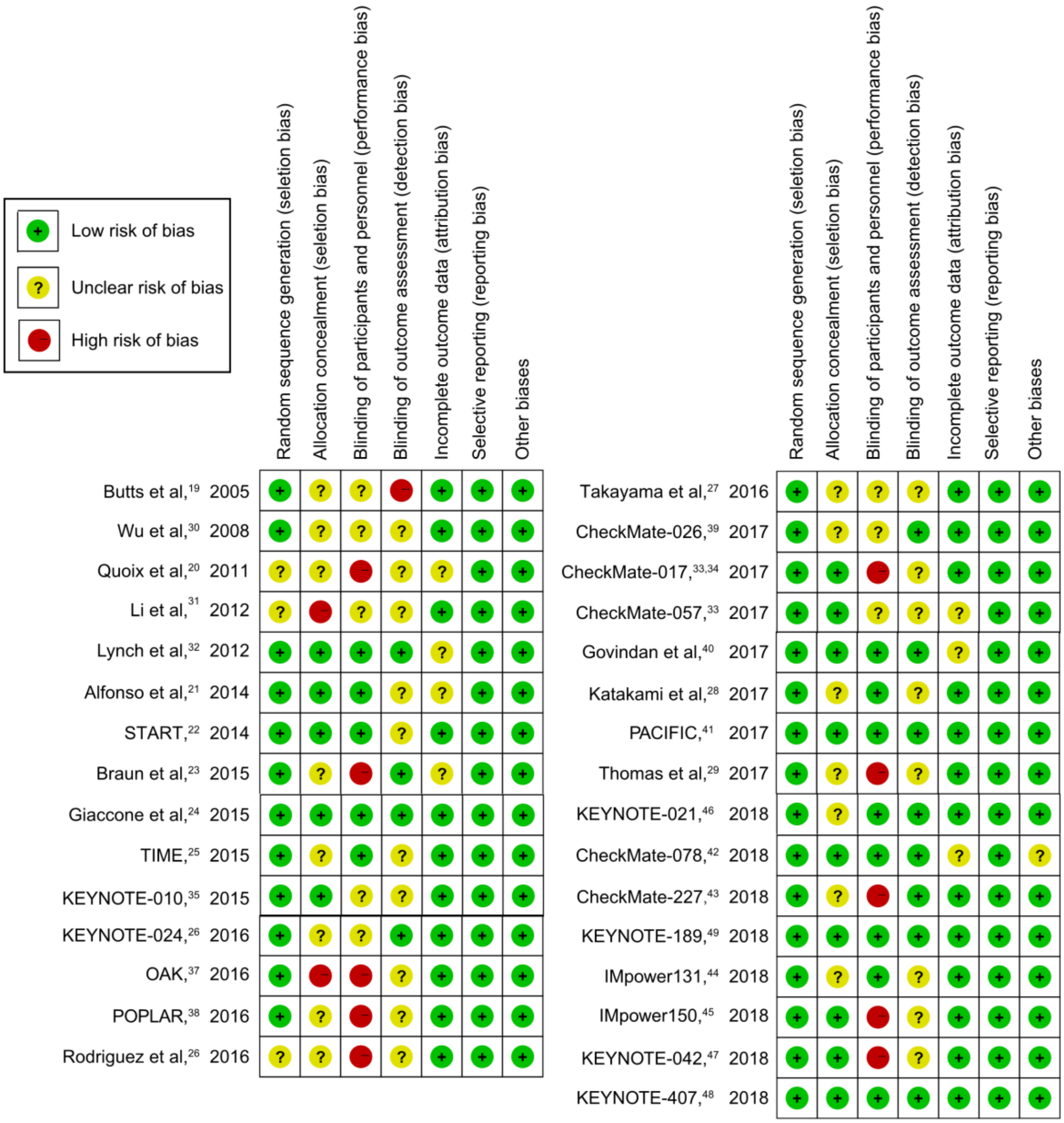

.

eFigure 3. Risk of bias graph for the Randomized Trials Included in the Meta-analysis

© 2019 Yu Y et al. *JAMA Network Open.*

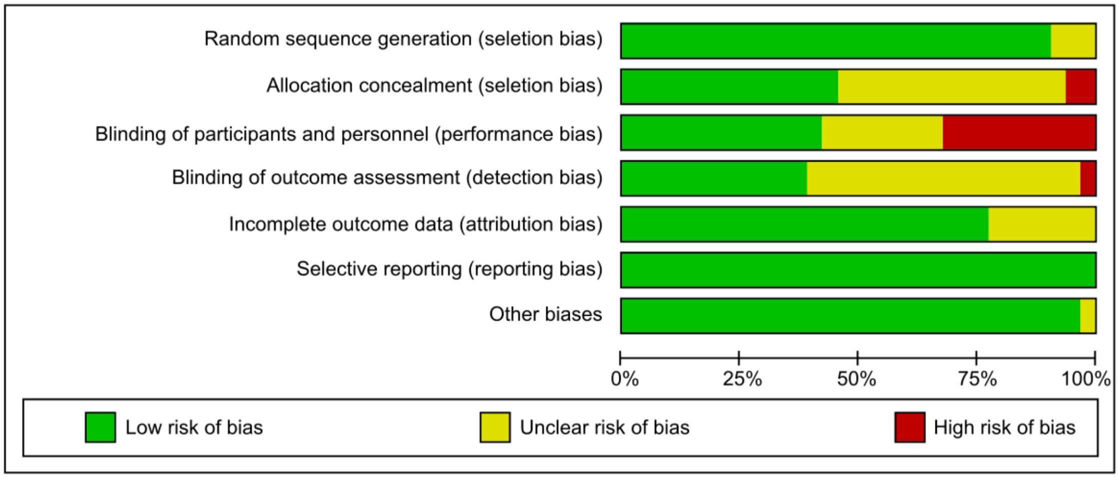

eFigure 4. Analysis of Publication Bias in the Meta-analyses of Immunotherapy vs Conventional Therapy

© 2019 Yu Y et al. *JAMA Network Open.*

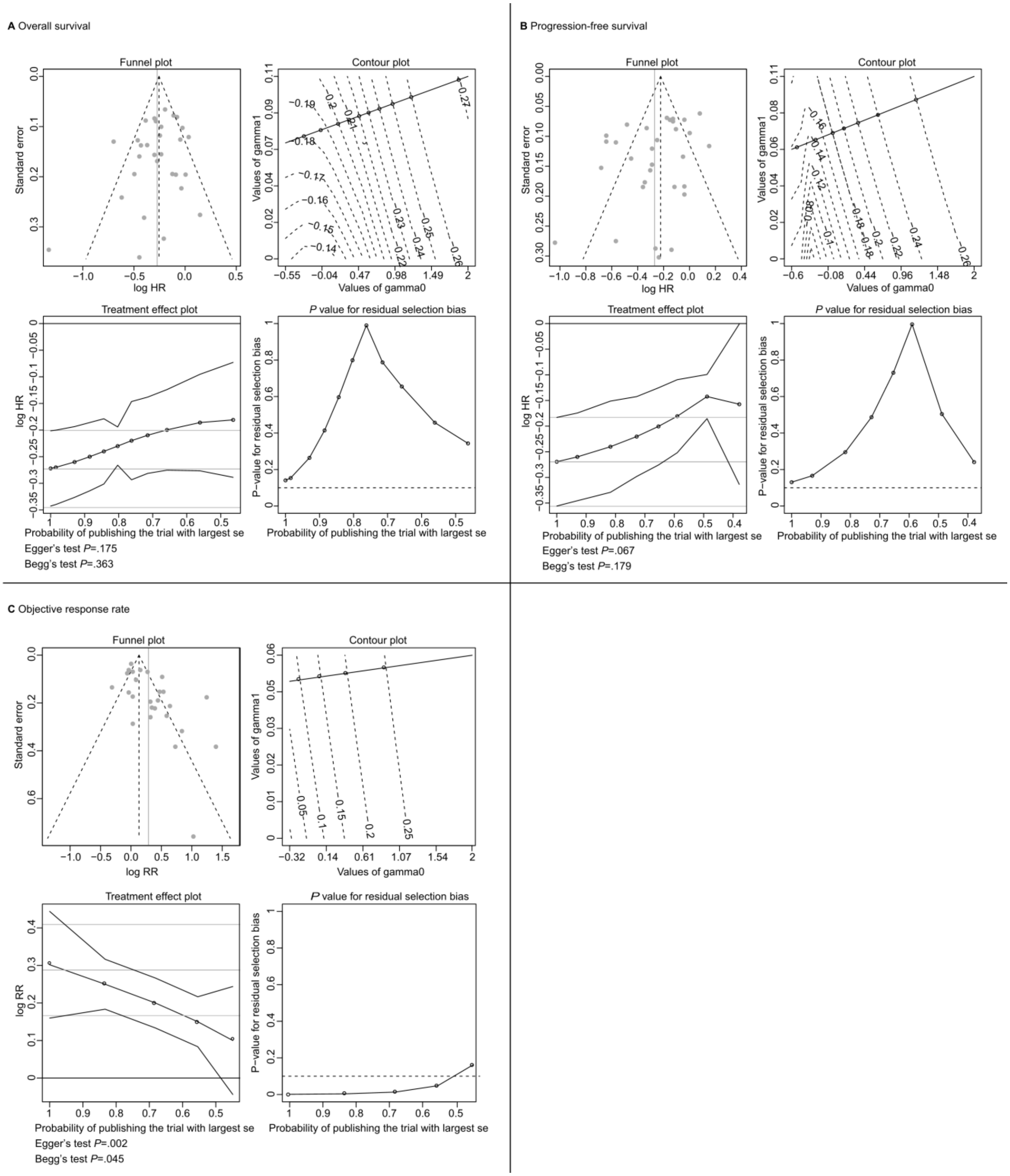

The outcomes assessed were overall survival (A), progression-free survival (B) and the objective response rate (C). HR, hazard ratio; RR, risk ratio.

eFigure 5. Pooled Analysis of the Ratio of the Median Overall Survival With Immunotherapy vs Conventional Therapy

© 2019 Yu Y et al. *JAMA Network Open.*

Study or Subgroup	Immunotherapy Total	Conventional Therapy Total	Median Overall Survival Ratio (95% CI)	Weight, %
Immune checkpoint inhibitors				
Lynch et al,[32,a] 2012	70	66	1.17 (0.99-1.38)	3.8
Lynch et al,[32,b] 2012	68	66	1.47 (1.24-1.74)	3.8
KEYNOTE-010,[35,c] 2015	346	343	1.22 (1.13-1.32)	4.0
KEYNOTE-010,[35,d] 2015	344	343	1.58 (1.47-1.70)	4.0
OAK,[37] 2016	425	425	1.44 (1.34-1.54)	4.0
POPLAR,[38] 2016	144	143	1.30 (1.16-1.46)	3.9
CheckMate-017,[33,34] 2017	135	137	1.53 (1.36-1.73)	3.9
CheckMate-026,[39] 2017	211	212	1.09 (0.99-1.20)	4.0
CheckMate-057,[33] 2017	292	290	1.30 (1.20-1.41)	4.0
Govindan et al,[40] 2017	388	361	1.08 (1.01-1.16)	4.0
IMpower131,[44] 2018	343	340	1.01 (0.93-1.09)	4.0
IMpower150,[45] 2018	359	337	0.77 (0.71-0.82)	4.0
KEYNOTE-021,[46] 2018	60	63	2.58 (2.16-3.08)	3.7
KEYNOTE-042,[47] 2018	637	637	1.38 (1.31-1.46)	4.0
Subtotal	3,822	3,763	1.30 (1.16-1.46)	59.0
Heterogeneity: $\tau^2 = 0.05$; $\chi^2_{13} = 370.93$ ($P < .01$); $I^2 = 97\%$				
Test for overall effect: $z = 4.10$ ($P < .01$)				
Tumor vaccine				
Butts et al,[19] 2005	88	83	1.34 (1.15-1.55)	3.8
Quoix et al,[20] 2011	74	74	1.04 (0.88-1.22)	3.8
Alfonso et al,[21] 2014	87	89	1.21 (1.04-1.40)	3.8
START,[22] 2014	829	410	1.15 (1.09-1.21)	4.0
Braun et al,[23] 2015	61	31	1.39 (1.13-1.70)	3.6
Giaccone et al,[24] 2015	270	262	2.76 (2.53-3.00)	4.0
Rodriguez et al,[25] 2016	270	135	0.57 (0.52-0.63)	4.0
Takayama et al,[27] 2016	26	24	1.37 (1.04-1.81)	3.3
Thomas et al,[29] 2017	60	30	0.83 (0.68-1.02)	3.6
Subtotal	1,765	1,138	1.19 (0.84-1.69)	34.0
Heterogeneity: $\tau^2 = 0.28$; $\chi^2_{8} = 613.99$ ($P < .01$); $I^2 = 99\%$				
Test for overall effect: $z = 0.98$ ($P = .33$)				
Cellular immunotherapy				
Wu et al,[30] 2008	29	30	1.36 (1.06-1.76)	3.4
Li et al,[31] 2012	37	37	2.40 (1.91-3.01)	3.5
Subtotal	66	67	1.81 (1.04-3.16)	7.0
Heterogeneity: $\tau^2 = 0.14$; $\chi^2_{1} = 10.49$ ($P < .01$); $I^2 = 91\%$				
Test for overall effect: $z = 2.11$ ($P = .04$)				
Total	5,653	4,968	1.29 (1.14-1.46)	100.0
Heterogeneity: $\tau^2 = 0.10$; $\chi^2_{24} = 1,017.92$ ($P < .01$); $I^2 = 98\%$				
Test for overall effect: $z = 3.91$ ($P < .01$)				

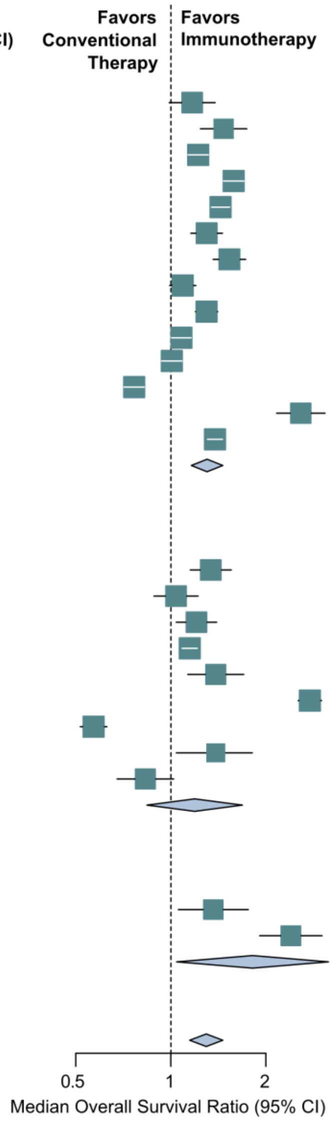

Size of boxes is proportional to the weight of each trial, and the diamonds indicate the point estimate and confidence interval of the combined result.

[a] Patients were treated by concurrent regimen.

[b] Patients were treated by phased regimen.

[c] Drugs were administered at a dose of 2 mg/kg.

[d] Drugs were administered at a dose of 10 mg/kg.

eFigure 6. Pooled Hazard Ratios for Progression-Free Survival With Immunotherapy vs Conventional Therapy

© 2019 Yu Y et al. *JAMA Network Open.*

Study or Subgroup	Immunotherapy Total	Conventional Therapy Total	Hazard Ratio (95% CI)	Weight, %
**Immune checkpoint inhibitors**				
Lynch et al,[32,a] 2012	68	66	0.69 (0.48-1.00)	2.6
Lynch et al,[32,b] 2012	70	66	0.88 (0.61-1.27)	2.6
KEYNOTE-010,[35] 2015	690	343	0.85 (0.73-0.98)	4.2
KEYNOTE-024,[36] 2016	154	151	0.50 (0.37-0.68)	3.0
OAK,[37] 2016	425	425	0.95 (0.82-1.10)	4.2
POPLAR,[38] 2016	144	143	0.94 (0.72-1.23)	3.3
CheckMate-017,[33,34] 2017	135	137	0.63 (0.48-0.83)	3.3
CheckMate-057,[33] 2017	292	290	0.89 (0.75-1.06)	4.0
CheckMate-026,[39] 2017	211	212	1.15 (0.91-1.45)	3.6
Govindan et al,[40] 2017	388	361	0.87 (0.75-1.01)	4.2
PACIFIC,[41] 2017	473	236	0.52 (0.42-0.65)	3.7
CheckMate-078,[42] 2018	338	166	0.77 (0.62-0.95)	3.7
CheckMate-227,[43,c] 2018	583	583	0.83 (0.72-0.96)	4.3
CheckMate-227,[43,d] 2018	71	79	0.95 (0.64-1.41)	2.4
CheckMate-227,[43,e] 2018	177	186	0.74 (0.58-0.94)	3.5
IMpower131,[44] 2018	343	340	0.71 (0.60-0.85)	4.0
IMpower150,[45] 2018	400	400	0.61 (0.52-0.72)	4.1
KEYNOTE-021,[46] 2018	60	63	0.56 (0.33-0.96)	1.7
KEYNOTE-042,[47] 2018	637	637	1.07 (0.94-1.21)	4.4
KEYNOTE-189,[49] 2018	410	206	0.52 (0.43-0.63)	3.8
KEYNOTE-407,[48] 2018	278	281	0.56 (0.45-0.70)	3.7
Subtotal	6,347	5,371	0.76 (0.68-0.84)	74.3
Heterogeneity: $\tau^2 = 0.05$; $\chi^2_{20} = 111.01$ ($P < .01$); $I^2 = 82\%$				
Test for overall effect: $z = -5.13$ ($P < .01$)				
**Tumor vaccine**				
Quoix et al,[20] 2011	74	74	0.70 (0.49-0.99)	2.7
Alfonso et al,[21] 2014	87	89	0.73 (0.53-1.00)	3.0
START,[22] 2014	829	410	0.87 (0.75-1.00)	4.3
Giaccone et al,[24] 2015	270	262	0.99 (0.82-1.20)	3.9
Braun et al,[23] 2015	61	31	0.86 (0.49-1.52)	1.6
TIME,[25] 2015	111	111	0.74 (0.55-0.99)	3.1
Takayama et al,[27] 2016	26	24	0.78 (0.43-1.42)	1.5
Katakami et al,[28] 2017	114	58	0.95 (0.66-1.37)	2.6
Subtotal	1,572	1,059	0.86 (0.78-0.94)	22.5
Heterogeneity: $\tau^2 = 0.00$; $\chi^2_{7} = 5.89$ ($P = .55$); $I^2 = 0\%$				
Test for overall effect: $z = -3.26$ ($P < .01$)				
**Cellular immunotherapy**				
Wu et al,[30] 2008	29	30	0.76 (0.43-1.34)	1.6
Li et al,[31] 2012	37	37	0.35 (0.20-0.61)	1.6
Subtotal	66	67	0.51 (0.24-1.10)	3.2
Heterogeneity: $\tau^2 = 0.22$; $\chi^2_{1} = 3.70$ ($P = .06$); $I^2 = 0\%$				
Test for overall effect: $z = -1.72$ ($P = .09$)				
Total	7,985	6,497	0.76 (0.70-0.83)	100.0
Heterogeneity: $\tau^2 = 0.04$; $\chi^2_{30} = 128.15$ ($P < .01$); $I^2 = 77\%$				
Test for overall effect: $z = -6.10$ ($P < .01$)				

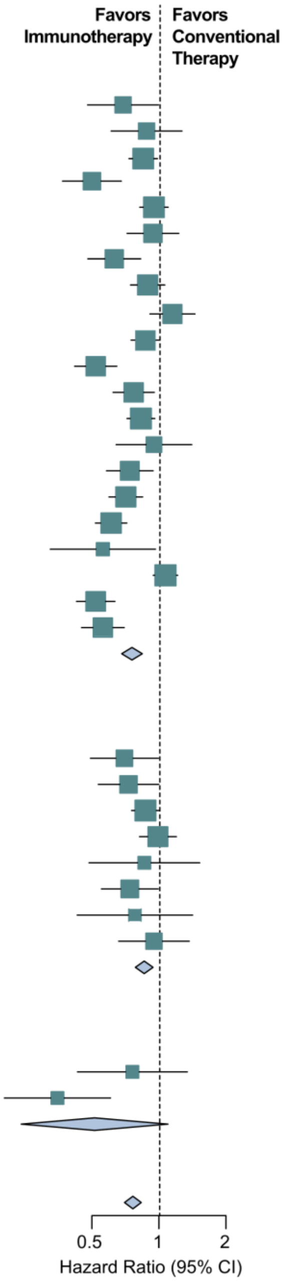

Size of boxes is proportional to the weight of each trial, and the diamonds indicate the point estimate and confidence interval of the combined result.

[a] Patients were treated by phased regimen.

[b] Patients were treated by concurrent regimen.

[c] Comparison between nivolumab + ipilimumab and chemotherapy.

[d] Comparison between nivolumab and chemotherapy.

[e] Comparison between nivolumab + ipilimumab and nivolumab.

eFigure 7. Pooled Analysis of the Ratio of the Median Progression-Free Survival With Immunotherapy vs Conventional Therapy

© 2019 Yu Y et al. *JAMA Network Open.*

Study or Subgroup	Immunotherapy Total	Conventional Therapy Total	Median Progresssion-Free Survival Ratio (95% CI)	Weight, %
Immune checkpoint inhibitors				
Lynch et al,[32,a] 2012	70	66	0.98 (0.83-1.15)	3.4
Lynch et al,[32,b] 2012	68	66	1.22 (1.03-1.44)	3.4
KEYNOTE-010,[35,c] 2015	344	343	0.98 (0.90-1.05)	3.5
KEYNOTE-010,[35,d] 2015	346	343	1.00 (0.93-1.08)	3.5
KEYNOTE-024,[36] 2016	154	151	1.72 (1.53-1.92)	3.5
OAK,[37] 2016	425	425	0.70 (0.65-0.75)	3.5
CheckMate-017,[33,34] 2017	135	137	1.25 (1.11-1.41)	3.5
CheckMate-057,[33] 2017	292	290	0.55 (0.50-0.59)	3.5
CheckMate-026,[39] 2017	211	212	0.71 (0.65-0.78)	3.5
Govindan et al,[40] 2017	388	361	1.00 (0.93-1.07)	3.5
PACIFIC,[41] 2017	473	236	3.00 (2.79-3.23)	3.5
CheckMate-078,[42] 2018	338	166	1.00 (0.92-1.09)	3.5
CheckMate-227,[43,e] 2018	583	583	1.12 (1.07-1.18)	3.6
CheckMate-227,[43,f] 2018	71	79	0.75 (0.64-0.88)	3.4
CheckMate-227,[43,g] 2018	177	186	1.19 (1.08-1.32)	3.5
IMpower131,[44] 2018	343	340	1.12 (1.04-1.21)	3.5
IMpower150,[45] 2018	400	400	1.22 (1.14-1.31)	3.5
KEYNOTE-021,[46] 2018	60	63	1.16 (0.97-1.39)	3.4
KEYNOTE-042,[47] 2018	637	637	0.83 (0.79-0.88)	3.5
KEYNOTE-189,[49] 2018	410	206	1.80 (1.66-1.94)	3.5
KEYNOTE-407,[48] 2018	278	281	1.33 (1.23-1.45)	3.5
Subtotal	6,203	5,571	1.09 (0.93-1.29)	73.4
Heterogeneity: $\tau^2 = 0.14$; $\chi^2_{20} = 1,646.47$ (P <.01); $I^2 = 99\%$				
Test for overall effect: $z = 1.07$ (P =.28)				
Tumor vaccine				
Alfonso et al,[21] 2014	87	89	1.37 (1.18-1.58)	3.4
Braun et al,[23] 2015	61	31	1.17 (0.95-1.43)	3.3
Giaccone et al,[24] 2015	270	262	1.07 (0.99-1.17)	3.5
TIME,[25] 2015	111	111	1.16 (1.01-1.32)	3.5
Takayama et al,[27] 2016	26	24	1.12 (0.85-1.48)	3.1
Katakami et al,[28] 2017	114	58	1.45 (1.25-1.68)	3.4
Subtotal	669	575	1.22 (1.09-1.36)	20.2
Heterogeneity: $\tau^2 = 0.01$; $\chi^2_{5} = 15.94$ (P <.01); $I^2 = 69\%$				
Test for overall effect: $z = 3.52$ (P <.01)				
Cellular immunotherapy				
Wu et al,[30] 2008	29	30	1.42 (1.10-1.84)	3.2
Li et al,[31] 2012	37	37	2.17 (1.73-2.72)	3.2
Subtotal	66	67	1.76 (1.17-2.66)	6.4
Heterogeneity: $\tau^2 = 0.07$; $\chi^2_{1} = 5.78$ (P =.02); $I^2 = 83\%$				
Test for overall effect: $z = 2.70$ (P <.01)				
Total	6,938	6,213	1.15 (1.01-1.32)	100.0
Heterogeneity: $\tau^2 = 0.13$; $\chi^2_{28} = 1,710.22$ (P <.01); $I^2 = 98\%$				
Test for overall effect: $z = 2.06$ (P =.04)				

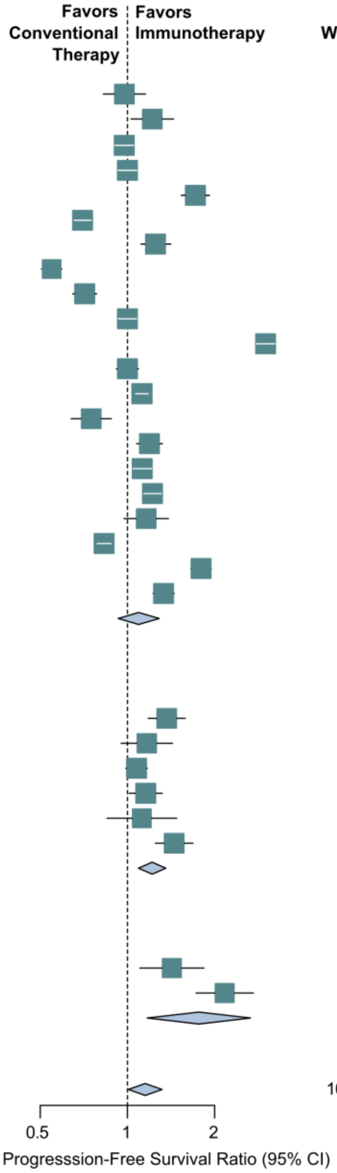

Size of boxes is proportional to the weight of each trial, and the diamonds indicate the point estimate and confidence interval of the combined result.

[a] Patients were treated by concurrent regimen.

[b] Patients were treated by phased regimen.

[c] Drugs were administered at a dose of 2 mg/kg.

[d] Drugs were administered at a dose of 10 mg/kg.

[e] Comparison between nivolumab + ipilimumab and chemotherapy.

[f] Comparison between nivolumab and chemotherapy.

[g] Comparison between nivolumab + ipilimumab and nivolumab.

eFigure 8. Network Diagram of Studies Comparing Clinical Outcomes of Different Immune Checkpoint Inhibitors Strategies for Advanced Non–Small Cell Lung Cancer

© 2019 Yu Y et al. *JAMA Network Open.*

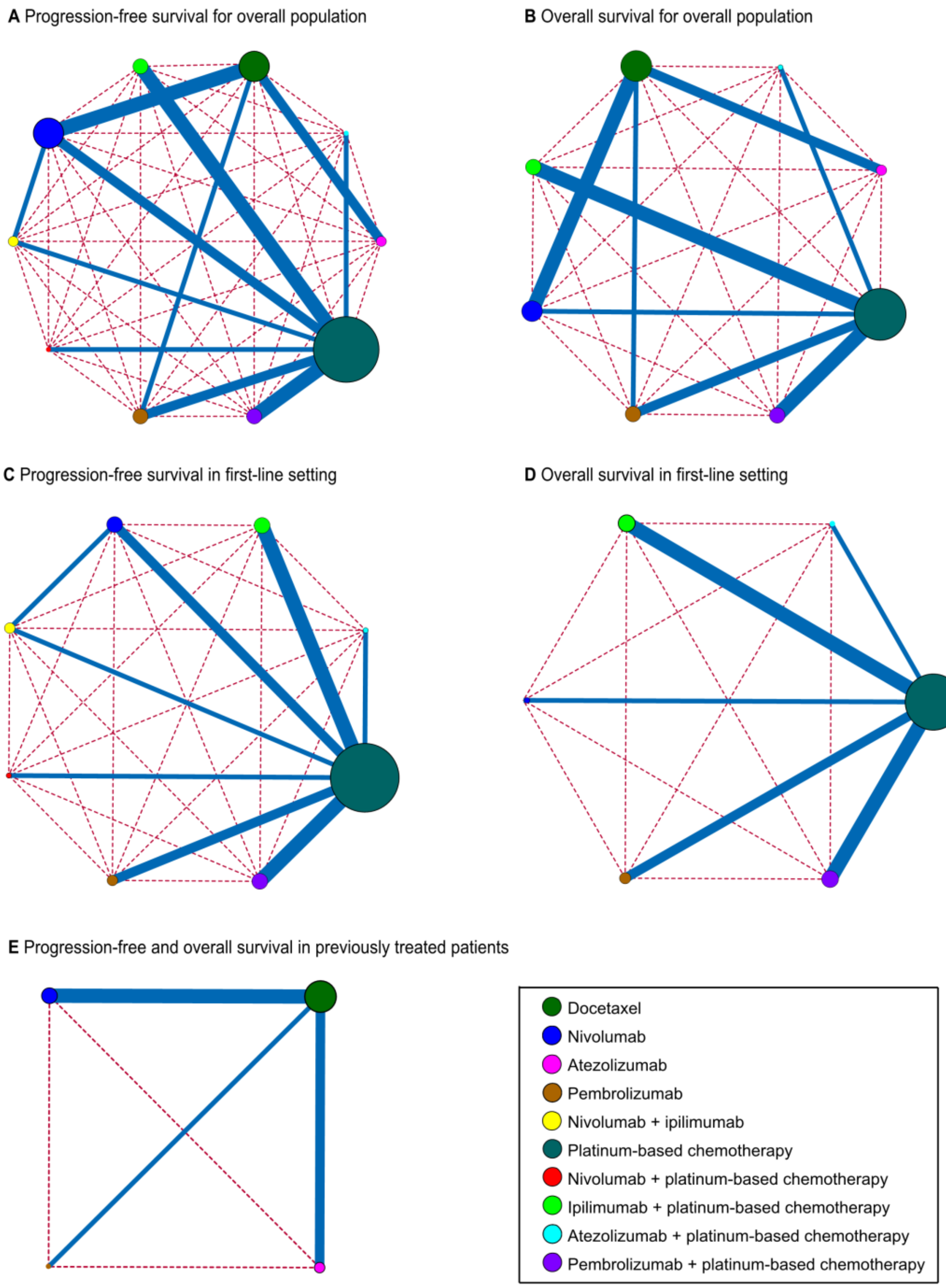

Network diagrams were plotted for (A) progression-free survival for overall population; (B) overall survival for overall population; (C) progression-free survival for first-line therapy; (D) overall survival for first-line therapy; (E) progression-free and overall survival in previously treated patients. The size of connected nodes is proportional to the number of patients receiving the treatment, and the line width is proportional to the number of trials included in the comparison of two treatment groups.

eFigure 9. Network Meta-analysis of Immune Checkpoint Inhibitors in Terms of Progression-Free Survival

© 2019 Yu Y et al. *JAMA Network Open.*

Atezolizumab	1.20 (0.77-1.80)	1.30 (0.82-2.20)	1.40(0.83-2.50)	0.95 (0.68-1.30)	1.60 (0.83-3.10)	1.40 (0.80-2.40)	1.50 (0.79-3.00)	**2.10 (1.20-3.70)**	1.10 (0.71-1.80)
0.86 (0.57-1.30)	Nivolumab	1.10 (0.81-1.60)	1.20(0.84-1.80)	0.82 (0.64-1.00)	1.40 (0.80-2.30)	1.20 (0.79-1.80)	1.30 (0.76-2.30)	**1.80 (1.20-2.70)**	0.98 (0.73-1.30)
0.78 (0.46-1.20)	0.90 (0.62-1.20)	Pembrolizumab	1.10 (0.69-1.70)	**0.73 (0.50-1.00)**	1.20 (0.70-2.10)	1.10 (0.69-1.60)	1.20 (0.66-2.00)	**1.60 (1.00-2.40)**	0.88 (0.64-1.20)
0.70 (0.40-1.20)	0.81 (0.55-1.20)	0.90 (0.59-1.40)	Nivolumab + ipilimumab	0.66 (0.43-1.00)	1.10 (0.63-2.00)	0.97 (0.61-1.50)	1.10 (0.59-1.90)	1.50 (0.90-2.30)	0.80 (0.55-1.10)
1.10 (0.76-1.50)	1.20 (0.96-1.60)	**1.40 (1.00-2.00)**	1.50 (0.98-2.40)	Docetaxel	1.70 (0.96-3.00)	1.50 (0.94-2.30)	1.60 (0.91-2.90)	**2.20 (1.40-3.50)**	1.20 (0.86-1.70)
0.62 (0.32-1.20)	0.72 (0.43-1.20)	0.80 (0.48-1.40)	0.89 (0.51-1.60)	0.59 (0.34-1.00)	Atezolizumab + platinum-based chemotherapy	0.86 (0.51-1.50)	0.96 (0.50-1.90)	1.30 (0.76-2.20)	0.71 (0.45-1.10)
0.72 (0.41-1.20)	0.84 (0.55-1.30)	0.93 (0.63-1.40)	1.00 (0.65-1.60)	0.68 (0.44-1.10)	1.20 (0.67-2.00)	Ipilimumab + platinum-based chemotherapy	1.10(0.63-1.90)	1.50 (0.99-2.30)	0.82 (0.61-1.10)
0.65 (0.33-1.30)	0.75 (0.43-1.30)	0.84 (0.49-1.50)	0.93 (0.51-1.70)	0.61 (0.34-1.10)	1.00 (0.54-2.00)	0.90 (0.52-1.60)	Nivolumab + platinum-based chemotherapy	1.40 (0.77-2.40)	0.74 (0.46-1.20)
**0.48 (0.27-0.84)**	**0.55 (0.37-0.84)**	**0.62 (0.42-0.97)**	0.68 (0.43-1.10)	**0.45 (0.29-0.71)**	0.76 (0.45-1.30)	0.66 (0.44-1.00)	0.73 (0.42-1.30)	Pembrolizumab + platinum-based chemotherapy	**0.54 (0.41-0.73)**
0.88 (0.55-1.40)	1.00 (0.77-1.40)	1.1 (0.87-1.60)	1.30 (0.89-1.80)	0.83 (0.59-1.20)	1.40 (0.89- 2.20)	1.20 (0.91-1.60)	1.40 (0.84-2.20)	**1.80 (1.40-2.50)**	Platinum-based chemotherapy

Comparisons should be read clockwise. Hazard ratios were compared between column-defining and row-defining treatments. Numbers in bold font on a darker cell background are statistically significant.

eFigure 10. Network Meta-analysis of Immune Checkpoint Inhibitors in Terms of Overall Survival

© 2019 Yu Y et al. *JAMA Network Open.*

Atezolizumab	1.00 (0.80-1.30)	1.20 (0.88-1.60)	**0.73 (0.60-0.89)**	0.98 (0.65-1.50)	1.00 (0.72-1.50)	**1.70 (1.20-2.50)**	0.94 (0.69-1.30)
0.98 (0.76-1.30)	Nivolumab	1.10 (0.92-1.50)	**0.71 (0.61-0.83)**	0.96 (0.67-1.40)	1.00 (0.74-1.30)	**1.70 (1.20-2.30)**	0.91 (0.73-1.10)
0.86 (0.63-1.10)	0.88 (0.69-1.10)	Pembrolizumab	**0.63 (0.50-0.76)**	0.84 (0.60-1.10)	0.88 (0.67-1.10)	**1.40 (1.10-1.90)**	**0.80 (0.66-0.95)**
**1.40 (1.10-1.70)**	**1.40 (1.20-1.60)**	**1.60 (1.30-2.00)**	Docetaxel	1.30 (0.94-1.90)	**1.40 (1.00-1.90)**	**2.30 (1.70-3.20)**	**1.30 (1.00-1.60)**
1.00 (0.68-1.50)	1.00 (0.73-1.50)	1.20 (0.87-1.70)	0.75 (0.52-1.10)	Atezolizumab + platinum-based chemotherapy	1.00 (0.75-1.50)	**1.70 (1.20-2.40)**	0.96 (0.73-1.30)
0.98 (0.68-1.40)	1.00 (0.75-1.30)	1.10 (0.90-1.50)	**0.71 (0.53-0.96)**	0.96 (0.69-1.30)	Ipilimumab + platinum-based chemotherapy	**1.70 (1.30-2.20)**	0.92 (0.76-1.10)
**0.59 (0.41- 0.85)**	**0.61 (0.44-0.82)**	**0.69 (0.53-0.91)**	**0.43 (0.31-0.58)**	**0.58 (0.41-0.81)**	**0.60 (0.46-0.79)**	Pembrolizumab + platinum-based chemotherapy	**0.55 (0.45-0.68)**
1.10 (0.79-1.40)	1.10 (0.87-1.40)	**1.20 (1.10-1.50)**	**0.78 (0.62-0.98)**	1.00 (0.79-1.40)	1.10 (0.91-1.30)	**1.80 (1.50-2.20)**	Platinum-based chemotherapy

Comparisons should be read clockwise. Hazard ratios were compared between column-defining and row-defining treatments. Numbers in bold font on a darker cell background are statistically significant.

eFigure 11. Network Meta-analysis of Immune Checkpoint Inhibitors as a First-line Therapy in Terms of Progression-Free Survival

Nivolumab	1.40 (0.79-2.50)	1.30 (0.81-2.10)	1.50 (0.74-3.00)	1.30 (0.77-2.20)	1.40 (0.71-2.90)	**2.00 (1.10-3.30)**	1.10 (0.72-1.60)
0.74 (0.40-1.30)	Pembrolizumab	0.96 (0.50-1.70)	1.10 (0.52-2.20)	0.96 (0.54-1.60)	1.10 (0.50-2.10)	1.50 (0.80-2.40)	0.79 (0.50-1.20)
0.76 (0.48-1.20)	1.00 (0.58-2.00)	Nivolumab + ipilimumab	1.20 (0.55-2.40)	1.00 (0.56-1.80)	1.10 (0.52-2.30)	1.50 (0.84-2.70)	0.82 (0.52-1.30)
0.66 (0.33-1.30)	0.90 (0.46-1.90)	0.86 (0.42-1.80)	Atezolizumab + platinum-based chemotherapy	0.86 (0.44-1.70)	0.96 (0.42-2.20)	1.30 (0.66-2.60)	0.71 (0.40-1.30)
0.76 (0.45-1.30)	1.00 (0.62-1.90)	1.00 (0.56-1.80)	1.20 (0.58-2.30)	Ipilimumab + platinum-based chemotherapy	1.10 (0.55-2.20)	1.50 (0.90-2.50)	0.82 (0.57-1.20)
0.69 (0.34-1.40)	0.94 (0.47-2.00)	0.90 (0.43-1.90)	1.00 (0.46-2.40)	0.90 (0.46-1.80)	Nivolumab + platinum-based chemotherapy	1.40 (0.68-2.70)	0.74 (0.41-1.40)
**0.51 (0.30-0.87)**	0.69 (0.41-1.30)	0.66 (0.37-1.20)	0.76 (0.39-1.50)	0.66 (0.40-1.10)	0.73 (0.37-1.50)	Pembrolizumab + platinum-based chemotherapy	**0.54 (0.38-0.78)**
0.93 (0.64-1.40)	1.30 (0.87-2.00)	1.20 (0.77-1.90)	1.40 (0.79-2.50)	1.20 (0.86-1.80)	1.30 (0.74-2.40)	**1.80 (1.30-2.60)**	Platinum-based chemotherapy

Comparisons should be read clockwise. Hazard ratios were compared between column-defining and row-defining treatments. Numbers in bold font on a darker cell background are statistically significant.

eFigure 12 Network Meta-analysis of Immune Checkpoint Inhibitors as a First-line Therapy in Terms of Overall Survival

Nivolumab	1.30 (0.84-2.30)	1.10 (0.59-1.90)	1.10 (0.68-1.80)	**1.80 (1.10-3.00)**	1.00 (0.67-1.50)
0.74 (0.43-1.20)	Pembrolizumab	0.79 (0.46-1.20)	0.83 (0.53-1.20)	1.40 (0.88-2.00)	**0.76 (0.54-0.98)**
0.94 (0.53-1.70)	1.30 (0.81-2.20)	Atezolizumab + platinum-based chemotherapy	1.00 (0.66-1.70)	**1.70 (1.10-2.80)**	0.96 (0.65-1.40)
0.90 (0.56-1.50)	1.20 (0.85-1.90)	0.95 (0.60-1.50)	Ipilimumab + platinum-based chemotherapy	**1.70 (1.20-2.40)**	0.92 (0.72-1.20)
**0.54 (0.33-0.89)**	0.73 (0.51-1.10)	**0.57 (0.36-0.92)**	**0.60 (0.42-0.87)**	Pembrolizumab + platinum-based chemotherapy	**0.55 (0.42-0.72)**
0.98 (0.65-1.50)	**1.30 (1.00-1.90)**	1.00 (0.70-1.50)	1.10 (0.84-1.40)	**1.80 (1.40-2.40)**	Platinum-based chemotherapy

Comparisons should be read clockwise. Hazard ratios were compared between column-defining and row-defining treatments. Numbers in bold font on a darker cell background are statistically significant.

eFigure 13. Network Meta-analysis of Immune Checkpoint Inhibitors in Terms of Progression-Free Survival in Previously Treated Patients

Atezolizumab	1.20 (0.84-1.90)	1.10 (0.66-1.90)	0.95 (0.70-1.30)
0.82 (0.54-1.20)	Nivolumab	0.91 (0.55-1.50)	**0.78 (0.59-0.98)**
0.90 (0.53-1.50)	1.10 (0.68-1.80)	Pembrolizumab	0.85 (0.56-1.30)
1.10 (0.77-1.40)	**1.30 (1.00-1.70)**	1.20 (0.77-1.80)	Docetaxel

Comparisons should be read clockwise. Hazard ratios were compared between column-defining and row-defining treatments. Numbers in bold font on a darker cell background are statistically significant.

© 2019 Yu Y et al. *JAMA Network Open.*

eFigure 14. Network Meta-analysis of Immune Checkpoint Inhibitors in Terms of Overall Survival in Previously Treated Patients

Atezolizumab	1.10 (0.75-1.50)	1.10 (0.69-1.70)	**0.73 (0.56-0.96)**
0.94 (0.66-1.30)	Nivolumab	1.00 (0.67-1.50)	**0.69 (0.55-0.85)**
0.92 (0.59-1.40)	0.97 (0.65, 1.50)	Pembrolizumab	**0.67 (0.47-0.96)**
**1.40 (1.00-1.80)**	**1.50 (1.20, 1.80)**	**1.50 (1.00-2.10)**	Docetaxel

Comparisons should be read clockwise. Hazard ratios were compared between column-defining and row-defining treatments. Numbers in bold font on a darker cell background are statistically significant.

eFigure 15. Pooled Analysis of the Objective Response Rate With Immunotherapy vs Conventional Therapy

© 2019 Yu Y et al. *JAMA Network Open.*

Study or Subgroup	Immunotherapy Events	Immunotherapy Total	Conventional Therapy Events	Conventional Therapy Total	Risk Ratio (95% CI)	Weight, %
**Immune checkpoint inhibitors**						
Lynch et al,[32,a] 2012	53	138	18	66	1.41 (0.90-2.20)	3.3
Lynch et al,[32,b] 2012	53	138	14	66	1.81 (1.09-3.02)	2.9
KEYNOTE-010,[35] 2015	126	390	32	343	3.46 (2.42-4.96)	3.9
KEYNOTE-024,[36] 2016	69	154	42	151	1.61 (1.18-2.20)	4.2
OAK,[37] 2016	58	425	57	425	1.02 (0.72-1.43)	4.0
CheckMate-017,[33,34] 2017	27	135	12	137	2.28 (1.21-4.32)	2.2
CheckMate-057,[33] 2017	56	292	36	290	1.54 (1.05-2.27)	3.7
CheckMate-026,[39] 2017	55	211	57	212	0.97 (0.71-1.33)	4.2
Govindan et al,[40] 2017	172	388	169	361	0.95 (0.81-1.11)	5.4
PACIFIC,[41] 2017	241	473	118	236	1.02 (0.87-1.19)	5.4
CheckMate-078,[42] 2018	57	338	7	166	4.00 (1.87-8.57)	1.8
CheckMate-227,[43] 2018	63	139	43	160	1.69 (1.23-2.31)	4.2
IMpower131,[44] 2018	204	343	174	340	1.16 (1.01-1.33)	5.5
IMpower150,[45] 2018	224	353	159	331	1.32 (1.15-1.52)	5.5
KEYNOTE-021,[46] 2018	34	60	19	63	1.88 (1.21-2.91)	3.3
KEYNOTE-407,[48] 2018	161	278	98	281	1.66 (1.38-2.01)	5.1
Subtotal	1,653	4,255	1,055	3,628	1.47 (1.25-1.73)	64.5
Heterogeneity: $\tau^2 = 0.08$; $\chi^2_{15} = 91.53$ (P <.01); $I^2 = 84\%$						
Test for overall effect: z = 4.70 (P <.01)						
**Tumor vaccine**						
Quoix et al,[20] 2011	31	74	21	74	1.48 (0.94-2.32)	3.2
Alfonso et al,[21] 2014	40	87	56	89	0.73 (0.55-0.96)	4.5
START,[22] 2014	564	829	279	410	1.00 (0.92-1.08)	5.7
Braun et al,[23] 2015	29	48	10	23	1.39 (0.83-2.34)	2.8
TIME,[25] 2015	44	111	32	111	1.38 (0.95-1.99)	3.8
Rodriguez et al,[26] 2016	139	270	64	135	1.09 (0.88-1.34)	5.0
Takayama et al,[27] 2016	6	26	2	24	2.77 (0.62-12.42)	0.6
Katakami et al,[28] 2017	95	114	50	58	0.97 (0.85-1.10)	5.5
Thomas et al,[29] 2017	22	46	6	26	2.07 (0.97-4.45)	1.8
Subtotal	970	1,605	520	950	1.07 (0.93-1.23)	32.9
Heterogeneity: $\tau^2 = 0.02$; $\chi^2_8 = 19.17$ (P =.01); $I^2 = 58\%$						
Test for overall effect: z = 0.99 (P =.32)						
**Cellular immunotherapy**						
Wu et al,[30] 2008	13	29	13	30	1.03 (0.58-1.84)	2.5
Subtotal	13	29	13	30	1.03 (0.58-1.84)	2.5
Heterogeneity: not applicable						
Test for overall effect: z = 0.12 (P =.91)						
Total	2,636	5,889	1,588	4,608	1.33(1.18-1.51)	100.0
Heterogeneity: $\tau^2 = 0.07$; $\chi^2_{25} = 147.06$ (P <.01); $I^2 = 83\%$						
Test for overall effect: z = 4.65 (P <.01)						

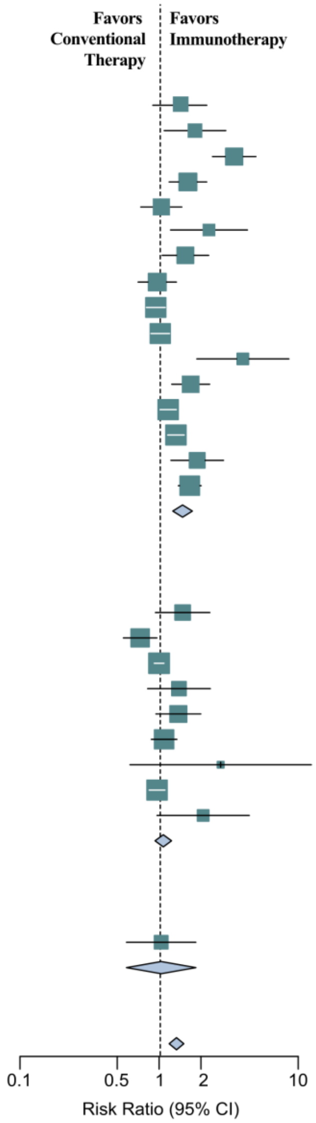

Size of boxes is proportional to the weight of each trial, and the diamonds indicate the point estimate and confidence interval of the combined result.

[a] Response was assessed by using immune-related response.

[b] Response was assessed by using modified WHO criteria.

eFigure 16. Trial Sequential Analyses of Trials Comparing Immunotherapy With Conventional Therapy

© 2019 Yu Y et al. *JAMA Network Open.*

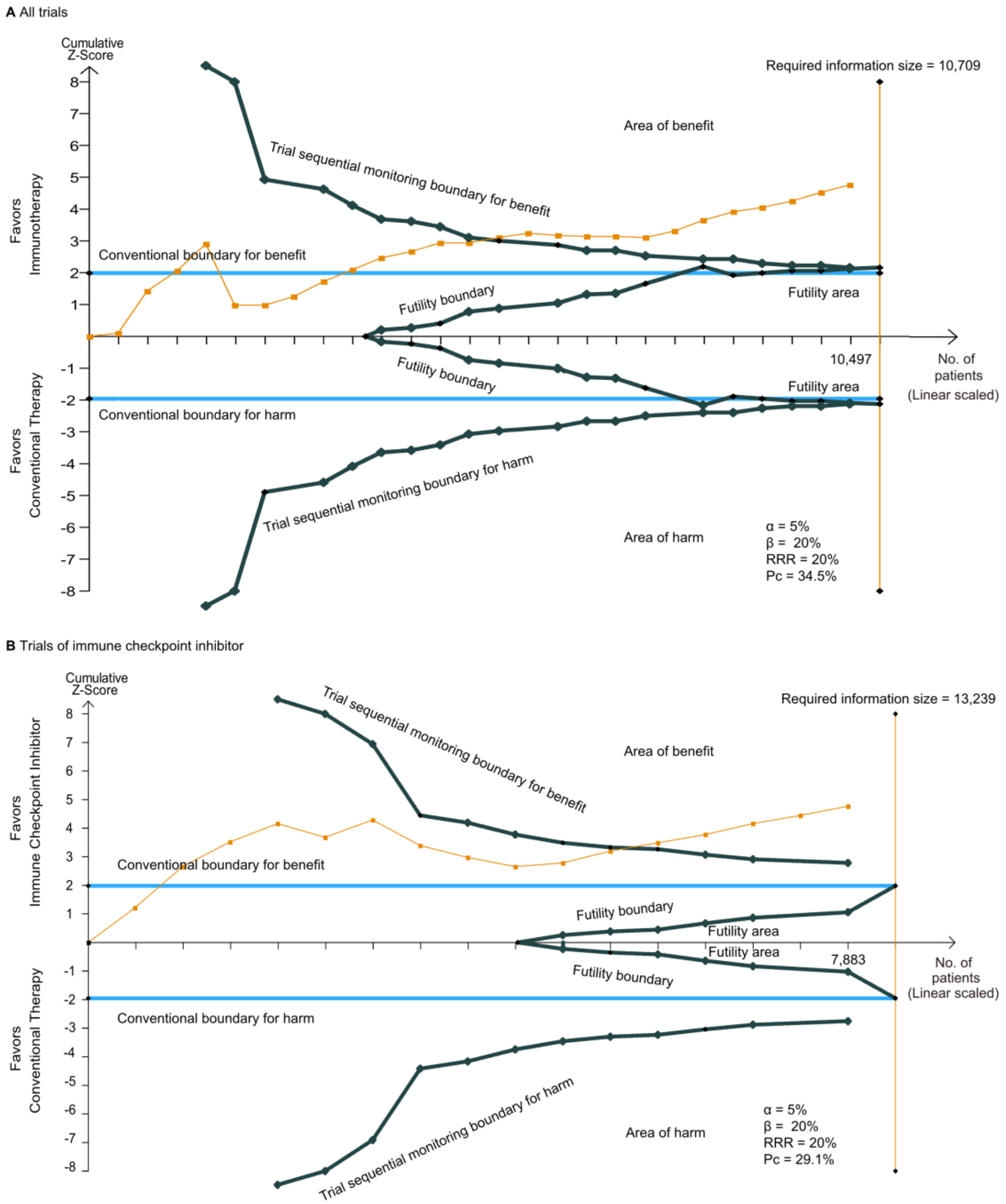

Panel A shows the results of the trial sequential analysis (TSA) for all trials, and panel B shows the results of the TSA for the trials of immune checkpoint inhibitor. α, type I error rate; β, type II error rate; RRR, relative risk reduction; Pc, event proportion of the control group.

eFigure 17. Subgroup Analyses of Progression-Free Survival in Patients Receiving Immune Checkpoint Inhibitor Therapy

© 2019 Yu Y et al. *JAMA Network Open.*

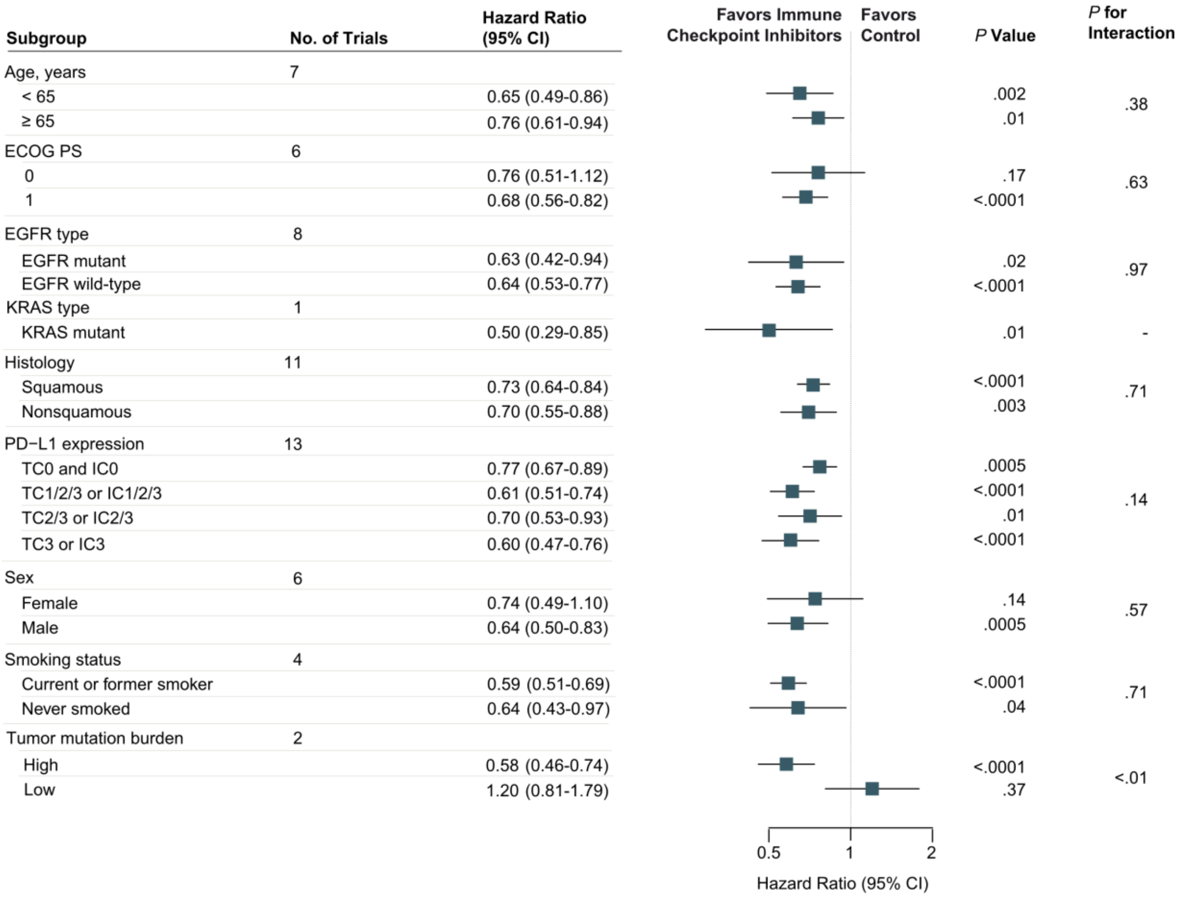

Subgroup	No. of Trials	Hazard Ratio (95% CI)	*P* Value	*P* for Interaction
Age, years	7			
< 65		0.65 (0.49-0.86)	.002	.38
≥ 65		0.76 (0.61-0.94)	.01	
ECOG PS	6			
0		0.76 (0.51-1.12)	.17	.63
1		0.68 (0.56-0.82)	<.0001	
EGFR type	8			
EGFR mutant		0.63 (0.42-0.94)	.02	.97
EGFR wild-type		0.64 (0.53-0.77)	<.0001	
KRAS type	1			
KRAS mutant		0.50 (0.29-0.85)	.01	-
Histology	11			
Squamous		0.73 (0.64-0.84)	<.0001	.71
Nonsquamous		0.70 (0.55-0.88)	.003	
PD−L1 expression	13			
TC0 and IC0		0.77 (0.67-0.89)	.0005	.14
TC1/2/3 or IC1/2/3		0.61 (0.51-0.74)	<.0001	
TC2/3 or IC2/3		0.70 (0.53-0.93)	.01	
TC3 or IC3		0.60 (0.47-0.76)	<.0001	
Sex	6			
Female		0.74 (0.49-1.10)	.14	.57
Male		0.64 (0.50-0.83)	.0005	
Smoking status	4			
Current or former smoker		0.59 (0.51-0.69)	<.0001	.71
Never smoked		0.64 (0.43-0.97)	.04	
Tumor mutation burden	2			
High		0.58 (0.46-0.74)	<.0001	<.01
Low		1.20 (0.81-1.79)	.37	

ECOG PS, Eastern Cooperative Oncology Group Performance Status; EGFR, epithelial growth factor receptor; KRAS, kirsten rat sarcoma viral oncogene homolog; PD-L1, programmed death-ligand 1; TC, tumor cells; IC, tumor-infiltrating immune cells. The *P* value for interaction reflects the difference between subgroups.

PD-L1 scoring criteria:

TC3 or IC3: TC3 ≥50% or IC3 ≥10%.

TC2 or IC2: TC2 ≥5% and <50% or IC2 ≥5% and <10%.

TC1 or IC1: TC1 ≥1% and <5% or IC1 ≥1% and <5%.

TC0 or IC0: TC0 <1% or IC0 <1%.

eFigure 18. Subgroup Analyses of Overall Survival in Patients Receiving Immune Checkpoint Inhibitor Therapy

© 2019 Yu Y et al. *JAMA Network Open.*

Subgroup	No. of Trials	Hazard Ratio (95% CI)	*P* Value	*P* for Interaction
Age, years	8			
< 65		0.73 (0.62-0.86)	.0004	.43
≥ 65		0.80 (0.71-0.91)	.0003	
ECOG PS	8			
0		0.75 (0.63-0.90)	.002	.97
1		0.75 (0.66-0.85)	<.0001	
EGFR type	8			
EGFR mutant		1.12 (0.80-1.56)	.52	<.01
EGFR wild-type		0.68 (0.58-0.79)	<.0001	
KRAS type	4			
KRAS mutant		0.65 (0.44-0.96)	.03	.30
KRAS wild-type		0.81 (0.69-0.95)	.009	
ALK type	1			
ALK wild-type		0.49 (0.38-0.64)	<.0001	-
Histology	10			
Squamous		0.77 (0.69-0.86)	<.0001	.99
Nonsquamous		0.77 (0.67-0.88)	<.0001	
PD−L1 expression	11			
TC0 and IC0		0.72 (0.61-0.86)	.0002	.25
TC1/2/3 or IC1/2/3		0.66 (0.55-0.78)	<.0001	
TC2/3 or IC2/3		0.64 (0.51-0.81)	.0002	
TC3 or IC3		0.56 (0.47-0.67)	<.0001	
Sex	8			
Female		0.71 (0.54-0.94)	.02	.56
Male		0.78 (0.72-0.85)	<.0001	
Smoking status	7			
Current or former smoker		0.79 (0.69-0.90)	.0004	.79
Never smoked		0.75 (0.53-1.06)	.1	

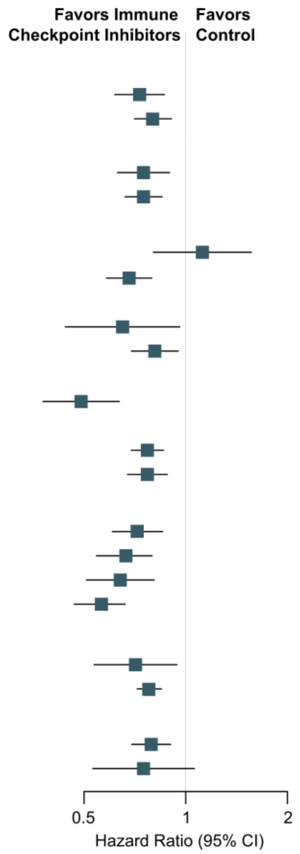

ECOG PS, Eastern Cooperative Oncology Group Performance Status; EGFR, epithelial growth factor receptor; KRAS, kirsten rat sarcoma viral oncogene homolog; ALK, anaplastic lymphoma kinase; PD-L1, programmed death-ligand 1; TC, tumor cells; IC, tumor-infiltrating immune cells. The *P* value for interaction reflects the difference between subgroups.

PD-L1 scoring criteria:

TC3 or IC3: TC3 ≥50% or IC3 ≥10%.

TC2 or IC2: TC2 ≥5% and <50% or IC2 ≥5% and <10%.

TC1 or IC1: TC1 ≥1% and <5% or IC1 ≥1% and <5%.

TC0 or IC0: TC0 <1% or IC0 <1%.

eFigure 19. Response and Clinical Benefit to Checkpoint Inhibitor Relative to Molecular Features in Cohort 1

© 2019 Yu Y et al. *JAMA Network Open.*

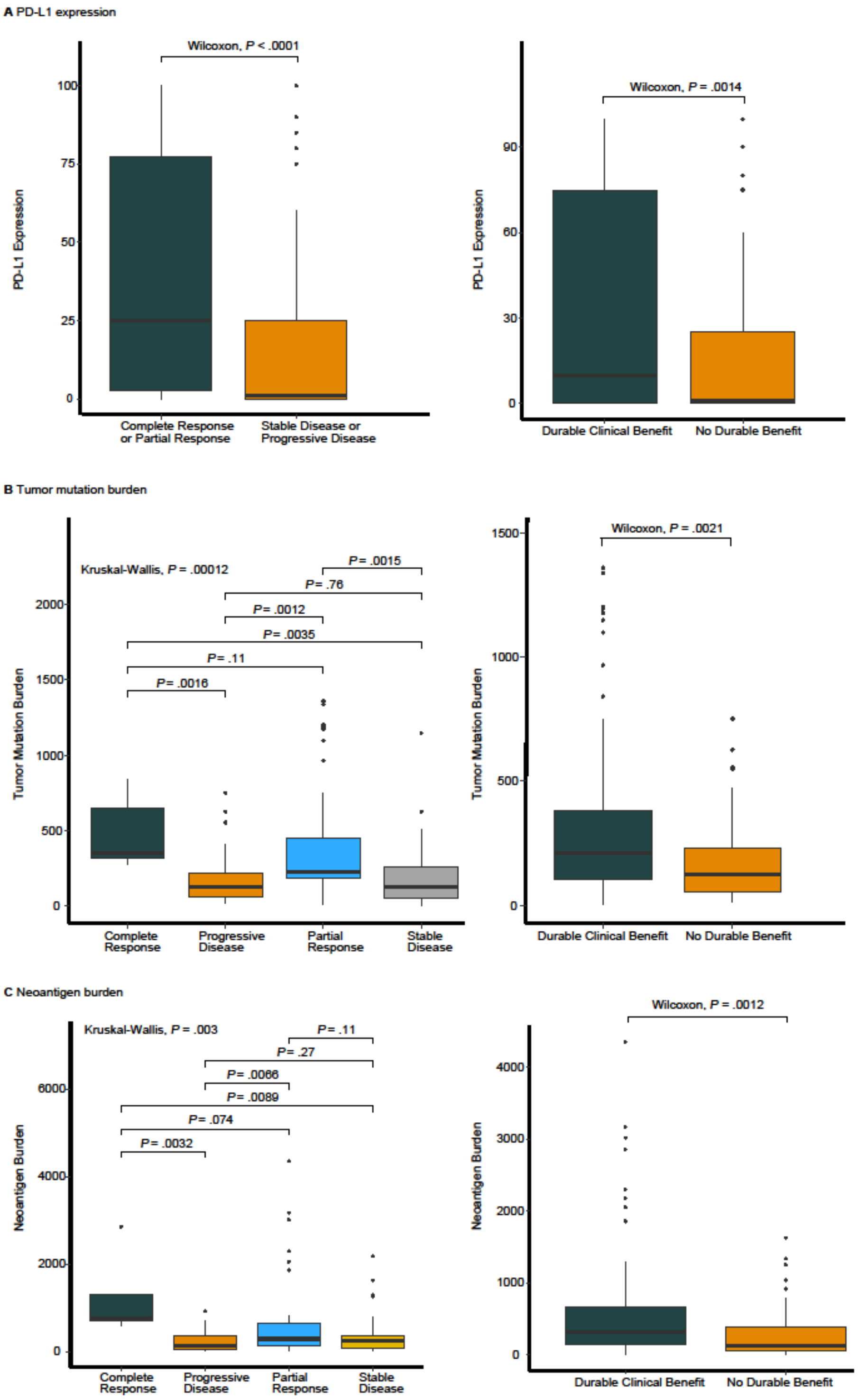

The PD-L1 expression, tumor mutation burden (TMB) and neoantigen burden (NAB) in patients with a complete response, a partial response, stable disease or progressive disease are shown in the left graphs in the Panels A, B and C, respectively. The PD-L1 expression,

© 2019 Yu Y et al. *JAMA Network Open.*

TMB and NAB in patients with durable clinical benefit versus those with no durable clinical benefit are shown in the right graphs in the Panels A, B and C, respectively.

eFigure 20. Progression-Free Survival Analysis Stratified by Neoantigen Burden in Cohort 1

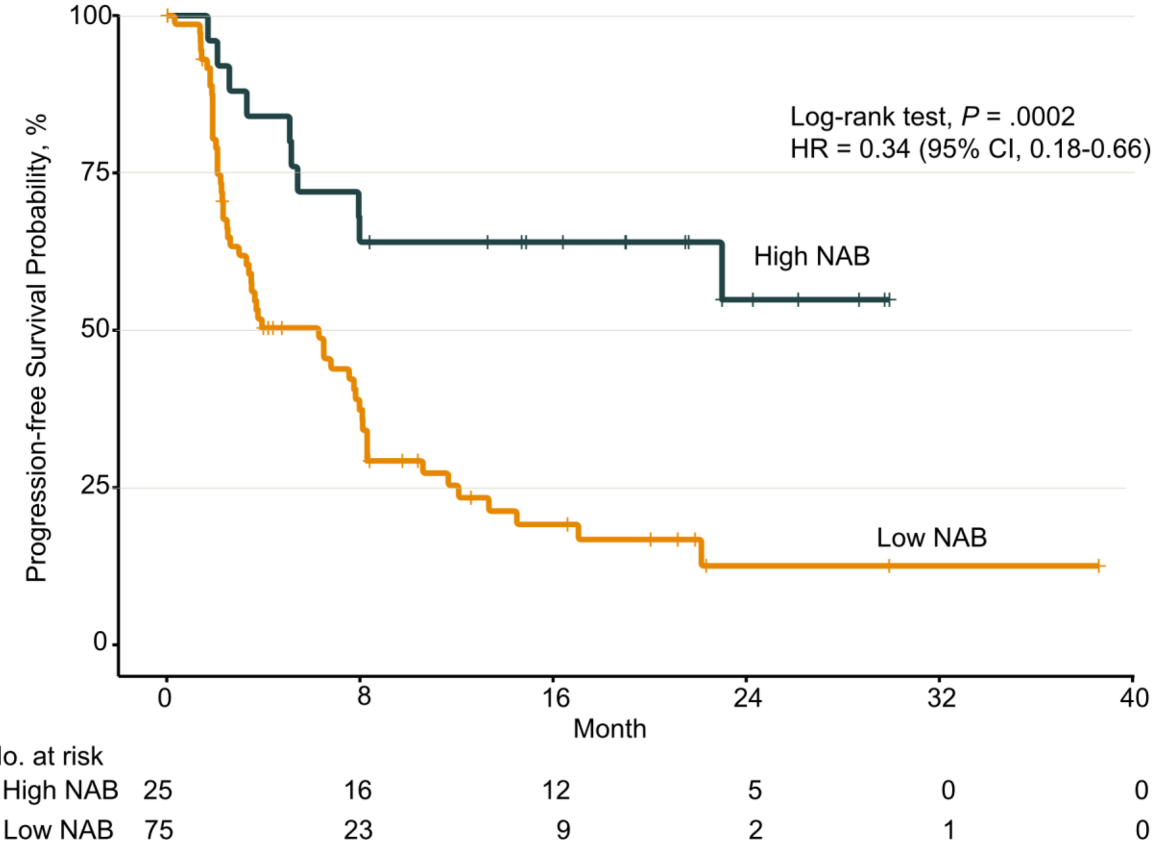

HR, hazard ratio; NAB, neoantigen burden.

eFigure 21. Overall Survival Analysis Stratified by Molecular Features in the TCGA Cohort

© 2019 Yu Y et al. *JAMA Network Open.*

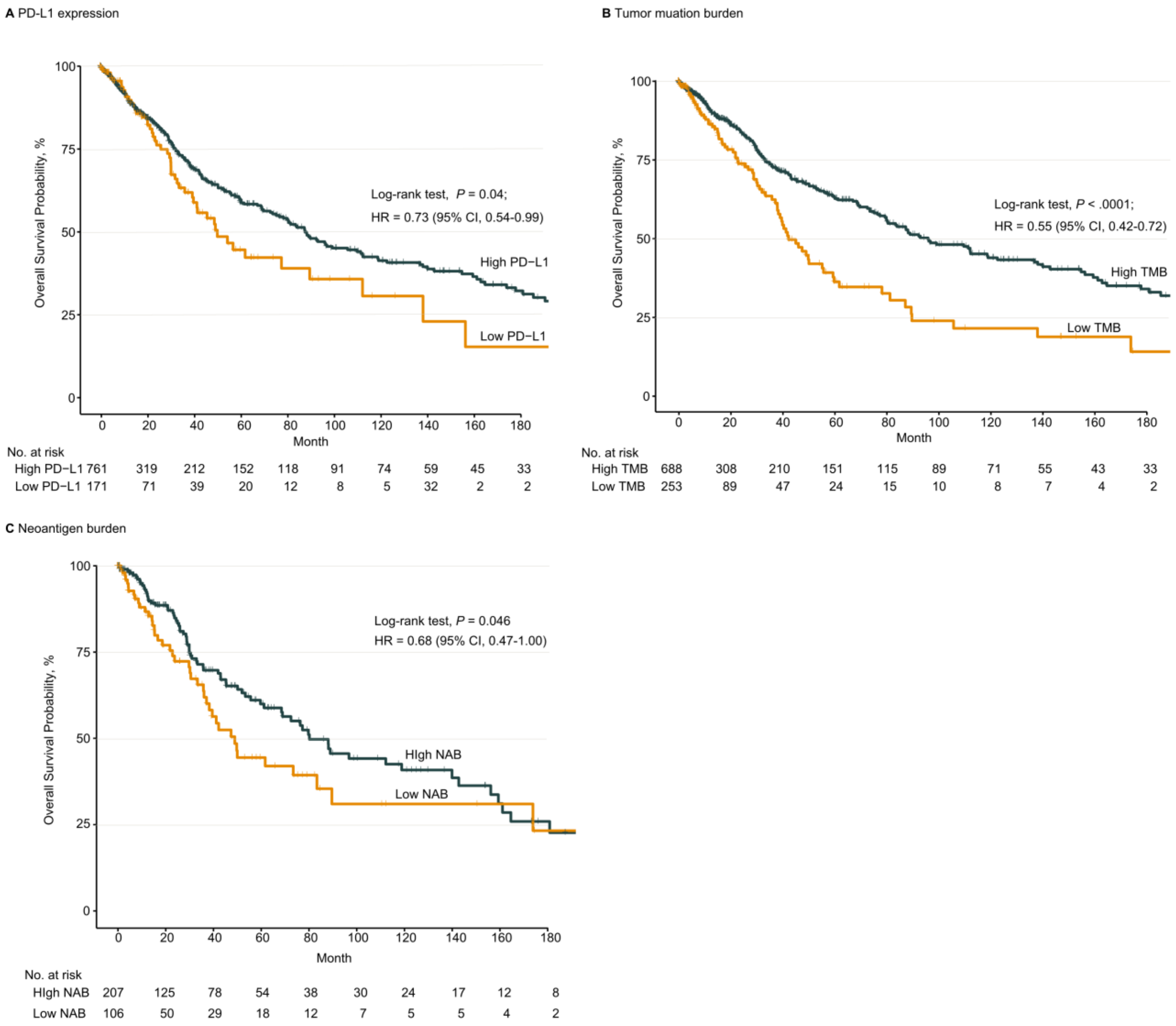

Overall survival curves of high PD-L1 expression versus low PD-L1 expression, high tumor mutation burden versus low tumor mutation burden and high neoantigen burden versus low neoantigen burden are shown in Panels A, B and C, respectively. TCGA, Cancer Genome Atlas; HR, hazard ratio; PD-L1, programmed death-ligand 1; TMB, tumor mutation burden; NAB, neoantigen burden.

eFigure 22. Receiver Operating Characteristic Curves Correlating Molecular Features With Clinical Outcomes in Cohort 1

© 2019 Yu Y et al. *JAMA Network Open.*

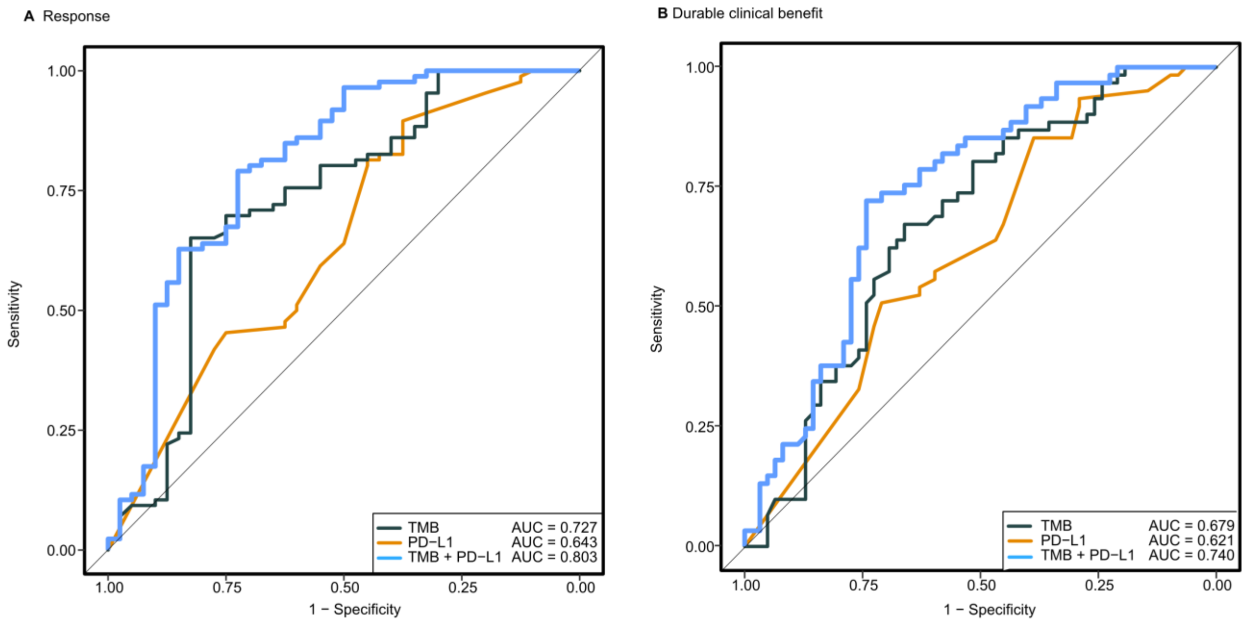

Panel A shows the receiver operating characteristic (ROC) curves for the correlation of tumor mutation burden/PD-L1 expression with complete response/partial response; Panel B shows the ROC curves for the correlation of tumor mutation burden/PD-L1 expression with durable clinical benefit. AUC, the area under the curve; PD-L1, programmed death-ligand 1; TMB, tumor mutation burden.

eFigure 23. Scatterplots of Molecular Features in Cohort 1

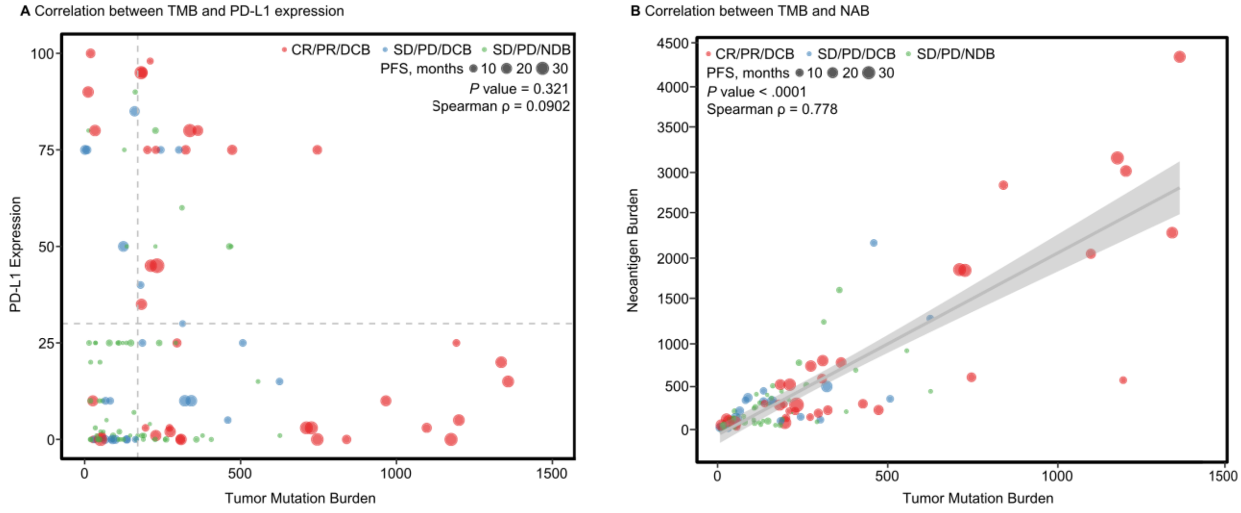

Panel A shows the correlation between the tumor mutation burden and expression of PD-L1; Panel B shows the correlation between tumor mutation burden and neoantigen burden. PD-L1, programmed death-ligand 1; TMB, tumor mutation burden; NAB, neoantigen burden; PFS, progression-free survival; CR, complete response; PR, partial response; SD, stable disease; PD, progressive disease; DCB, durable clinical benefit; NDB, no durable benefit.

© 2019 Yu Y et al. *JAMA Network Open.*

eFigure 24. Progression-Free Survival Analysis Stratified by Programmed Cell Death Ligand 1 Expression and Tumor Mutation Burden in Cohort 1

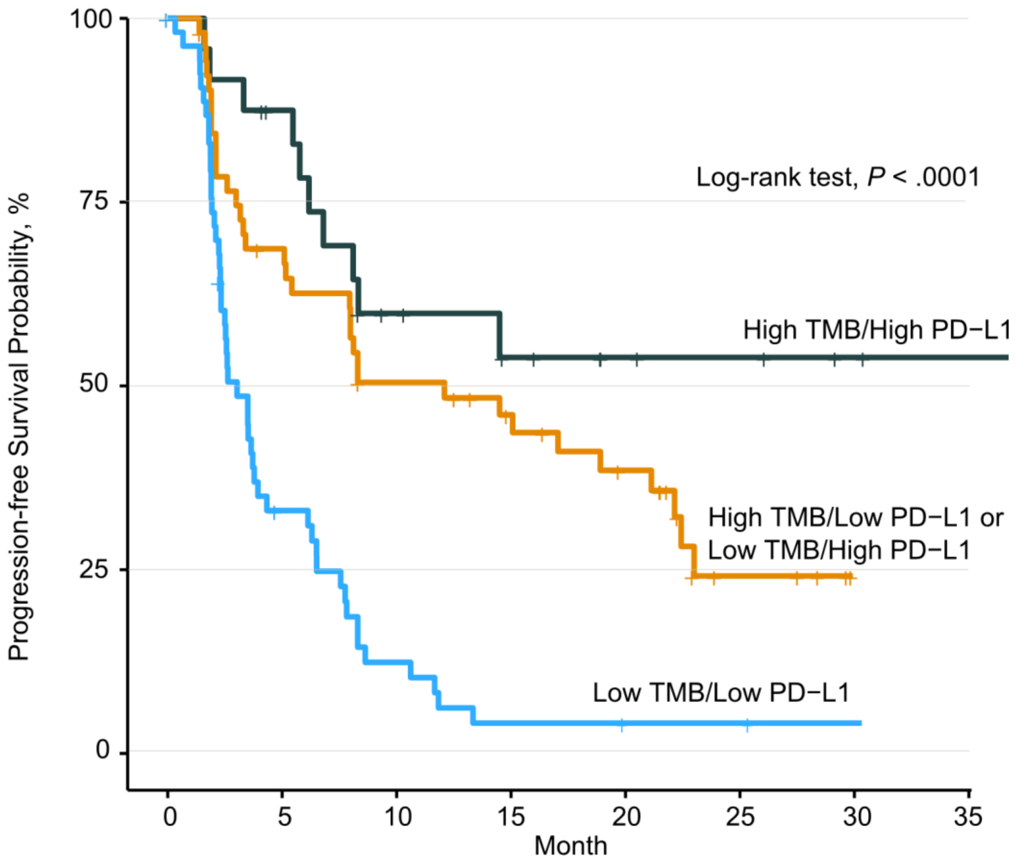

No. at risk

	0	5	10	15	20	25	30	35
High TMB/High PD−L1	24	19	11	8	5	4	3	1
High TMB/Low PD−L1 or Low TMB/High PD−L1	54	34	24	19	14	4	0	0
Low TMB/Low PD−L1	54	16	6	2	2	1	1	0

PD-L1, programmed death-ligand 1; TMB, tumor mutation burden.

eFigure 25. Response and Clinical Benefit to Checkpoint Inhibitor Stratified by Programmed Cell Death Ligand 1 and Tumor Mutation Burden

© 2019 Yu Y et al. *JAMA Network Open.*

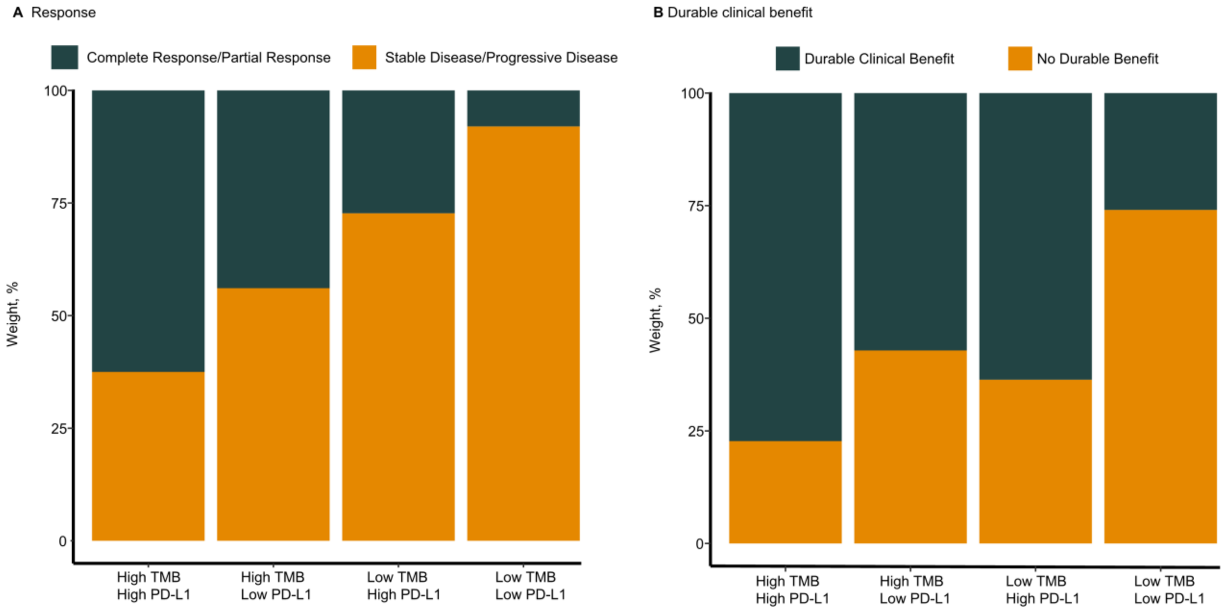

Panel A shows the complete response/partial response and stable disease/progressive disease rates in patients with different combinations of the tumor mutation burden and expression of PD-L1; Panel B shows the durable clinical benefit/no durable benefit rates in patients with different combinations of the tumor mutation burden and expression of PD-L1; TMB, tumor mutation burden; PD-L1, programmed death-ligand 1.

eFigure 26. Receiver Operating Characteristic Curves Correlating Molecular Features With Survival in the Cancer Genome Atlas Cohort

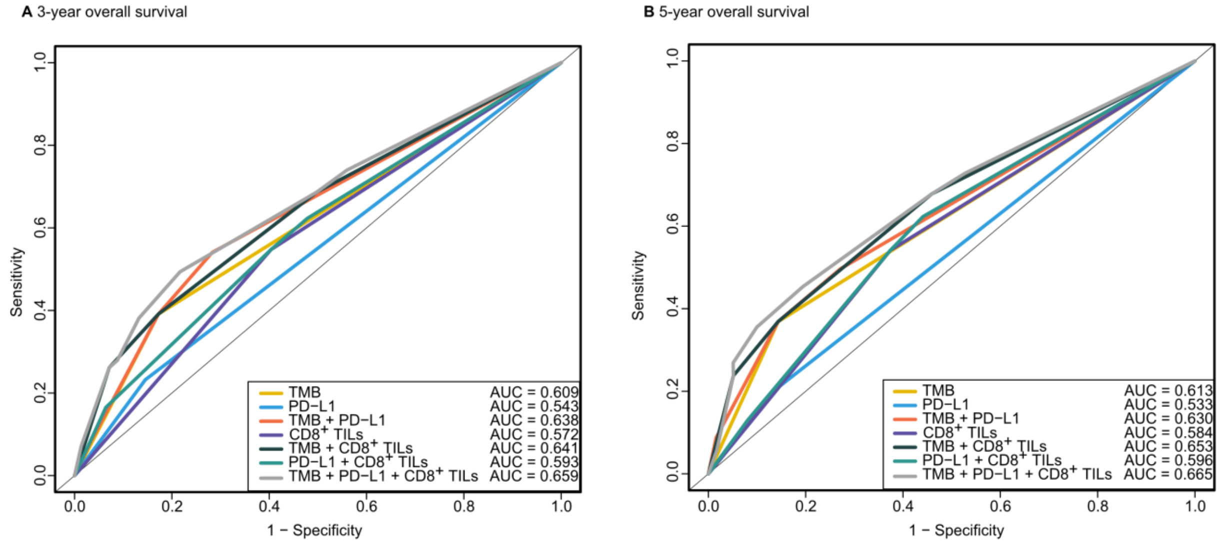

A, Receiver operating characteristic curves correlating multiple molecular features with 3-year overall survival. B, Same as A but were plotted for 5-year overall survival. Abbreviations: PD-L1,

© 2019 Yu Y et al. *JAMA Network Open.*

programmed death-ligand 1; TMB, tumor mutation burden; TILs, tumor-infiltrating lymphocytes; TCGA, The Cancer Genome Atlas.

eFigure 27. Unsupervised Consensus Clustering of Immune Subtypes in the Cancer Genome Atlas Cohort

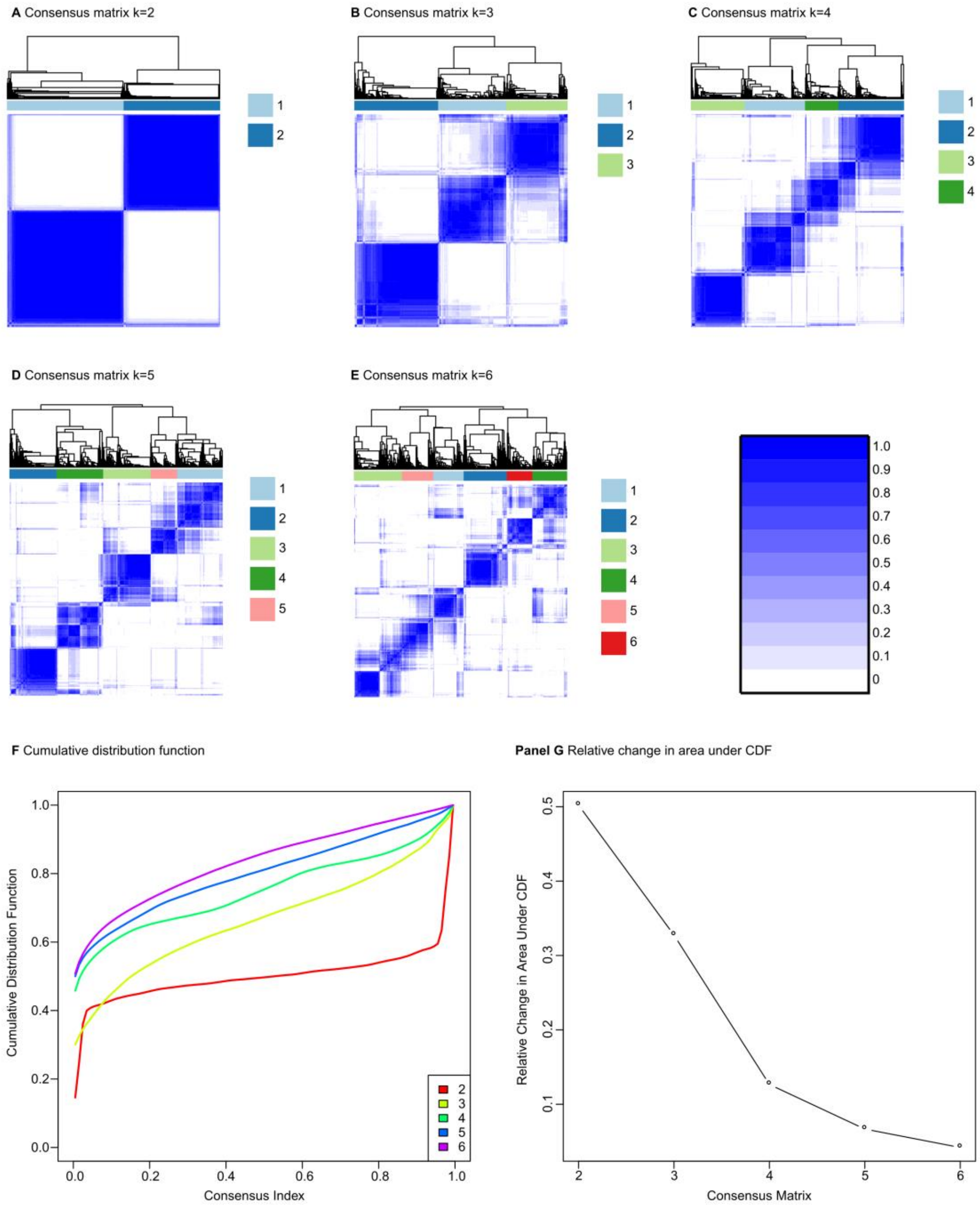

Panels A to E show the consensus matrices represented as heatmaps for the chosen cluster numbers (k = 2 to 6); Panel F shows the cumulative distribution function curve for the chosen cluster

© 2019 Yu Y et al. *JAMA Network Open.*

numbers (k = 2 to 6); Panel G shows the corresponding relative change in the area under the cumulative distribution function curve when the cluster number changes from 2 to 6. CDF, cumulative distribution function curve; TCGA, The Cancer Genome Atlas.

eFigure 28. Molecular Features and Survival Stratified by Immune Subtype in the Cancer Genome Atlas Cohort

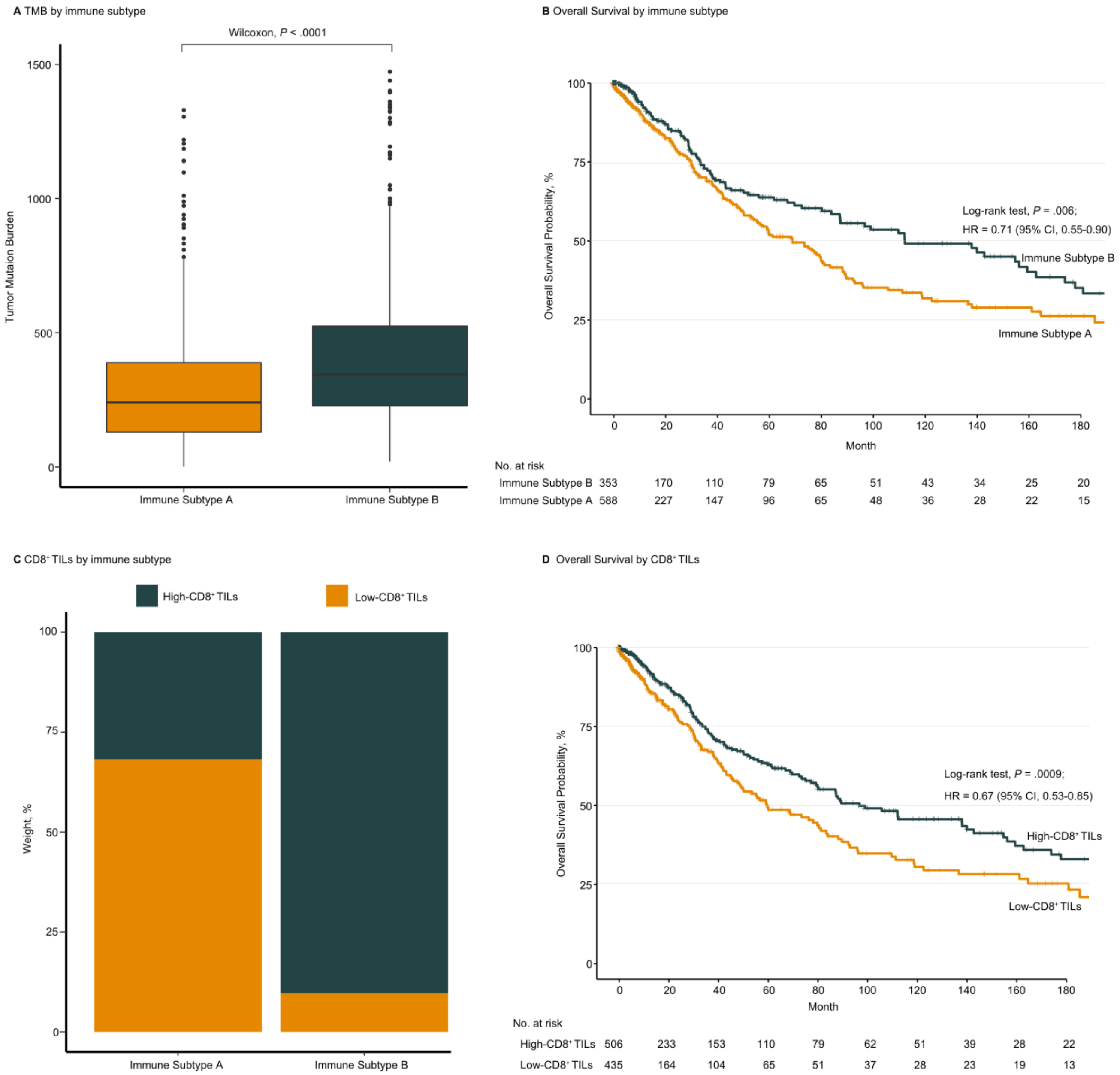

Panel A shows the tumor mutation burden in the patients with the immune subtype A tumors versus those with immune subtype B tumors. Panel B shows the overall survival curve of the

© 2019 Yu Y et al. *JAMA Network Open.*

immune subtype A group versus that of the immune subtype B group. Panel C shows the proportions of high- and low- $CD8^+$ tumor-infiltrating lymphocytes (TILs) in patients with immune subtype A versus those with immune subtype B. Panel D shows the overall survival curve of the high-$CD8^+$ TILs group versus that of the low-$CD8^+$ TILs group. TMB, tumor mutation burden; HR, hazard ratio; TCGA, The Cancer Genome Atlas; TILs, tumor-infiltrating lymphocytes.

eFigure 29. Identification of the Most Important Immune Feature Using Random Forest Method

© 2019 Yu Y et al. *JAMA Network Open.*

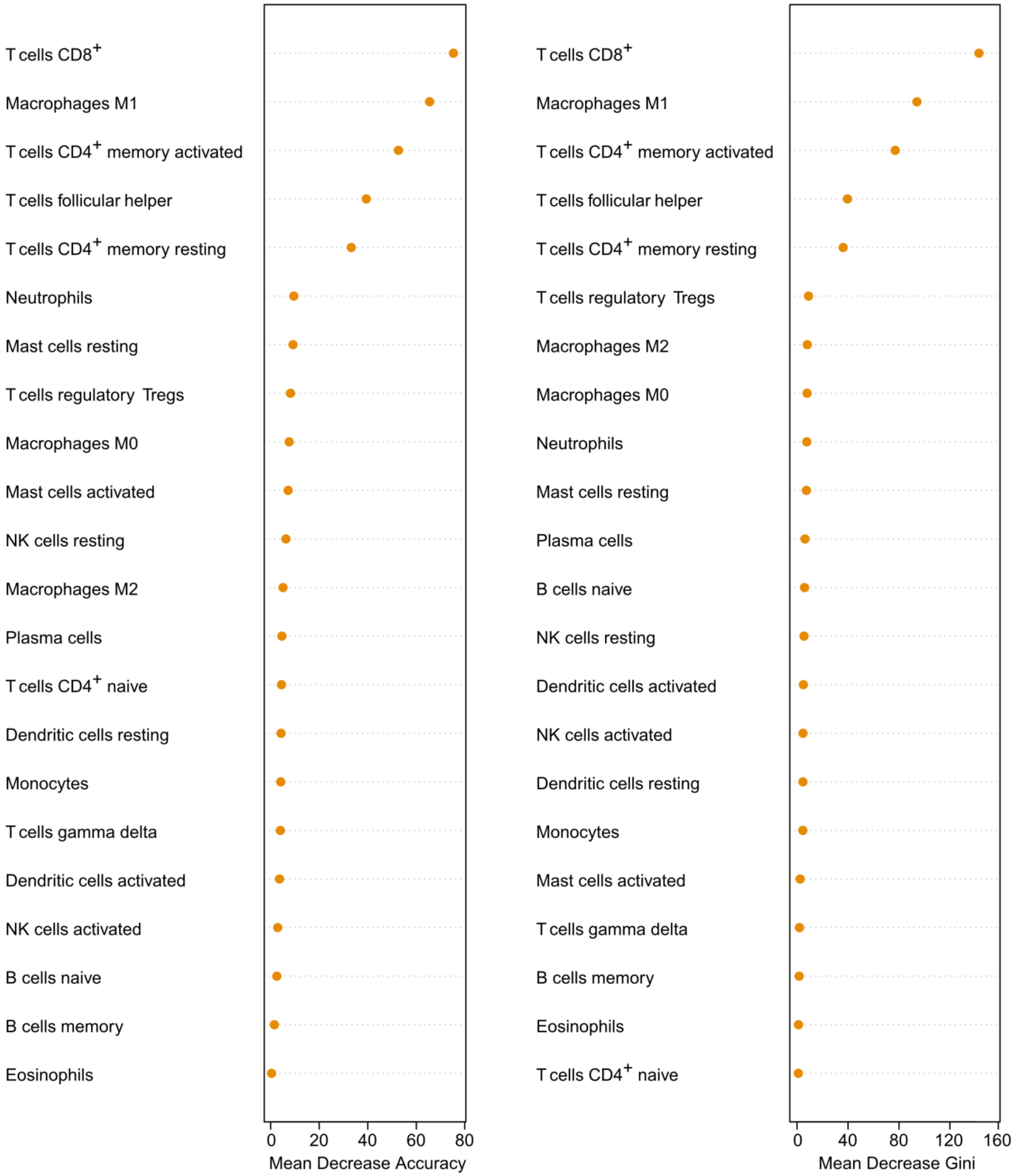

Abbreviations: NK, natural killer.

© 2019 Yu Y et al. *JAMA Network Open.*

eFigure 30. Scatterplots of Molecular Features in the Cancer Genome Atlas Cohort

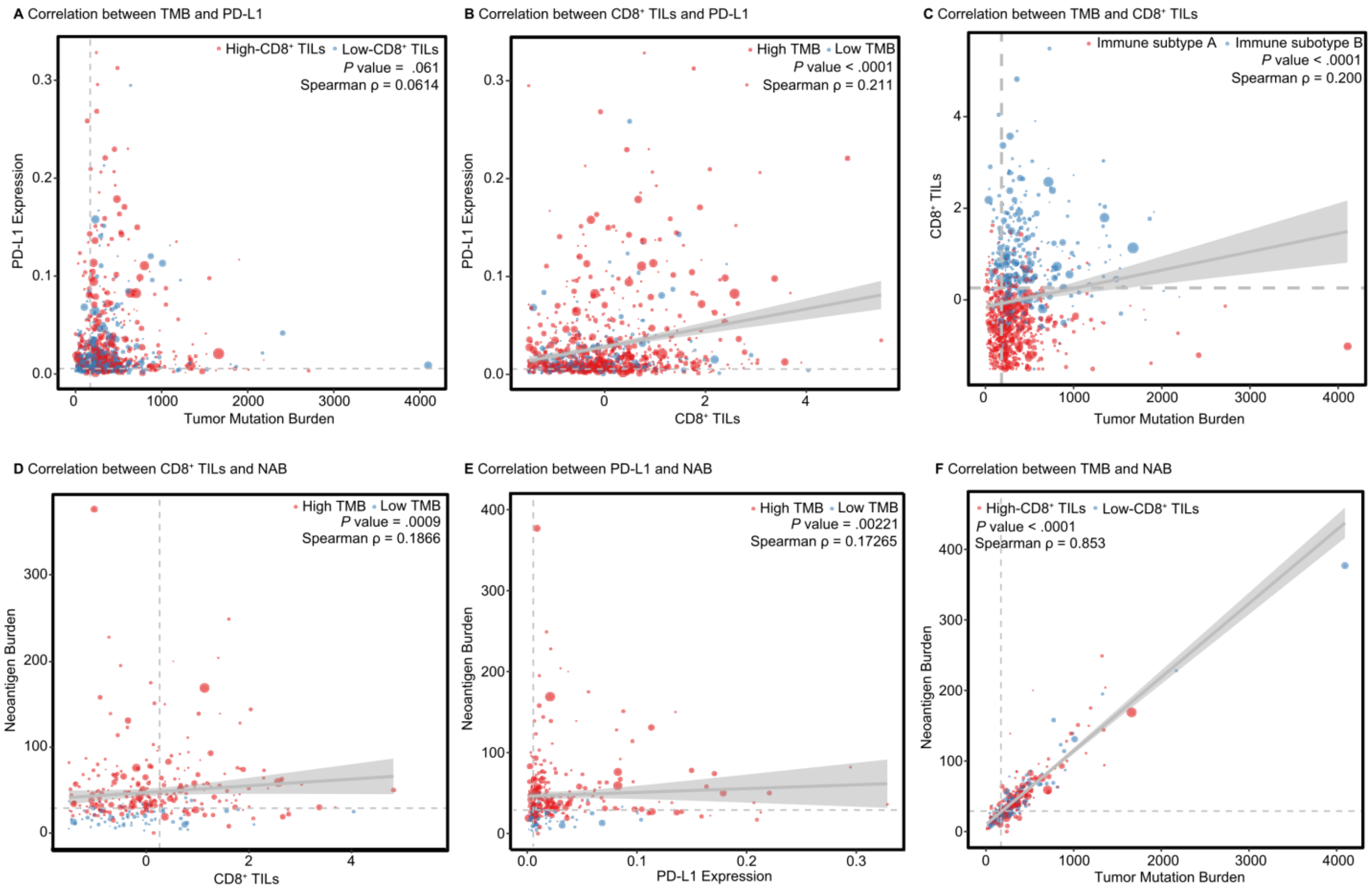

Panel A shows the correlation between the tumor mutation burden (TMB) and expression of PD-L1; Panel B shows the correlation between CD8$^+$ tumor-infiltrating lymphocytes (TILs) and PD-L1 expression; Panel C shows the correlation between the TMB and CD8$^+$ TILs. Panel D shows the correlation between CD8$^+$ TILs and the neoantigen burden (NAB); Panel E shows the correlation between the NAB and expression of PD-L1; and Panel F shows the correlation between the TMB and NAB. PD-L1, programmed death-ligand 1; TMB, tumor mutation burden; NAB, neoantigen burden; TILs, tumor-infiltrating lymphocytes; TCGA, The Cancer Genome Atlas

© 2019 Yu Y et al. *JAMA Network Open.*

eFigure 31. Individual Gene Alterations Associated With Checkpoint Blockade Efficacy and Molecular Features

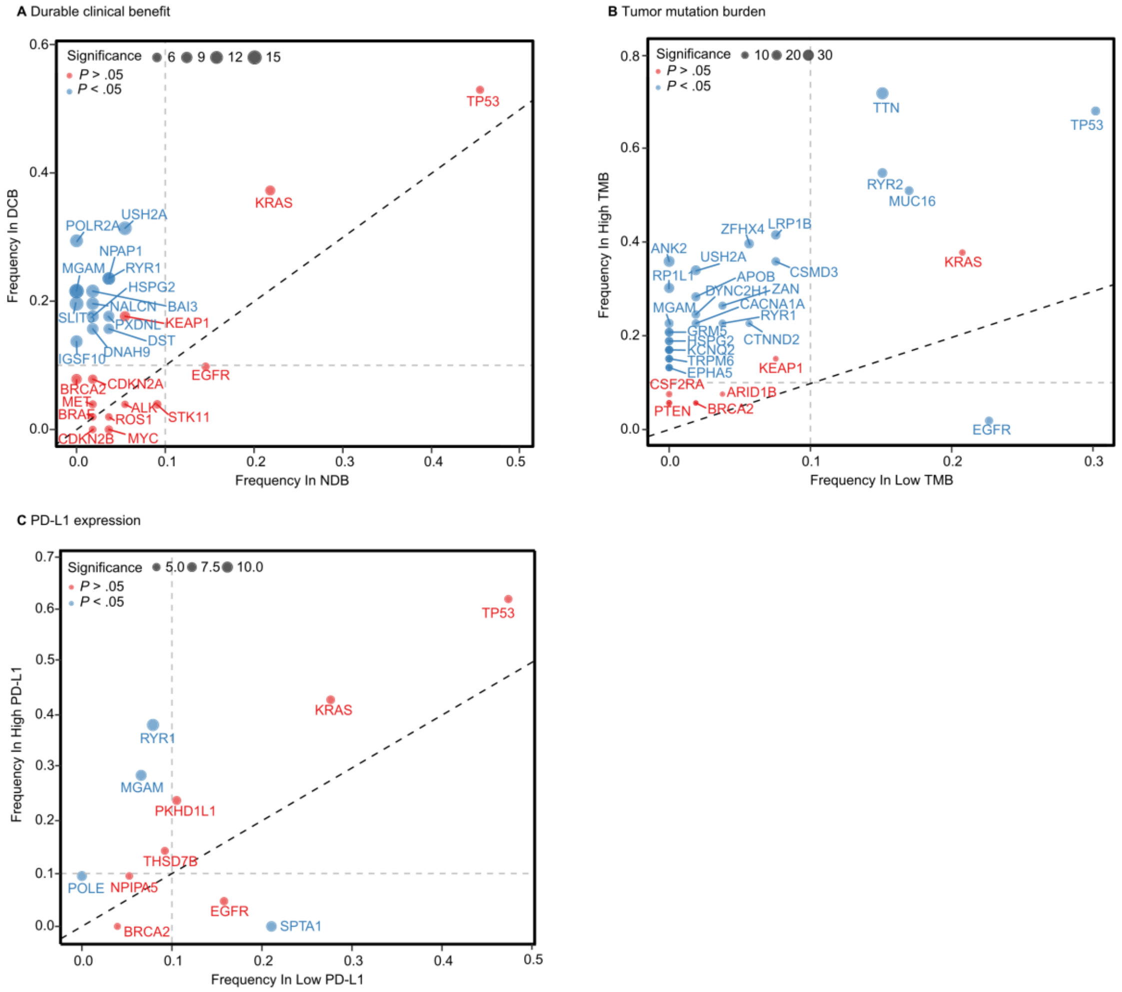

A, The frequencies of the altered genes in patients with durable clinical benefit versus those with no durable benefit. B, Same as B but comparing the high tumor mutation burden group versus the low tumor mutation burden group. C, Same as B but comparing the high PD-L1 group versus the low PD-L1 group. Abbreviations: DCB, durable clinical benefit; NDB, no durable benefit; PD-L1, programmed death-ligand 1; TMB, tumor mutation burden.

© 2019 Yu Y et al. *JAMA Network Open.*

eReferences.

1. Higgins JPT, Green S. *Cochrane Handbook for Systematic Reviews of Interventions* Version 5.1.0 [updated March 2011]. The Cochrane Collaboration, 2011. Available from http://handbook.cochrane.org. (accessed April 14, 2017).
2. Eisenhauer EA, Therasse P, Bogaerts J, et al. New response evaluation criteria in solid tumours: revised RECIST guideline (version 1.1). *Eur J Cancer*. 2009;45(2):228–47.
3. Dias S, Sutton AJ, Ades AE, et al. Evidence synthesis for decision making 2: a generalized linear modeling framework for pairwise and network meta-analysis of randomized controlled trials. *Med Decis Making*. 2013;33(5):607–617.
4. Wetterslev J, Thorlund K, Brok J, Gluud C. Trial sequential analysis may establish when firm evidence is reached in cumulative meta-analysis. *J Clin Epidemiol*. 2008;61(1):64-75.
5. Brok J, Thorlund K, Gluud C, Wetterslev J. Trial sequential analysis reveals insufficient information size and potentially false positive results in many meta-analyses. *J Clin Epidemiol*. 2008;61(8):763-9.
6. Brok J, Thorlund K, Wetterslev J, Gluud C. Apparently conclusive meta-analysis may be inconclusive—trial sequential analysis adjustment of random error risk due to repetitive testing of accumulating data in apparently conclusive neonatal meta-analysis. *Int J Epidemiol*. 2009;38(1):287-98.
7. Thorlund K, Devereaux PJ, Wetterslev J, et al. Can trial sequential monitoring boundaries reduce spurious inferences from meta-analysis? *Int J Epidemiol*. 2009;38(1):276-86.
8. Copas JB, Shi JQ. A sensitivity analysis for publication bias in systematic reviews. *Stat Methods Med Res*. 2001;10(4):251–265.
9. Begg CB, Mazumdar M. Operating characteristics of a rank correlation test for publication bias. *Biometrics*. 1994;50(4):1088-101.
10. Egger M, Davey SG, Schneider M, Minder C. Bias in meta-analysis detected by a simple, graphical test. *BMJ*. 1997;315(7109):629-634.
11. GRADE working group website. http://www.gradeworkinggroup.org/. Accessed March 14, 2017.
12. Guyatt GH, Oxman AD, Vist GE, et al. GRADE Working Group. GRADE: an emerging consensus on rating quality of evidence and strength of recommendations. *BMJ*. 2008;336(7650):924-926.
13. Pak K, Uno H, Kim DH, et al. Interpretability of Cancer Clinical Trial Results Using Restricted Mean Survival Time as an Alternative to the Hazard Ratio. *JAMA Oncol.* 2017;3(12):1692-1696.
14. Kursa MB, Rudnicki WR. Feature Selection with the Boruta Package. *Journal of Statistical Sof*tware. 2010;36(11).
15. Lawrence MS, Stojanov P, Polak P, et al. Mutational heterogeneity in cancer and the search for new cancer genes. *Nature*. 2013; 499(7457):214-218.

© 2019 Yu Y et al. *JAMA Network Open.*

16. Newman AM, Liu CL, Green MR, et al. Robust enumeration of cell subsets from tissue expression profiles. *Nat Methods*. 2015;12(5):453-457
17. Hartigan JA, Wong MA. Algorithm AS 136: A k-means clustering algorithm. *Appl Stat*. 1979;28(1): 100-108.
18. Monti S, Tamayo P, Mesirov J, Golub T. Consensus Clustering: A Resampling-Based Method for Class Discovery and Visualization of Gene Expression Microarray Data. *Mach Learn*. 2003;52(1-2):91-118.
19. Butts C, Murray N, Maksymiuk A, et al. Randomized phase IIB trial of BLP25 liposome vaccine in stage IIIB and IV non-small-cell lung cancer. *J Clin Oncol.* 2005;23(27):6674-6681.
20. Quoix E, Ramlau R, Westeel V, et al. Therapeutic vaccination with TG4010 and first-line chemotherapy in advanced non-small-cell lung cancer: a controlled phase 2B trial. *Lancet Oncol.* 2011;12(12):1125-1133.
21. Alfonso S, Valdes-Zayas A, Santiesteban ER, et al. A randomized, multicenter, placebo-controlled clinical trial of racotumomab-alum vaccine as switch maintenance therapy in advanced non-small cell lung cancer patients. *Clin Cancer Res.* 2014;20(14):3660-3671.
22. Butts C, Socinski MA, Mitchell PL, et al. Tecemotide (L-BLP25) versus placebo after chemoradiotherapy for stage III non-small-cell lung cancer (START): a randomised, double-blind, phase 3 trial. *Lancet Oncol.* 2014;15(1):59-68.
23. Braun A, Engel-Riedel W, Schneller F, et al. 112pefficacy and safety of imprime PGG, a novel innate immune modulator, in combination with bevacizumab (BEV), carboplatin and paclitaxel for the 1st-line treatment of stage IV NSCLC. *Ann Oncol.* 2015;26(suppl_1):i35.
24. Giaccone G, Bazhenova LA, Nemunaitis J, et al. A phase III study of belagenpumatucel-L, an allogeneic tumour cell vaccine, as maintenance therapy for non-small cell lung cancer. *Eur J Cancer.* 2015;51(16):2321-2329.
25. Quoix E, Lena H, Losonczy G, et al. TG4010 immunotherapy and first-line chemotherapy for advanced non-small-cell lung cancer (TIME): results from the phase 2b part of a randomised, double-blind, placebo-controlled, phase 2b/3 trial. *Lancet Oncol.* 2016;17(2):212-223.
26. Rodriguez PC, Popa X, Martinez O, et al. A phase III clinical trial of the epidermal growth factor vaccine CIMAvax-EGF as switch maintenance therapy in advanced non-small cell lung cancer patients. *Clin Cancer Res.* 2016;22(15):3782-3790.
27. Takayama K, Sugawara S, Saijo Y, et al. Randomized phase II study of docetaxel plus personalized peptide vaccination versus docetaxel plus placebo for patients with previously treated advanced wild type EGFR non-small-cell lung cancer. *J Immunol Res.* 2016;2016:1745108.
28. Katakami N, Hida T, Nokihara H, et al. Phase I/II study of tecemotide as immunotherapy in Japanese patients with unresectable stage III non-small cell lung cancer. *Lung Cancer.* 2017;105:23-30.

© 2019 Yu Y et al. *JAMA Network Open.*

29. Thomas M, Sadjadian P, Kollmeier J, et al. A randomized, open-label, multicenter, phase II study evaluating the efficacy and safety of BTH1677 (1,3-1,6 beta glucan; Imprime PGG) in combination with cetuximab and chemotherapy in patients with advanced non-small cell lung cancer. *Invest New Drugs.* 2017;35(3):345-358.
30. Wu C, Jiang J, Shi L, Xu N. Prospective study of chemotherapy in combination with cytokine-induced killer cells in patients suffering from advanced non-small cell lung cancer. *Anticancer Res.* 2008;28(6b):3997-4002.
31. Li R, Wang C, Liu L, et al. Autologous cytokine-induced killer cell immunotherapy in lung cancer: a phase II clinical study. *Cancer Immunol Immunother.* 2012;61(11):2125-2133.
32. Lynch TJ, Bondarenko I, Luft A, et al. Ipilimumab in combination with paclitaxel and carboplatin as first-line treatment in stage IIIB/IV non-small-cell lung cancer: results from a randomized, double-blind, multicenter phase II study. *J Clin Oncol.* 2012;30(17):2046-2054.
33. Horn L, Spigel DR, Vokes EE, et al. Nivolumab versus docetaxel in previously treated patients with advanced non-small-cell lung cancer: two-year outcomes from two randomized, open-label, phase III trials (checkmate 017 and checkmate 057). *J Clin Oncol.* 2017;35(35):3924-3933.
34. Brahmer J, Reckamp KL, Baas P, et al. Nivolumab versus docetaxel in advanced squamous-cell non-small-cell lung cancer. *N Engl J Med.* 2015;373(2):123-135.
35. Herbst RS, Baas P, Kim DW, et al. Pembrolizumab versus docetaxel for previously treated, PD-L1-positive, advanced non-small-cell lung cancer (KEYNOTE-010): a randomised controlled trial. *Lancet.* 2016;387(10027):1540-1550.
36. Reck M, Rodriguez-Abreu D, Robinson AG, et al. Pembrolizumab versus chemotherapy for PD-L1-positive non-small-cell lung cancer. *N Engl J Med.* 2016;375(19):1823-1833.
37. Rittmeyer A, Barlesi F, Waterkamp D, et al. Atezolizumab versus docetaxel in patients with previously treated non-small-cell lung cancer (OAK): a phase 3, open-label, multicentre randomised controlled trial. *Lancet.* 2016;389(10066):255-265.
38. Fehrenbacher L, Spira A, Ballinger M, et al. Atezolizumab versus docetaxel for patients with previously treated non-small-cell lung cancer (POPLAR): a multicentre, open-label, phase 2 randomised controlled trial. *Lancet.* 2016;387(10030):1837-1846.
39. Carbone DP, Reck M, Paz-Ares L, et al. First-line nivolumab in stage IV or recurrent non-small-cell lung cancer. *N Engl J Med.* 2017;376(25):2415-2426.
40. Govindan R, Szczesna A, Ahn MJ, et al. Phase III trial of ipilimumab combined with paclitaxel and carboplatin in advanced squamous non-small-cell lung cancer. *J Clin Oncol.* 2017;35(30):3449-3457.
41. Antonia SJ, Villegas A, Daniel D, et al. Durvalumab after chemoradiotherapy in stage III non-small cell lung cancer. *N Engl J Med.* 2017;377(20):1919-1929.
42. Wu Y-L, Lu S, Cheng Y, et al. Abstract CT114: nivolumab versus docetaxel in a predominantly Chinese patient population with previously treated advanced non-small

cell lung cancer (NSCLC): results of the phase 3 CheckMate 078 study. *Cancer Res*. 2018;78(13 Supplement):CT114.
43. Hellmann MD, Ciuleanu TE, Pluzanski A, et al. Nivolumab plus ipilimumab in lung cancer with a high tumor mutational burden. *N Engl J Med.* 2018;378(22):2093-2104.
44. Jotte RM, Cappuzzo F, Vynnychenko I, et al. IMpower131: primary PFS and safety analysis of a randomized phase III study of atezolizumab + carboplatin + paclitaxel or nab-paclitaxel vs carboplatin + nab-paclitaxel as 1L therapy in advanced squamous NSCLC. *J Clin Oncol.* 2018;36(18_suppl):LBA9000.
45. Socinski MA, Jotte RM, Cappuzzo F, et al. Atezolizumab for first-line treatment of metastatic nonsquamous NSCLC. *N Engl J Med.* 2018;378(24):2288-2301.
46. Langer CJ, Gadgeel SM, Borghaei H, et al. Carboplatin and pemetrexed with or without pembrolizumab for advanced, non-squamous non-small-cell lung cancer: a randomised, phase 2 cohort of the open-label KEYNOTE-021 study. *Lancet Oncol.* 2016;17(11):1497-1508.
47. Lopes G, Wu Y-L, Kudaba I, et al. Pembrolizumab (pembro) versus platinum-based chemotherapy (chemo) as first-line therapy for advanced/metastatic NSCLC with a PD-L1 tumor proportion score (TPS) ≥ 1%: open-label, phase 3 KEYNOTE-042 study. *J Clin Oncol.* 2018;36(18_suppl):LBA4.
48. Paz-Ares LG, Luft A, Tafreshi A, et al. Phase 3 study of carboplatin-paclitaxel/nab-paclitaxel (Chemo) with or without pembrolizumab (Pembro) for patients (Pts) with metastatic squamous (Sq) non-small cell lung cancer (NSCLC). *J Clin Oncol.* 2018;36(15_suppl):105.
49. Gandhi L, Rodriguez-Abreu D, Gadgeel S, et al. Pembrolizumab plus chemotherapy in metastatic non-small-cell lung cancer. *N Engl J Med.* 2018;378(22):2078-2092.

© 2019 Yu Y et al. *JAMA Network Open.*
